# Combining alkali metals and zinc to harness heterometallic cooperativity in cyclic ester ring-opening polymerisation†

**DOI:** 10.1039/d0sc04705h

**Published:** 2020-10-12

**Authors:** Weronika Gruszka, Anna Lykkeberg, Gary S. Nichol, Michael P. Shaver, Antoine Buchard, Jennifer A. Garden

**Affiliations:** EaStCHEM School of Chemistry, University of Edinburgh Edinburgh EH9 3FJ UK j.garden@ed.ac.uk; School of Natural Sciences, Department of Materials, Henry Royce Institute, University of Manchester Manchester M13 9PL UK; Department of Chemistry, University of Bath Claverton Down Bath BA2 7AY UK

## Abstract

Heterometallic cooperativity is an emerging strategy to elevate polymerisation catalyst performance. Here, we report the first heterotrimetallic Na/Zn_2_ and K/Zn_2_ complexes supported by a ProPhenol ligand, which deliver “best of both” in cyclic ester ring-opening polymerisation, combining the outstanding activity (Na/K) and good control (Zn_2_) of homometallic analogues. Detailed NMR studies and density-functional theory calculations suggest that the Na/Zn_2_ and K/Zn_2_ complexes retain their heterometallic structures in the solution-state. To the best of our knowledge, the K/Zn_2_ analogue is the most active heterometallic catalyst reported for *rac*-lactide polymerisation (*k*_obs_ = 1.7 × 10^−2^ s^−1^), giving activities five times faster than the Na/Zn_2_ complex. These versatile catalysts also display outstanding performance in ε-caprolatone and δ-valerolactone ring-opening polymerisation. These studies provide underpinning methodologies for future heterometallic polymerisation catalyst design, both in cyclic ester polymerisation and other ring-opening (co)polymerisation reactions.

## Abstract 2

Cooperative heterotrimetallic Na/Zn_2_ and K/Zn_2_ complexes combine the excellent activities and control of the homometallic analogues, giving “best of both” in cyclic ester ring-opening polymerisation.

## Graphical abstract

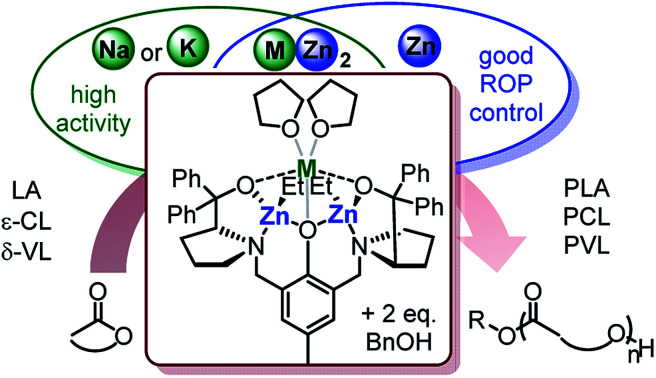

## Introduction

Cyclic ester ring-opening polymerisation (ROP) is an efficient method for producing aliphatic polyesters such as poly(lactic acid) (PLA), poly(ε-caprolactone) (PCL) and poly(δ-valerolactone) (PVL).^1–3^ The degradable and biocompatible properties of these polyesters have led to applications across packaging,^4^ electronics and medicine.^5^ ROP is dependent on the catalyst, and well-defined organometallic complexes have exerted excellent activities, selectivities and control over the polymer microstructure. The first examples comprised homoleptic homometallic alkoxides, *e.g.* Al(O^*i*^Pr)_3_, however bimetallic catalysts have recently gathered increased attention,^6^ with most examples based on biocompatible Al, Ca, Fe, K, Na, Ti and Zn metals.^7^ Despite the high activity of alkali metal catalysts,^8^ bimetallic zinc catalysts are generally more efficient at combining high activities with polymerisation control in ROP.^9–11^ We recently reported a highly active bimetallic zinc-benzoxide catalyst (**[LZn2OBn]**),^12^ based on the Trost ProPhenol ligand (**LH3**), for the controlled homo- and co-polymerisation of *rac*-lactide (*rac*-LA), ε-caprolactone (ε-CL) and *rac*-β-butyrolactone.

**Fig. 1:**
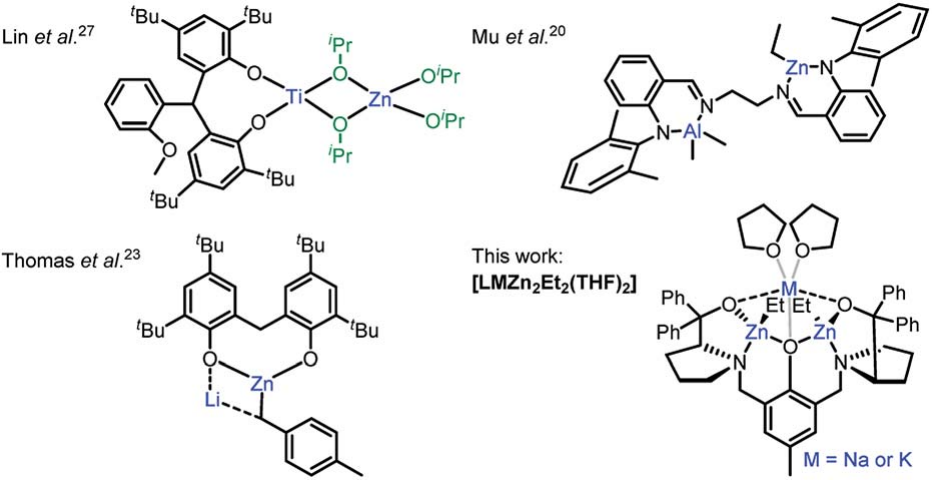
Heterometallic M/Zn complexes reported for cyclic ester ROP.

The activity and selectivity of homometallic species can be enhanced by introducing a heterometal into the same complex, which can result in heterometallic cooperativity. Inspired by nature, which has long utilised heterometallic metalloenzymes in biological transformations,^13,14^ chemists have observed unprecedented activity and selectivity enhancements with heterometallic complexes in metal–halogen exchange,^15^ C–H bond activation^16^ and olefin polymerisation.^17,18^ However, the concept remains underexplored in cyclic ester ROP despite heterometallic complexes with a M–O–M′ (M ≠ M′) framework (and thus intermetallic electronic communication *via* the O heteroatom) having the potential to enhance monomer coordination by increasing the metal Lewis acidity and accelerate propagation by enhancing the metal-alkoxide nucleophilicity.^17,19^ To date, the best performing heterometallic catalysts for LA and ε-CL ROP have generally comprised metals with large ionic radii and significant electronegativity differences between the metals, *e.g.* Al/Zn,^20^ La/Mg,^21^ Li/In,^22^ Li/Mg and Li/Zn,^23^ Li/Sm,^24^ Na/Sm,^25^ Sm/Al^26^ and Ti/Zn^27^ (Fig. 1). Combining Zn with electropositive alkali metals, which are highly active, inexpensive, earth abundant and non-toxic, is thus attractive from scientific, economic and environmental perspectives, yet remains underexplored.^7^ Herein, the synthesis and activity of novel heterometallic complexes **[LMZn2Et2(THF)2]** (Fig. 1, where M = Na or K) are reported for cyclic ester ROP.

## Results and discussion

### Homo- and hetero-metallic complex synthesis

**Scheme 1:**
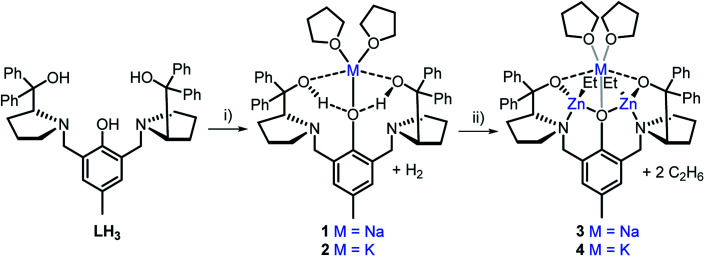
Synthesis of monometallic complexes **1** and **2** and heterometallic complexes **3** and **4**. Reagents and conditions: (i) 1.1 eq. MH, THF, R.T., 2 h; (ii) 2 eq. ZnEt_2_, THF, R.T., 1 h.

**Fig. 2:**
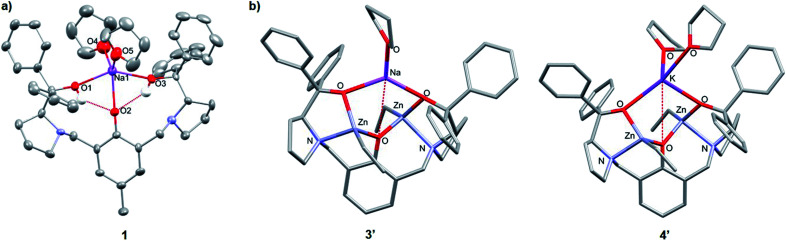
(a) Molecular structure of **1**. Ellipsoids set at 50% probability level. H atoms and co-crystallised THF have been omitted for clarity. Selected bond lengths (Å): Na1–O1 2.258(6), Na1–O2 2.355(5), Na1–O3 2.254(6), Na1–O4 2.278 (7), Na1–O5 2.258(7). Selected bond angles (°): O1–Na1–O2 70.6(2), O2–Na1–O3 73.7(2), O1–Na1–O4 90.2(3), O3–Na1–O5 96.1(2), O4–Na1–O5 119.6(3). (b) Molecular structures of **3′** and **4′** with the lowest free enthalpies computed by DFT (refer to Tables S6 and S13†).

The heterometallic precursors **[LH2Na(THF)2]** (**1**) and **[LH2K(THF)2]** (**2**) were synthesised *via* mono-deprotonation of the phenolic-O*H* of **LH3** with NaH or KH, respectively (Scheme 1), and characterised by NMR spectroscopy, mass spectrometry and elemental analysis (Fig. S1–S6†). ^1^H and DOSY NMR analysis indicated that **1** and **2** are centrosymmetric and mononuclear in THF-*d*_8_ solution (Fig. S2 and S5†). Crystals of **1** were obtained by cooling a 1 : 1 THF : toluene mixture to −34 °C, yet despite multiple attempts, the crystals of **2** obtained were unsuitable for X-ray crystallographic analysis. **1** was mononuclear in the solid-state, with pentacoordinate Na displaying a low *τ* value (*τ* = 0.41, Fig. 2a)^28^ suggesting that the structure is closer to a square pyramidal geometry than a trigonal bipyramidal geometry. The Na coordination sphere comprises a longer central Na1–O2 bond (2.355(5) Å) and four shorter dative bonds, two from the benzylic-*O*H [Na1–O1 (2.258(6) Å) and Na1–O3 (2.254(6) Å)] and two from THF [Na1–O4 (2.278(7) Å) and Na1–O5 (2.258(7) Å)]. In contrast to the solution-state, **1** was non-centrosymmetric in the solid-state, as evidenced by the tetragonal space group (*P*4_3_).

Although complex **1** features two benzylic O*H* groups that are acidified through hydrogen bonding to the phenolic O, these groups were not deprotonated with further equivalents of NaH (≤3 eq. in total). This suggests that the product selectivity is influenced by both the p*K*_a_ of the O*H* groups and the ionic radii of the alkali metals. Notably, Na^+^ and K^+^ are significantly larger (102 and 138 pm, respectively) than Li^+^ (76 pm),^29^ and indeed, metalation of **LH3** with ^*n*^BuLi was less selective. NMR spectroscopic studies revealed two products, one symmetric (attributed to lithiation of the phenol-O*H*) and one asymmetric (lithiation of the benzylic O*H*). However, **1** and **2** were selectively deprotonated with 2 eq. of ZnEt_2_, yielding complexes **[LNaZn2Et2(THF)2]** (**3**) and **[LKZn2Et2(THF)2]** (**4**) (Scheme 1), which were characterised by NMR spectroscopy, mass spectrometry and elemental analysis (Fig. S7–S12†). The centrosymmetric solution-state structure of **1** and **2** was also prominent in **3** and **4**, suggesting that each Et_2_Zn deprotonates one benzylic O*H* and retains one ethyl group (Fig. S7 and S10†). DOSY NMR analysis suggests that **3** and **4** are both monomeric in the solution state (Fig. S8 and S11†). Unfortunately, attempts to isolate single crystals of **3** and **4** suitable for X-ray diffraction studies proved unsuccessful. However, density-functional theory (DFT) calculations confirmed the heterometallic structures and stability of **3** and **4** (refer to ESI†). The calculations suggest that the *R*,*R* configuration of the N atoms observed in the molecular structure of **1** is likely retained with **3′** and **4′** (′ denotes computationally modelled structures, see ESI†), resulting in the two Zn-Et moieties facing in opposite directions relative to the phenol ring plane (Fig. 2b). However, ligand rearrangement to a *meso* (*R*,*S*) configuration at the N atoms, with Zn-Et groups facing in the same direction, was found to be only slightly endergonic for both **3′** (+2.0 kcal mol^−1^) and **4′** (+7.7 kcal mol^−1^) (Tables S6 and S13†).

### *Rac*-LA polymerisation: heterometallic *vs.* homometallic catalysis

Complexes **3** and **4** were found to be highly efficient initiators for *rac*-LA ROP with 2 eq. of benzyl alcohol (BnOH, Table 1). Complex **4** exhibited an exceptional polymerisation rate of *k*_obs_ = 1.7 × 10^−2^ s^−1^ in THF solvent at room temperature (R.T., Fig. S17†), converting 60 eq. of *rac*-LA in just 20 seconds. Not only is **4** five times faster than **3** (*k*_obs_ = 3.2 × 10^−3^ s^−1^) but it is, to the best of our knowledge, the most active heterometallic catalyst system reported for LA ROP and the first heterometallic K/Zn catalyst reported for cyclic ester ROP. Previously, some of the most active heterometallic catalysts for LA ROP were Li/Zn and Li/Mg complexes supported by a bis(phenol) ligand,^23^ which respectively converted 62 eq. and 88 eq. *rac*-LA in 15 min at R.T. in the presence of 1 eq. neopentyl alcohol. Complexes **3** and **4** (with 2 eq. of BnOH) also displayed activity for *rac*-LA ROP at 0.2–0.5 mol% catalyst loadings, generating PLA with *M*_n_ of up to 12 100 g mol^−1^ (Table S1†).

**Table 1:** ROP of *rac*-LA catalysed by complexes **1–4**, **[BnONa]**, **[BnOK]** and **[LZn2OBn]**^a^

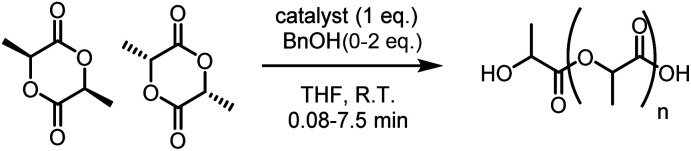						
Entry	Cat.	Time (min)	Conv.^b^ (%)	*M*_n,obs_ c (Da)	*M*_n,calc_ d (Da)	*Đ* c
1^e^^,^^f^	**3**	2.5	12	—	—	—
2	**3**	0.33	47	2100	3400	1.3
3	**3**	2.5	71	3000	5100	1.2
4	**3**	7.5	86	3900	6200	1.4
5^e^^f^	**4**	2.5	13	—	—	—
6	**4**	0.08	45	1900	3300	1.7
7	**4**	0.33	60	2500	4300	1.4
8	**4**	1.25	81	3900	5800	1.4
9	**4**	2	93	4300	6700	1.4
10^e^	**1**	1.25	79	14 800	11 400^g^	4.1
11^e^	**[BnONa]**	1.25	88	20 400	12 700^g^	2.7
12^e^	**2**	0.33	72	7300	10 400^g^	4.3
13^e^	**[BnOK]**	0.25	94	13 100	13 600^g^	1.9
14^e^	**[LZn2OBn]**	7.5	17	—	—	—
15^e^^,^^h^	**[BnOK] + [LZn2OBn]**	0.25	99	8300	7100	1.6

a 100 : 1 : 2 LA : cat : BnOH, [LA] = 1 M in THF.

b Calculated using ^1^H NMR spectroscopy.

c Determined by GPC using polystyrene standards in THF. Values corrected by Mark–Houwink factor (0.58).^31^

d Calculated from the monomer conversion *M*_n,calc_ = M_0_ × ([M]/[I]) × conversion assuming 2 chains per catalyst.

e No BnOH used.

f Polymerisations run at 60 °C.

g Calculated from the monomer conversion *M*_n,calc_ = M_0_ × ([M]/[I]) × conversion assuming 1 chain per catalyst.

h **[BnOK]** generated *in situ* from KH and BnOH before adding **[LZn2OBn]**.

The polymerisations with **3** or **4** (and 2 eq. BnOH) were controlled with a linear relationship between *M*_n_ and monomer conversion (Table 1, Fig. S18 and S19†). The discrepancy between the observed and calculated *M*_n_ values was attributed to transesterification reactions, as evidenced by MALDI-ToF analysis (refer to ESI†). End-group analysis revealed the expected α-benzoxy, ω-hydroxy end-capped polymer chains. However, unlike related homometallic Trost ProPhenol catalysts (**[(LH)2Zr]**^30^ and **[LZn2OBn]**^12^) no ligand end groups were detected with complexes **3** and **4**; this improved control may arise from the increased chelate stability and steric congestion of **3** and **4**. Similarly to homometallic **[LZn2OBn]**,^12^ the PLA generated from *rac*-LA was either atactic (maximum *P*_i_ = 0.53 with **3**, Table S1†) or showed a modest isotactic bias (maximum *P*_i_ = 0.62 with **4**). Kinetic studies of l-LA ROP (Fig. S34†) indicated that **4** is twice as active in *rac*-LA ROP (*k*_obs_ = 1.7 × 10^−2^ s^−1^) than l-LA ROP (*k*_obs_ = 7.8 × 10^−3^ s^−1^), whereas **3** displays similar polymerisation rates for *rac*- and l-LA (*k*_obs_ = 3.2 × 10^−3^ and 2.7 × 10^−3^ s^−1^, respectively). These results suggest that while **3** likely has a similar degree of preference for d- and l-LA enchainment, **4** might display a slight preference for d-LA coordination and insertion, resulting in a modest isotactic bias. Notably, only trace *rac*-LA (<13%) was converted in the absence of BnOH co-initiator (Table 1, entries 1 and 5); these conversions were only mildly improved by the addition of 1 eq. BnOH to give 15% conversion with **3** after 5 min, and 20% conversion with **4** after 1.25 min (THF at R.T, Table S1,† entries 12 and 25). The dramatically reduced activity in the presence of 1 eq. BnOH suggests that **3** and **4** are unlikely to operate *via* an activated monomer mechanism as 2 eq. BnOH are required to efficiently initiate the proposed coordination-insertion mechanism. Complexes **3** and **4** also remained active under immortal polymerisation conditions (10 eq. BnOH, Table S1,† entries 13 and 26).

Complexes **3** and **4** were benchmarked against homometallic complexes **1–2**, **[BnONa]**, **[BnOK]** and **[LZn2OBn]** in THF (Table 1). The alkali metal analogues were highly active but poorly controlled; the MALDI-ToF data shows transesterified ω-hydroxy end-capped and cyclic PLA (see ESI†). **[BnONa]** and **[BnOK]** also displayed poor solubility in THF and toluene, emphasising a potential benefit of heterometallic initiators, which are often more soluble than the homometallic counterparts. Although **[LZn2OBn]** displays good activities in toluene,^12^ the activity is diminished in THF (entry 14, Table 1). In contrast, **3** and **4** gave improved activities in THF, which was attributed to the Lewis acidic alkali metals, particularly the larger K^+^ in **4** (*vs.* Na^+^ in **3**), providing additional coordination sites, thus preventing competitive THF/LA coordination. Indeed, DFT calculations suggest a slight preference for coordination of 2 eq. THF to **4′***vs.* 1 eq. THF to **3′** (Fig. 2, Tables S6 and S13†), even if coordination of 2 eq. THF to both **3** and **4** was observed by NMR analysis. Complexes **3** and **4** were also significantly faster than *in situ* generated **[LZn2OBn]** in *rac*-LA ROP in toluene at 60 °C (Table S3†), with **3** and **4** converting 89 eq. and 86 eq. *rac*-LA in 2.5 and 1 min, respectively (*vs.* 87 eq. in 10 min with *in situ* generated **[LZn2OBn]**). The activity and control differences between **3–4** and their homometallic analogues suggest cooperative interactions between Na/K and Zn_2_.

### Reactivity insights: experimental and computational studies

The *in situ* generation of **[LNaZn2(OBn)2(THF)2]** (**5**) and **[LKZn2(OBn)2(THF)2]** (**6**) from **3** or **4** and 2 eq. BnOH was investigated by NMR analysis in THF-*d*_8_, which indicated the rapid loss of BnO*H* and the formation of ethane (0.85 ppm) and new centrosymmetric complexes with OBn co-ligands (Fig. S44 and S45†). Notably, the Zn-Et groups of **3** remained intact in the presence of 10 eq. *rac*-LA until the addition of 2 eq. BnOH whereupon the Zn-Et groups disappeared and PLA was rapidly formed (Fig. S46†). DOSY NMR analysis of *in situ* generated **5** and **6** confirmed that the OBn co-ligands and **L** were part of the same complex (Fig. S47 and S48†). DFT calculations suggested **5′** and **6′** conserve the *R*,*R* ligand stereochemistry at the N atoms (*vide supra*) but with ligand rearrangement to a *meso* (*R*,*S*) configuration also possible under the polymerisation conditions (Tables S9 and S14†). The *in situ* dissociation of **5** and **6** in THF to **[LZn2OBn]** and **[BnONa]** or **[BnOK]**, respectively, was deemed unlikely based on NMR and DFT calculations (Fig. S49, S50 and Table S10†). The reaction of **1** and **2** with 1 eq. BnOH was also investigated but gave no reaction *i.e.* no **[BnONa]** or **[BnOK]** was formed (Fig. S51†). These findings suggest that the rearrangement of **3** and **4** to homometallic species is unlikely under the polymerisation conditions. This was further supported by monitoring the reaction of **[LZn2OBn]** with 1 eq. of *in situ* generated **[BnOK]** in THF-*d*_8_ by ^1^H NMR, which also generated **6** (Fig. S52†). Testing a 1 : 1 **[BnOK]** : **[LZn2OBn]** mixture in *rac*-LA ROP gave excellent activity in both THF and toluene (Tables 1 and S3†), albeit with reduced polymerisation control (*Đ* = 1.6–2.0) *vs.***[LZn2OBn]** and **4** (*Đ* < 1.5). Similarly to LiCl addition boosting the activity of conventional Grignard reagents by forming heterometallic Turbo-Grignard reagents,^32^ our findings suggest that addition of an alkali metal alkoxide to a bis-Zn complex may provide a simple yet effective strategy for improving the performance of homometallic ROP initiators. However, in this case, the optimal balance between the polymerisation activity and control was achieved with *in situ* generation of **5** and **6***via* alcoholysis of **3** and **4** (Table 1, *vide infra*).

Coordination of 1–2 eq. l-LA to **5′** and **6′** was modelled by DFT (see ESI†); these reactions were either neutral or slightly exergonic. The most stable structures feature the *R*,*R* ligand configuration at the N atoms and one l-LA coordinated to the alkali metal, although coordination of two l-LA may also be accessible under polymerisation conditions. While no significant differences in structural and l-LA coordination preferences were found between **5′** and **6′**, the data suggests that coordination of two l-LA is more accessible for **6′** (*vs.***5′**), in line with the increased ionic radius of K^+^. The activity differences between **3** and **4** (with 2 eq. BnOH) may thus have a kinetic origin; this requires modelling of ROP transition states which are currently under investigation in our laboratories.

Previous studies showed that isolated **[LZn2OBn]** gave a ten-fold activity increase *vs.* the *in situ* generated complex,^12^ and so the isolation of **5** and **6** was investigated. However, the isolated heterometallic species showed reduced activity in *rac*-LA ROP compared to the *in situ* generated analogues (with 2 eq. BnOH); isolated **5** was approximately six times slower than **3**, and **6** gave half the rate of **4** (THF, R.T.). In contrast to *in situ* generated **5** and **6**, DOSY NMR analysis of isolated **5** and **6** (Fig. S54 and S55†) suggested formation of a two-component mixture involving higher MW species (approx. 826 Da in both cases) and lower MW species comprising OBn anions (252 Da for **5** and 298 Da for **6**). This may explain the reduced activity of the isolated species, as steric congestion around the metals in **5** and **6** could decrease the stability over time in THF (1 h at R.T.), leading to the formation of (mixed) metal-OBn aggregates. It is also plausible that increased concentration upon solvent removal for product isolation leads to formation of different structures, as Lewis donor solvents are well-known to influence aggregation states of organometallic complexes. Importantly, on the timescale of the *in situ* generated polymerisations (<7.5 min at R.T. in THF), there was no evidence of decomposition by NMR analysis.

### Catalyst scope

Complex **3** (with 2 eq. BnOH) is also extremely active in ε-CL and δ-valerolactone (δ-VL) ROP (Table S4†), converting 53 eq. ε-CL and 99 eq. δ-VL in just 5 s at R.T. Notably, **3** converts up to 760 eq. ε-CL in 4 min at R.T., producing PCL with *M*_n_ up to 19 600 g mol^−1^. While complex **4** converted 94 eq. δ-VL in 5 s, it was less active than **3** in ε-CL ROP (Table S4†), which contrasts with the higher activity of **4** (*vs.***3**) in *rac*-LA ROP. The more Lewis basic character of ε-CL *vs.* δ-VL and LA (based on the FT-IR carbonyl shifts: *ν*(C=O) = 1732 cm^−1^ for ε-CL, 1747 cm^−1^ for δ-VL and 1770 cm^−1^ for LA)^33^ may promote decomposition/rearrangement of **4**; this may be more likely with **4** than **3** due to the larger and more electropositive K^+^ centre facilitating ε-CL coordination.

## Conclusions

In summary, two novel heterometallic complexes **3** and **4** were reported to be highly active in *rac*-LA, ε-CL and δ-VL ROP with 2 eq. BnOH. Complexes **3** and **4** outperform their homometallic counterparts, combining high activities with good polymerisation control. To the best of our knowledge, **4**/BnOH (2 eq.) is the fastest heterometallic catalytic system for *rac*-LA ROP reported to date. These rate enhancements demonstrate the benefit of combining metals known to be highly active in cyclic ester ROP (Zn) with abundant, inexpensive and non-toxic alkali metals (Na and K). When bridged through a heteroatom, electronic communication between heterometals can alter the properties of each metal through an “ate” activation. While alkali metals are known to boost the reactivity of Zn towards C–H activation,^34^ our studies suggest that this concept can also be translated to cyclic ester ROP. The activity enhancements observed may arise from increased Lewis acidity of the more electropositive metal (Na/K) and labilisation of the M–OR bonds around the more electrophilic metal(s) (Zn). Heterometallic catalysts remain underexplored in ROP and offer a promising area for further investigation.

## Conflicts of interest

There are no conflicts to declare.

## Supplementary Material

SC-011-D0SC04705H-s001

SC-011-D0SC04705H-s002
