# Supplementary material for: Combining alkali metals and zinc to harness heterometallic cooperativity in cyclic ester ring-opening polymerisation
Source: Chem Sci. 2020 Oct 12;11(43):11785–90. doi: 10.1039/d0sc04705h (PMC8162475; doi:10.1039/d0sc04705h)
Supplement: SC-011-D0SC04705H-s001 [file SC-011-D0SC04705H-s001.pdf]

Electronic Supplementary Material (ESI) for Chemical Science.
This journal is © The Royal Society of Chemistry 2020

Supporting Information

Combining Alkali Metals and Zinc to Harness Heterometallic Cooperativity in Cyclic Ester Ring-Opening Polymerisation

Weronika Gruszka,[a] Anna Lykkeberg,[a] Gary S. Nichol,[a] Michael P. Shaver,[b] Antoine Buchard,[c] Jennifer A. Garden*[a]

[a] *EaStCHEM School of Chemistry, University of Edinburgh, Edinburgh, EH9 3FJ, UK*

[b] *School of Natural Sciences, Department of Materials and Henry Royce Institute, University of Manchester, Manchester, M13 9PL, UK*

[c] *Department of Chemistry, University of Bath, Claverton Down, Bath, BA2 7AY, UK*

E-mail: j.garden@ed.ac.uk

Table of Contents

General experimental details 6

General computational details 6

Synthesis and characterisation of complex **1** 7

Synthesis and characterisation of complex **2** 7

Synthesis and characterisation of complex **3** 8

Synthesis and characterisation of complex **4** 8

Synthesis and characterisation of **[BnONa]** 9

Synthesis and characterisation of **[BnOK]** 9

General experimental procedure for the ring-opening polymerisation of *rac*-LA in toluene 9

General experimental procedure for the ring-opening polymerisation of LA in THF 9

General experimental procedure for the ring-opening polymerisation of ε-CL 10

General experimental procedure for the ring-opening polymerisation of δ-VL 10

Figure S1. ^{1}H and ^{13}C NMR spectra of complex **1** in THF-d_8 (298 K) 11

Figure S2. DOSY NMR spectrum of complex **1** in THF-d_8 (298 K) 12

Figure S3. MALDI-ToF spectrum of complex **1** 13

Figure S4. ^{1}H and ^{13}C NMR spectra of complex **2** in THF-d_8 (298 K) 14

Figure S5. DOSY NMR spectrum of complex **2** in THF-d_8 (298 K) 15

Figure S6. APPI-MS spectrum of complex **2** 16

Figure S7. ^{1}H and ^{13}C NMR spectra of complex **3** in THF-d_8 (298 K) 17

Figure S8. DOSY NMR spectrum of complex **3** in THF-d_8 (298 K) 18

Figure S9. APPI-MS spectrum of complex **3**........19
Figure S10. ^{1}H and ^{13}C NMR spectra of complex **4** in THF-d_8 (298 K) 20
Figure S11. DOSY NMR spectrum of complex **4** in THF-d_8 (298 K) 21
Figure S12. APPI-MS spectrum of complex **4**........22
Figure S13. ^{1}H and ^{13}C NMR spectra of **[BnONa]** in THF-d_8 (298 K) 23
Figure S14. DOSY NMR spectrum of **[BnONa]** in THF-d_8 (298 K) 24
Figure S15. ^{1}H and ^{13}C NMR spectra of **[BnOK]** in THF-d_8 (298 K) 25
Figure S16. DOSY NMR spectrum of **[BnOK]** in THF-d_8 (298 K) 26
Table S1. Full table of results for the ROP of *rac*-LA catalysed by complexes **3** and **4** in the presence of 2 eq. of BnOH in THF at R.T. 27
Figure S17. Plots of ln([LA]$_0$/[LA]$_t$) *vs*. time (min) for ROP of *rac*-LA with complexes **3** and **4** ([LA] = 1 M in THF, R.T., 2 eq. BnOH used) 27
Figure S18. Comparison between experimental and calculated M_n values and dispersity values at increasing conversions of *rac*-LA in presence of complex **3** + 2 eq. BnOH in THF at R.T. 28
Figure S19. Comparison between experimental and calculated M_n values and dispersity values at increasing conversions of *rac*-LA in presence of complex **4** + 2 eq. BnOH in THF at R.T. 28
Figure S20. Example GPC trace of PLA generated in presence of complex **3** + 2 eq. BnOH in THF at R.T. 29
Figure S21. Example GPC trace of PLA generated in presence of complex **4** + 2 eq. BnOH in THF at R.T. 29
Figure S22. MALDI-TOF spectrum of PLA resulting from 47 % conversion of *rac*-LA in the presence of complex **3** + 2 eq. BnOH (THF, R.T.) 30
Figure S23. MALDI-TOF spectrum of PLA resulting from 71 % conversion of *rac*-LA in the presence of complex **3** + 2 eq. BnOH (THF, R.T.) 31
Figure S24. MALDI-TOF spectrum of PLA resulting from 86 % conversion of *rac*-LA in the presence of complex **3** + 2 eq. BnOH (THF, R.T.) 32
Figure S25. MALDI-TOF spectrum of PLA resulting from 53 % conversion of *rac*-LA in the presence of complex **4** + 2 eq. BnOH (THF, R.T.) 33
Figure S26. MALDI-TOF spectrum of PLA resulting from 67 % conversion of *rac*-LA in the presence of complex **4** + 2 eq. BnOH (THF, R.T.) 34
Figure S27. MALDI-TOF spectrum of PLA resulting from 93 % conversion of *rac*-LA in the presence of complex **4** + 2 eq. BnOH (THF, R.T.) 35
Figure S28. MALDI-TOF spectrum of PLA resulting from 79 % conversion of *rac*-LA in the presence of homometallic complex **1** (THF, R.T.) 36
Figure S29. MALDI-TOF spectrum of PLA resulting from 72 % conversion of *rac*-LA in the presence of homometallic complex **2** (THF, R.T.) 37
Figure S30. MALDI-TOF spectrum of PLA resulting from 88 % conversion of *rac*-LA in the presence of **[BnONa]** (THF, R.T.) 38
Figure S31. MALDI-TOF spectrum of PLA resulting from 94 % conversion of *rac*-LA in the presence of **[BnOK]** (THF, R.T.) 39

Table S2. ROP of *rac*-LA catalysed by complexes **3** and **4** in the presence of 2 eq. of BnOH in THF at 60 °C 40

Figure S32. Plot of $\ln([LA]_0/[LA]_t)$ *vs*. time (min) for ROP of *rac*-LA with complex **3** and 2 eq. BnOH ([LA] = 1 M in THF, 60 °C) 40

Figure S33. Comparison between experimental and calculated M_n values and dispersity values at increasing conversions of *rac*-LA in the presence of complex **3** + 2 eq. BnOH in THF at 60 °C 41

Figure S34. Plot of $\ln([LA]_0/[LA]_t)$ *vs*. time (min) for ROP of L-LA with complexes **3** and **4** and 2 eq. BnOH ([LA] = 1 M in THF, R.T.) 42

Table S3. ROP of *rac*-LA catalysed by complexes **3** and **4** in the presence of 2 eq. of BnOH in toluene at 60 °C 42

Figure S35. Plot of $\ln([LA]_0/[LA]_t)$ *vs*. time (min) for ROP of *rac*-LA with complex **3** and 2 eq. BnOH ([LA] = 1 M in toluene, 60 °C) 43

Figure S36. Comparison between experimental and calculated M_n values and dispersity values at increasing conversions of *rac*-LA in the presence of complex **3** + 2 eq. BnOH in toluene at 60 °C 43

Figure S37. Example GPC trace of PLA generated in the presence of complex **3** + 2 eq. BnOH in toluene at 60 °C 44

Figure S38. Example GPC trace of PLA generated in the presence of complex **4** + 2 eq. BnOH in toluene at 60 °C 44

Figure S39. MALDI-TOF spectrum of PLA resulting from 35 % conversion of *rac*-LA in the presence of complex **3** + 2 eq. BnOH (toluene, 60 °C) 45

Figure S40. MALDI-TOF spectrum of PLA resulting from 56 % conversion of *rac*-LA in the presence of complex **3** + 2 eq. BnOH (toluene, 60 °C) 46

Figure S41. MALDI-TOF spectrum of PLA resulting from 89 % conversion of *rac*-LA in the presence of complex **3** + 2 eq. BnOH (toluene, 60 °C) 47

Figure S42. MALDI-TOF spectrum of PLA resulting from 68 % conversion of *rac*-LA in the presence of complex **4** + 2 eq. BnOH (toluene, 60 °C) 48

Figure S43. MALDI-TOF spectrum of PLA resulting from 86 % conversion of *rac*-LA in the presence of complex **4** + 2 eq. BnOH (toluene, 60 °C) 49

Figure S44. ^{1}H NMR spectrum of *in situ* generated complex **5** in THF-d_8 (298 K) 50

Figure S45. ^{1}H NMR spectrum of *in situ* generated complex **6** in THF-d_8 (298 K) 51

Figure S46. An overlay of ^{1}H NMR spectra of complex **3** in the presence and absence of 10 eq. *rac*-LA and 2 eq. BnOH in THF-d_8 (298 K) 52

Figure S47. DOSY NMR spectrum of *in situ* generated complex **5** in THF-d_8 (298 K) 53

Figure S48. DOSY NMR spectrum of *in situ* generated complex **6** in THF-d_8 (298 K) 54

Figure S49. An overlay of ^{1}H NMR spectra of complexes **3**, *in situ* generated **5** and **[BnONa]** in THF-d_8 (298 K) 55

Figure S50. An overlay of ^{1}H NMR spectra of complexes **4**, *in situ* generated **6** and **[BnOK]** in THF-d_8 (298 K) 55

Figure S51. An overlay of ^{1}H NMR spectra of complexes **1** and **2** in the presence and absence of 1 eq. BnOH in THF-d_8 (298 K) 56

Figure S52. An overlay of ^{1}H NMR spectra of **[BnOK]**, **[LZn$_2$OBn]**, *in situ* generated complex **6** (from the reaction of **4** with 2 eq. BnOH) and product of the *in situ* reaction between **[LZn$_2$OBn]** and **[BnOK]** in THF-d_8 (298 K) ... 57

Figure S53. An overlay of ^{1}H NMR spectra of *in situ* generated and isolated complexes **5** and **6** in THF-d_8 (298 K) ... 58

Figure S54. DOSY NMR spectrum of the 2-component mixture attained upon the attempted isolation of complex **5** in THF-d_8 (298 K) ... 59

Figure S55. DOSY NMR spectrum of the 2-component mixture attained upon the attempted isolation of complex **6** in THF-d_8 (298 K) ... 60

Table S4. ROP of ε-CL and δ-VL with complexes **3** and **4** and 2 eq. of BnOH in THF at R.T. ... 60

Figure S56. Example GPC trace of PCL generated in the presence of complex **3** + 2 eq. BnOH in THF at R.T. ... 61

Figure S57. Example GPC trace of PVL generated in the presence of complex **3** + 2 eq. BnOH in THF at R.T. ... 61

Figure S58. Example GPC trace of PCL generated in the presence of complex **4** + 2 eq. BnOH in THF at R.T. ... 62

Figure S59. Example GPC trace of PVL generated in the presence of complex **4** + 2 eq. BnOH in THF at R.T. ... 62

Figure S60. MALDI-TOF spectrum of PCL resulting from 53 % conversion of ε-CL in the presence of complex **3** + 2 eq. BnOH (THF, R.T.) ... 63

Figure S61. MALDI-TOF spectrum of PVL resulting from 99 % conversion of δ-VL in the presence of complex **3** + 2 eq. BnOH (THF, R.T.) ... 64

Figure S62. MALDI-TOF spectrum of PVL resulting from 94 % conversion of δ-VL in the presence of complex **4** + 2 eq. BnOH (THF, R.T.) ... 65

Table S5. Crystallographic data and collection and refinement details for complex **1** ... 66

Table S6. Free enthalpies computed for various conformers of complex **3’** ... 67

Figure S63. Molecular structure of complex **3’-4** ... 68

Figure S64. Thermodynamics of protonation of complex **3’** with 1 eq. BnOH ... 69

Table S7. Free enthalpies computed for various conformers of complex **3’-n*** ... 70

Figure S65. Thermodynamics of protonation of complex **3’** with 2 eq. BnOH ... 71

Table S8. Free enthalpies computed for the protonation of complex **3’** into complex **5’** ... 72

Table S9. Free enthalpies computed for various conformers of complex **5’** ... 73

Figure S66. Molecular structure of complex **5’-4** ... 74

Figure S67. Thermodynamics of possible rearrangement reactions of complex **5’** ... 75

Table S10. Free enthalpies computed for various homo- and heterometallic rearrangements of complex **5’** ... 76

Table S11. Free enthalpies computed for various structures of complex **5’-n-LLA** ... 78

Table S12. Comparative free enthalpies of various structures of complex **5’-n-LLA** ... 79

Figure S68. Molecular structure of complex **5’-4-LLA** ... 80

Table S13. Free enthalpies computed for various conformers of complex **4'** 81
Figure S69. Molecular structure of complex **4'-1** 82
Table S14. Free enthalpies computed for various conformers of complex **6'** 83
Figure S70. Molecular structure of complex **6'-1** 84
Table S15. Free enthalpies computed for various structures of complex **6'-n-LLA** 85
Table S16. Comparative free enthalpies of various structures of complex **6'-n-LLA** 86
Figure S71. Molecular structure of complex **6'-4-LLA** 87
Table S17. Comparison of selected bond lengths in DFT and X-ray structures of **[LZn$_2$(4-nitrophenol)]** 88
Table S18. Comparison of selected bond angles in DFT and X-ray structures of **[LZn$_2$(4-nitrophenol)]** 88
Figure S72. Molecular structure of complex **[LZn$_2$(4-nitrophenol)]** 89
References 90

General experimental details

All manipulations requiring inert conditions were performed under an argon atmosphere using standard Schlenk techniques or in a glove box. All reagents and solvents were obtained from Sigma-Aldrich, Fischer Scientific, Honeywell or Acros Organics and were used without further purification unless described otherwise. Dry THF, toluene and hexane were collected from a solvent purification system (Innovative Technologies), dried over activated 4 Å molecular sieves and stored under argon. THF-d_8 and toluene-d_8 NMR solvents for NMR were degassed by three freeze-pump-thaw cycles and stored over activated 4 Å molecular sieves under argon. *Rac*-lactide (*rac*-LA) and L-lactide (L-LA) were purified by double recrystallisation from toluene and sublimation. γ-valerolactone (γ-VL), ε-caprolactone (ε-CL), δ-valerolactone (δ-VL) and benzyl alcohol (BnOH) were dried over CaH_2 and distilled under reduced pressure prior to use. ^1H, ^{13}C and 2D NMR (COSY, HSQC and DOSY) spectra were recorded on a Bruker AVA500, PRO500, AVA400 and AVA600 spectrometers at 298 K at 400 MHz, 500 MHz and 600 MHz and referenced to the residual solvent peaks (^1H: δ 3.58 for THF-d_8 and δ 2.08 for toluene-d_8, ^{13}C: δ 67.21 for THF-d_8 and δ 137.48 for toluene-d_8). The reported DOSY masses (to the nearest whole number) and aggregation states were determined by comparison to a calibration plot made with a range of standards (hexamethyldisilazane (HMDSH), $Zn(HMDS)_2$, β-diketiminate ligand (BDIH) and (BDI)Zn(HMDS)) with molecular weights varying from 161.4 to 643.4 g mol^{-1} in THF-d_8.[1] APPI-MS analysis was performed using a Bruker Daltonics 12T SolariX Fourier Transform Ion Cyclotron Resonance Mass Spectrometer using atmospheric pressure photoionisation (APPI). Single crystal X-ray diffraction data were collected on a Rigaku Oxford Diffraction SuperNova diffractometer fitted with an Atlas CCD detector with Cu-Kα radiation (λ = 1.5418 Å). Crystals were mounted under Paratone on MiTeGen loops. The structures were solved by direct methods using SHELXS and refined by full-matrix least-squares on F^2 using SHELXL interfaced through Olex2.[2,3] GPC analyses of the filtered polymer samples were carried out in GPC grade THF at a flow rate of 1 mL min^{-1} at 35 °C on a 1260 Infinity II GPC/SEC single detection system with mixed bed C PLgel columns (300 x 7.5 mm). MALDI-TOF MS analyses were performed using a Bruker Daltonics UltrafleXtreme™ MALDI-TOF/TOF MS instrument. The sample to be analysed, dithranol matrix and KI (cationising agent) were dissolved in THF at 10 mg mL^{-1} and the solutions were mixed in a 2:2:1 volume ratio, respectively. A droplet (2 µL) of the resultant mixture was spotted on to the sample plate and submitted for MALDI-TOF MS analysis.

General computational details

All density-functional theory (DFT) calculations were performed on the University of Bath High Performance Computing service (Balena Cluster) and were performed using the Gaussian09 suite of codes (revision D.02).[4] Geometries were fully optimised without any symmetry or geometry

constraints. The nature of all the stationary points as minima was verified by calculations of the vibrational frequency spectrum at 298 K and characterised by no imaginary mode. Only the intermediates of lowest free enthalpy found are reported here. Free enthalpies were calculated within the harmonic approximation for vibrational frequencies. DFT Optimisation was carried out using the ωB97XD long-range corrected (LC) hybrid functional developed by Chai and Head-Gordon that includes an empirical dispersion correction.[5,6] The 6-31++g(d,p) basis set was used for Na, K, N, O, Zn atoms and the 6-31g(d) basis set was used for C and H atoms. Solvent effects in THF were considered using conductor-like polarisable continuum model (CPCM).[7,8] In order to facilitate DFT optimisations, calculations were carried out on a model version of the Trost ProPhenol ligand used experimentally, using no methyl group in the *para* position of the phenol ring. Such computational structures have therefore been augmented with the prime symbol. Full coordinates for all the stationary points, together with computed energies and vibrational frequency data, are available *via* the corresponding Gaussian 09 output files and calculation spreadsheet, stored in the open-access digital repository, DOI:10.6084/m9.figshare.12688109.

Synthesis and characterisation of complex 1

(*S,S*)-(+)-2,6-bis[2-(hydroxydiphenylmethyl)-1-pyrrolidinyl-methyl]-4-methylphenol (1.00 g, 1.57 mmol) was weighed into a Schlenk flask and dissolved in dry THF (20 mL) in the glove box. NaH (41.4 mg, 1.73 mmol) was slowly added into the ligand solution. The resulting mixture was stirred for 2 h at ambient temperature under an argon atmosphere in the glove box. THF was subsequently removed *in vacuo* resulting in a pale yellow powder (1.10 g, 87 %). Single crystals suitable for X-ray diffraction analysis were obtained *via* cooling down in a 1:1 mixture of THF and toluene to -34 °C. ^{1}H NMR (500 MHz, THF-d_8) δ 9.96 (s, 2 H), 7.57 (dd, 4 H), 7.34 (dd, 4 H), 7.28 (t, 4 H), 7.19 (t, 2 H), 7.02-7.05 (m, 6 H), 6.57 (s, 2 H), 4.48 (d, 2 H), 3.81 (dd, 2 H), 3.60-3.63 (m, 8 H), 3.08 (d, 2 H), 2.68 (dt, 2 H), 2.32 (dt, 2 H), 2.09 (s, 3 H), 1.91-1.99 (m, 2 H), 1.76-1.79 (m, 8 H), 1.57-1.62 (m, 2 H), 1.24-1.32 (m, 2 H), 0.31-0.40 (m, 2 H). ^{13}C NMR (126 MHz, THF-d_8) δ 166.14, 148.90, 148.06, 130.24, 129.11, 128.32, 128.16, 127.75, 127.08, 126.90, 126.21, 116.37, 70.84, 68.03, 61.67, 55.18, 30.53, 26.20, 24.08, 20.63.
Elemental analysis: calculated: C, 76.09; H, 7.64; N, 3.48 %. Found: C, 75.99; H, 7.54; N, 3.41 %.
m/z (MALDI-ToF): 683.4 $[LH_2Na + Na]^+$ (calc: 683.3)

Synthesis and characterisation of complex 2

(*S,S*)-(+)-2,6-bis[2-(hydroxydiphenylmethyl)-1-pyrrolidinyl-methyl]-4-methylphenol (1.00 g, 1.57 mmol) was weighed into a Schlenk flask and dissolved in dry THF (20 mL) in the glove box. KH (69.3 mg, 1.73 mmol) was slowly added into the ligand solution. The resulting mixture was stirred for 2 h at ambient temperature under an argon atmosphere in the glove box. THF was subsequently removed *in vacuo* resulting in a pale orange powder (0.84 g, 65 %). ^{1}H NMR (601 MHz, THF-d_8) δ 10.25 (s, 2H),

7.70 (dd, 4 H), 7.44 (dd, 4 H), 7.25 (t, 4 H), 7.15 (t, 2 H), 6.99-7.06 (m, 6 H), 6.52 (s, 2 H), 4.41 (d, 2 H), 3.75 (dd, 2 H), 3.60-3.63 (m, 8 H), 2.89 (d, 2 H), 2.75 (dt, 2 H), 2.25-2.30 (m, 2 H), 2.06 (s, 3 H), 1.76-1.79 (m, 8 H), 1.52-1.57 (m, 2 H), 1.25-1.29 (m, 2 H), 0.50-0.57 (m, 2 H). ^{13}C NMR (126 MHz, THF-d_8) δ 166.32, 150.09, 148.67, 130.03, 128.70, 128.20, 128.16, 127.68, 127.14, 126.53, 125.85, 116.00, 69.99, 68.03, 60.42, 54.97, 30.67, 26.20, 23.32, 20.59.

Elemental analysis: calculated: C, 70.20; H, 7.70; N, 5.01 %. Found: C, 70.10; H, 7.81; N, 4.96 %.

m/z (APPI-MS): 677.3 $[LH_2K + H]^+$ (calc: 677.3).

Synthesis and characterisation of complex 3

Complex **1** (500 mg, 0.62 mmol) was weighed into a Schlenk flask and dissolved in dry THF (7.5 mL) in the glove box. $ZnEt_2$ (154 mg, 1.24 mmol) was added dropwise into a vial with dry THF (2.5 mL) in the glove box and the resulting solution was added dropwise to the solution of complex **1**. The reaction mixture was stirred for 1 h at ambient temperature under an argon atmosphere in the glove box. THF was subsequently removed *in vacuo*, resulting in a pale yellow powder (0.39 g, 64 %). ^{1}H NMR (601 MHz, THF-d_8) δ 7.82 (dd, 4 H), 7.65 (dd, 4 H), 7.23 (t, 4 H), 7.09 (t, 4 H), 7.02 (t, 2 H), 6.95 (t, 2 H), 6.61 (s, 2 H), 4.22 (d, 2 H), 4.07 (t, 2 H), 3.61-3.63 (m, 8 H), 2.86 (dt, 2 H), 2.62 (d, 2 H), 2.52 (q, 2 H), 2.07 (s, 3 H), 1.76-1.79 (m, 8 H), 1.49-1.66 (m, 8 H), 0.55 (t, 6 H), -0.46- -0.30 (ddq, 4 H). ^{13}C NMR (126 MHz, THF-d_8) δ 161.55, 155.25, 154.44, 130.32, 128.25, 128.17, 127.35, 127.16, 126.98, 125.54, 125.22, 73.27, 68.03, 67.74, 59.24, 56.13, 29.62, 26.19, 25.62, 20.51, 13.36, -0.54.

Elemental analysis: calculated: C, 66.60; H, 7.01; N, 2.82 %. Found: C, 66.51; H, 6.91; N, 2.88 %.

m/z (APPI-MS): 793.3 $[LZn_2Et + H]^+$, 815.2 $[LZn_2Et + Na]^+$ (calc: 845.2).

Synthesis and characterisation of complex 4

Complex **2** (500 mg, 0.61 mmol) was weighed into a Schlenk flask and dissolved in dry THF (7.5 mL) in the glove box. $ZnEt_2$ (151 mg, 1.22 mmol) was added dropwise into a vial with dry THF (2.5 mL) in the glove box and the resulting solution was added dropwise to the solution of complex **2**. The reaction mixture was stirred for 1 h at ambient temperature under an argon atmosphere in the glove box. THF was subsequently removed *in vacuo*, resulting in a dark yellow powder (0.37 g, 60 %). ^{1}H NMR (601 MHz, THF-d_8) δ 7.77 (dd, 4 H), 7.61 (dd, 4 H), 7.22 (t, 4 H), 7.08 (t, 4 H), 7.01 (t, 2 H), 6.93 (t, 2 H), 6.64 (s, 2 H), 4.30 (d, 2 H), 4.03 (dd, 2 H), 3.61-3.63 (m, 8 H), 2.93 (dt, 2 H), 2.62 (d, 2 H), 2.52 (q, 2 H), 2.08 (s, 3 H), 1.76-1.78 (m, 8 H), 1.46-1.58 (m, 6 H), 1.33-1.38 (m, 2 H), 0.58 (t, 6 H), -0.52- -0.38 (ddq, 4 H). ^{13}C NMR (126 MHz, THF-d_8) δ 161.15, 155.90, 154.70, 130.67, 128.15, 127.35, 126.88, 126.75, 125.46, 125.03, 71.66, 68.03, 58.26, 56.23, 29.99, 26.19, 20.47, 20.16, 13.31, -1.01.

Elemental analysis: calculated: C, 65.53; H, 6.90; N, 2.78 %. Found: C, 65.35; H, 6.65; N, 2.77 %.

m/z (APPI-MS): 793.3 $[LZn_2Et + H]^+$, 831.2 $[LZn_2Et + K]^+$ (calc: 861.2).

Synthesis and characterisation of [BnONa]

BnOH (56 µL, 0.56 mmol) was dissolved in dry THF (8 mL) in a Schlenk flask. NaH (14.7 mg, 0.61 mmol) was slowly added into the BnOH solution. The resulting mixture was stirred over the weekend at ambient temperature under an argon atmosphere in the glove box with the Schlenk tap open to allow H_2 gas evolution. THF was subsequently removed *in vacuo* resulting in an off-white sticky solid, which was used directly in preparation of a stock solution for polymerisation studies. Both ^{1}H and ^{13}C NMR spectra show a major and minor set of product resonances. DOSY NMR analysis suggests that these two species are both BnONa products but with different aggregation states (refer to Fig. S13-14). ^{1}H NMR (601 MHz, THF-d_8) for the major product, **$[BnONa]_3$**, δ 7.36 (d, 2 H), 7.19 (t, 2 H), 7.04 (t, 1 H), 4.97 (s, 2 H). ^{13}C NMR (126 MHz, THF-d_8) δ 128.25, 126.24, 125.55.

Synthesis and characterisation of [BnOK]

BnOH (56 µL, 0.56 mmol) was dissolved in dry THF (8 mL) in a Schlenk flask. KH (24.5 mg, 0.61 mmol) was slowly added into the BnOH solution. The resulting mixture was stirred over the weekend at ambient temperature under an argon atmosphere in the glove box with the Schlenk tap open to allow H_2 gas evolution. THF was subsequently removed *in vacuo* resulting in a white sticky solid, which was used directly in preparation of a stock solution for polymerisation studies. ^{1}H NMR (500 MHz, THF-d_8) δ 7.34 (d, 2 H), 7.16 (t, 2 H), 7.00 (t, 1 H), 4.93 (s, 2 H). ^{13}C NMR (126 MHz, THF-d_8) δ 150.88, 128.30, 126.72, 125.54.

General experimental procedure for the ring-opening polymerisation of *rac*-LA in toluene

In the glove box, in two separate air-tight vials with magnetic stirrer bars, *rac*-LA (100 eq., 200 mg, 1.39 mmol) was dissolved in dry toluene (1 mL) and complex **3** or **4** (13.9 µmol) was dissolved in dry toluene (0.39 mL). To aid solubility, both solutions were stirred for 3 minutes at 60 °C using DrySyn heating blocks before the catalyst solution was added to the LA solution. The polymerisation was initiated by addition of BnOH (2.88 µL, 27.8 µmol). The reaction was subsequently stirred at the appropriate temperature for the required time. Upon completion, the reaction was quenched in excess hexane. The volatiles were removed under compressed air and an aliquot was dissolved in $CDCl_3$ for NMR spectroscopic analysis.

General experimental procedure for the ring-opening polymerisation of LA in THF

In the glove box, in an air-tight vial with a magnetic stirrer bar, *rac*-LA or L-LA (100 eq., 200 mg, 1.39 mmol) and complex **1**-**4** or **[BnOM]** (13.9 µmol, M = Na or K) were dissolved in dry THF (1.39 mL). The polymerisation was initiated by addition of BnOH (2.88 µL, 27.8 µmol). The reaction was subsequently stirred using DrySyn heating blocks at the appropriate temperature for the required time. Upon completion, the reaction was quenched in excess hexane. The volatiles were removed under compressed air and an aliquot was dissolved in $CDCl_3$ for NMR spectroscopic analysis.

General experimental procedure for the ring-opening polymerisation of ε-CL

In the glove box, in an air-tight vial with a magnetic stirrer bar, ε-CL (100 eq., 154 µL, 1.39 mmol) and complex **3** or **4** (13.9 µmol) were dissolved in dry THF (1.39 mL). The polymerisation was initiated by addition of BnOH (2.88 µL, 27.8 µmol). The reaction was subsequently stirred using DrySyn heating blocks at the appropriate temperature for the required time. Upon completion, the reaction was quenched in excess hexane. The volatiles were removed under compressed air and an aliquot was dissolved in $CDCl_3$ for NMR spectroscopic analysis.

General experimental procedure for the ring-opening polymerisation of δ-VL

In the glove box, in an air-tight vial with a magnetic stirrer bar, δ-VL (100 eq., 129 µL, 1.39 mmol) and complex **3** or **4** (13.9 µmol) were dissolved in dry THF (1.39 mL). The polymerisation was initiated by addition of BnOH (2.88 µL, 27.8 µmol). The reaction was subsequently stirred using DrySyn heating blocks at the appropriate temperature for the required time. Upon completion, the reaction was quenched in excess hexane. The volatiles were removed under compressed air and an aliquot was dissolved in $CDCl_3$ for NMR spectroscopic analysis.

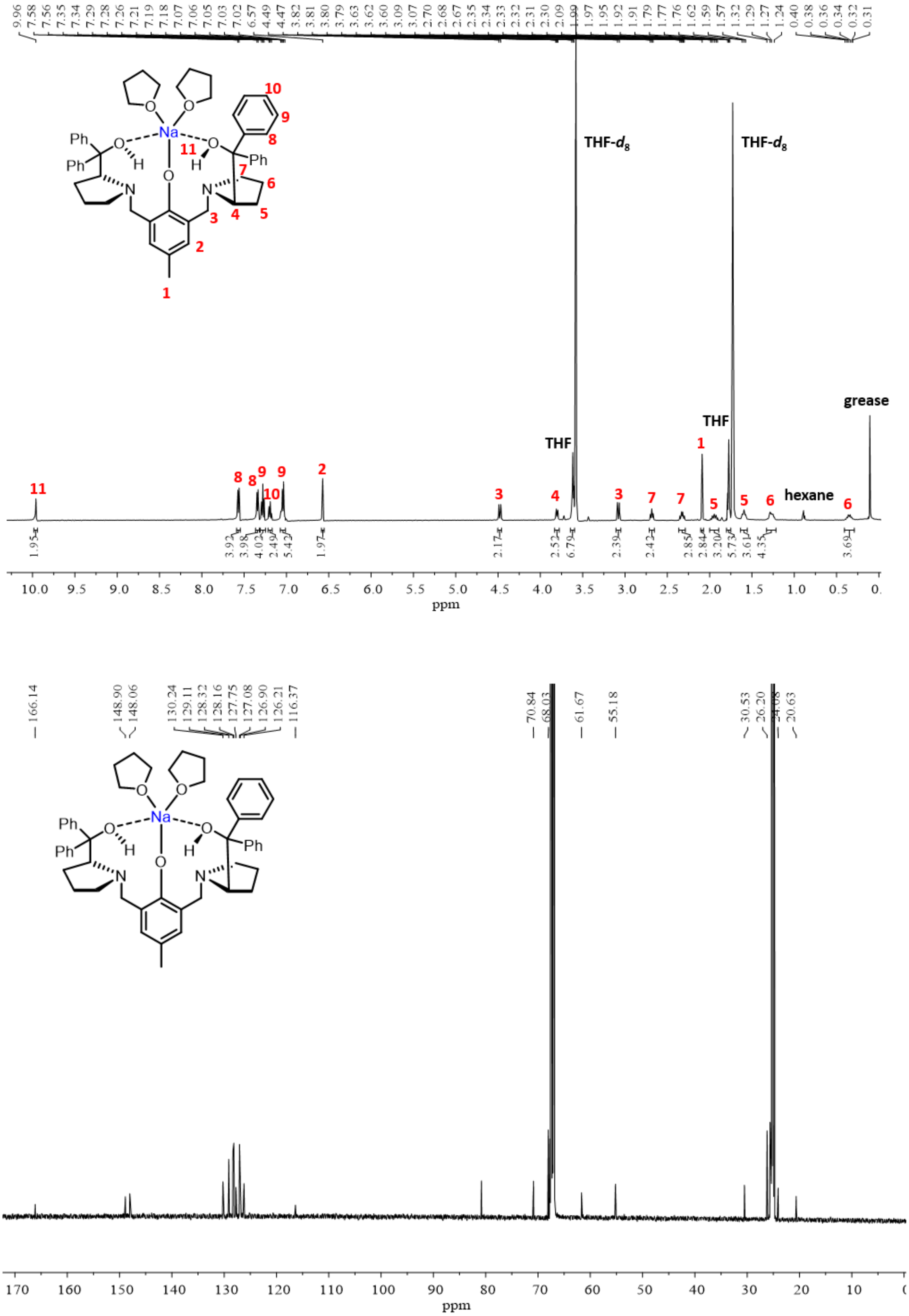

Figure S1. ^{1}H and ^{13}C NMR spectra of complex **1** in THF-d_8 (298 K).

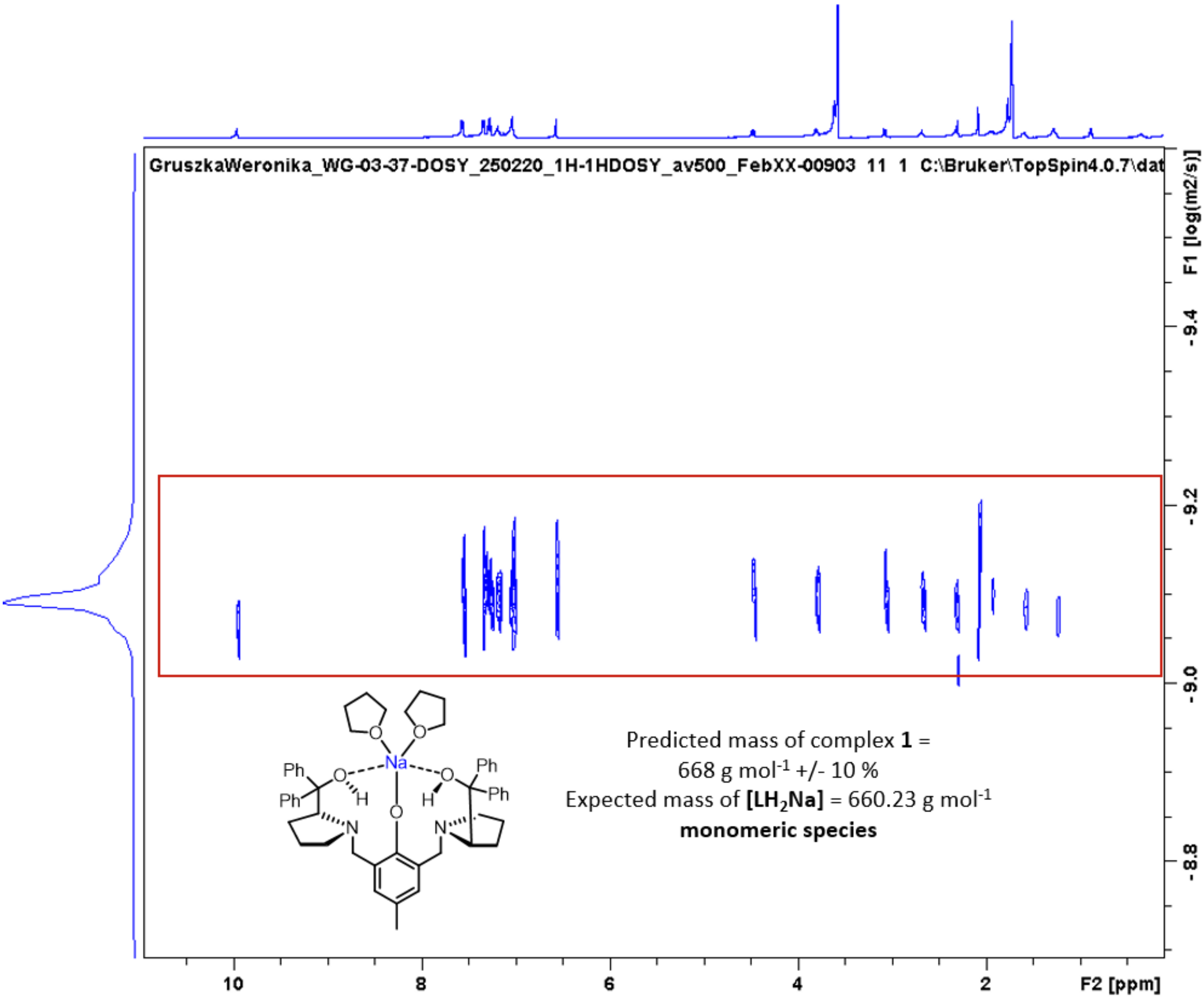

Figure S2. DOSY NMR spectrum of complex **1** in THF-d_8 (298 K).

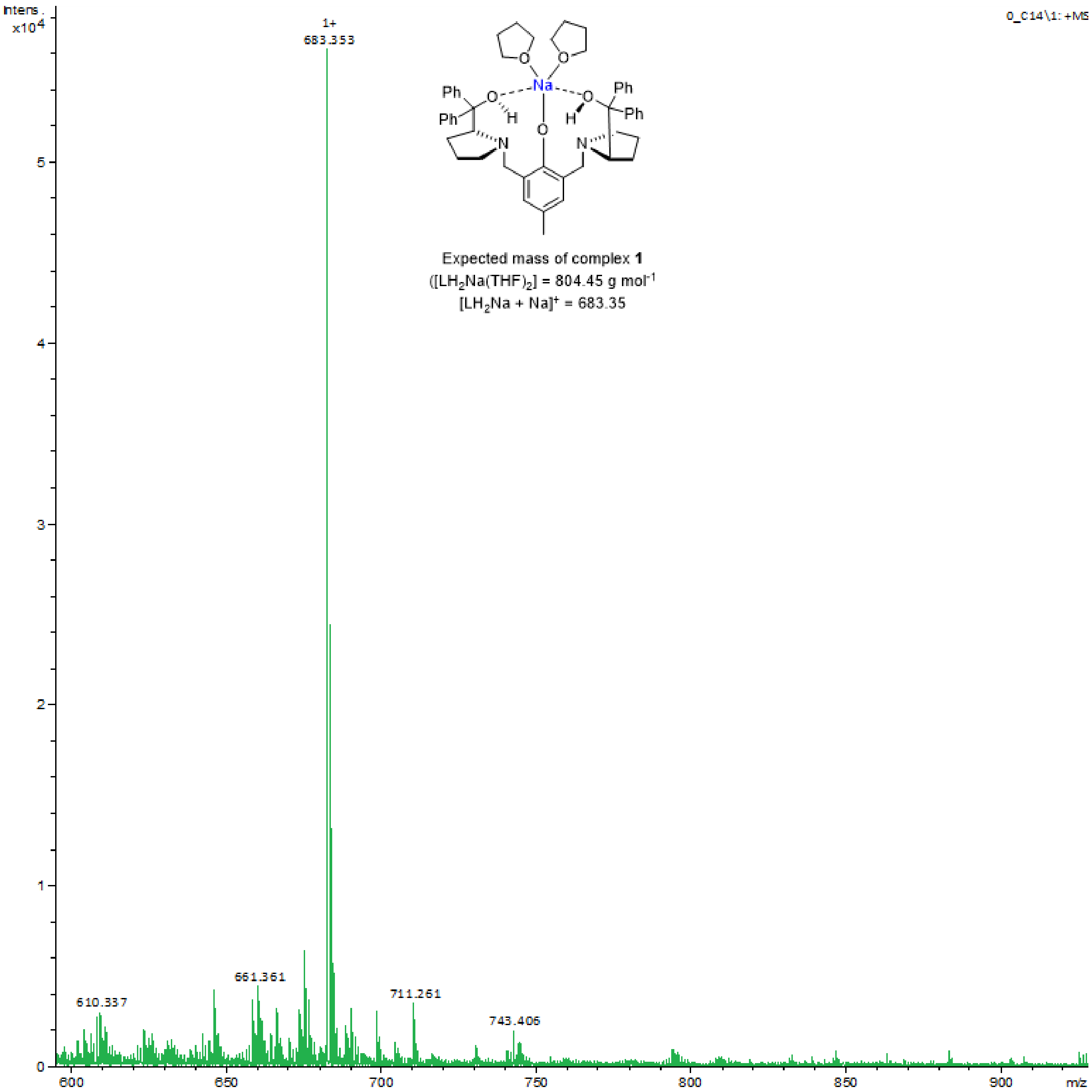

Figure S3. MALDI-ToF spectrum of complex **1**.

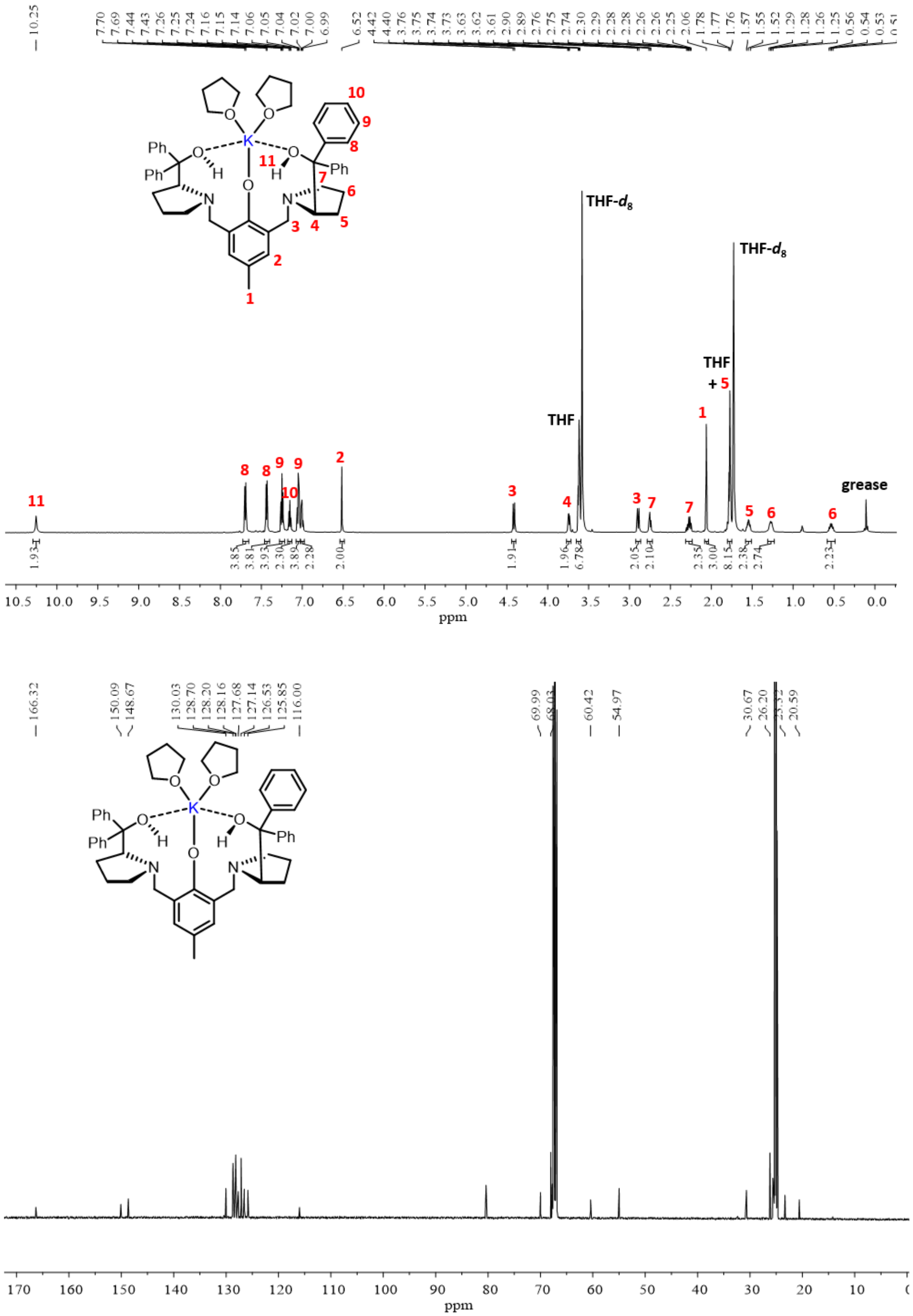

Figure S4. ^{1}H and ^{13}C NMR spectra of complex **2** in THF-d_8 (298 K).

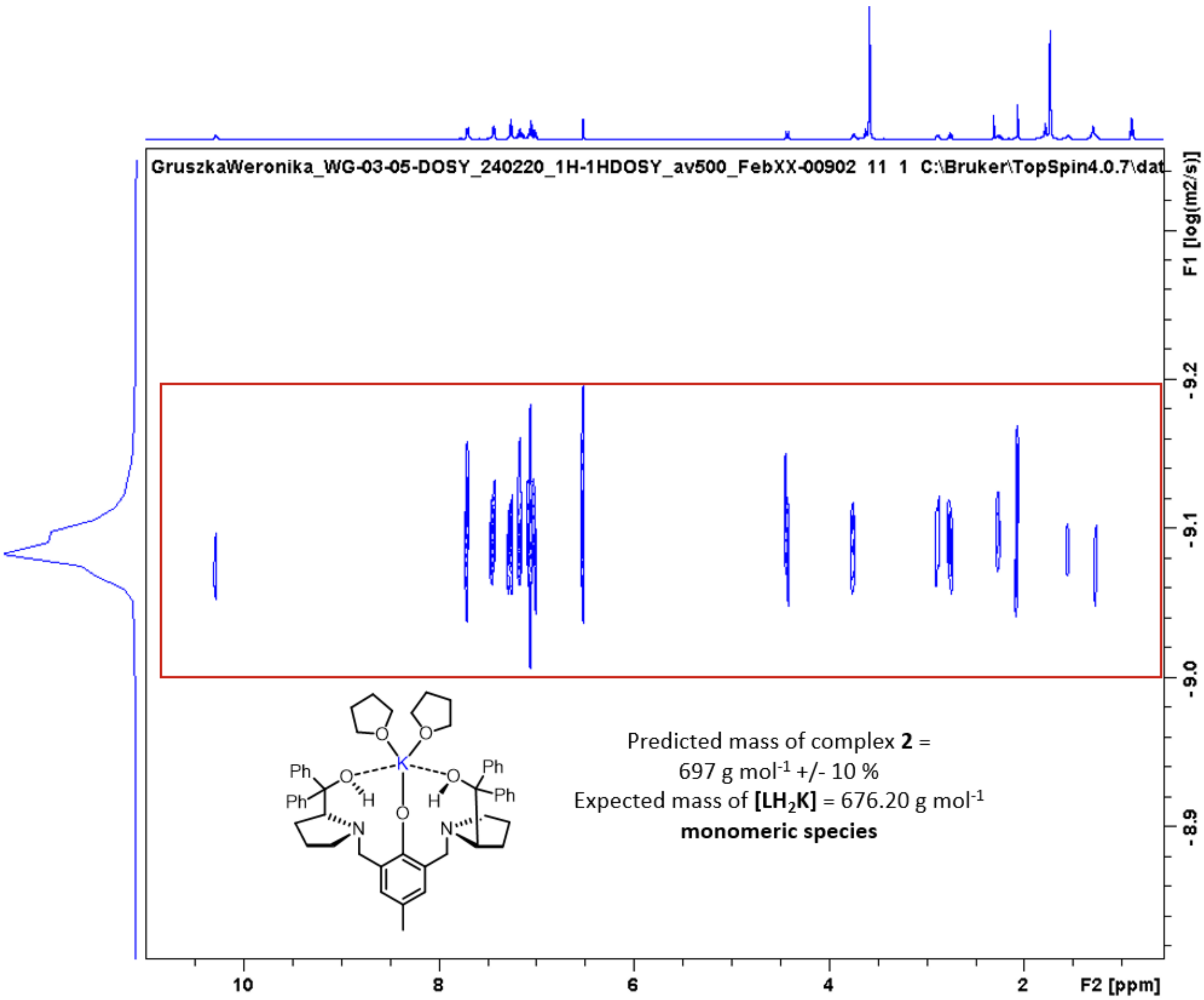

Figure S5. DOSY NMR spectrum of complex **2** in THF-d_8 (298 K).

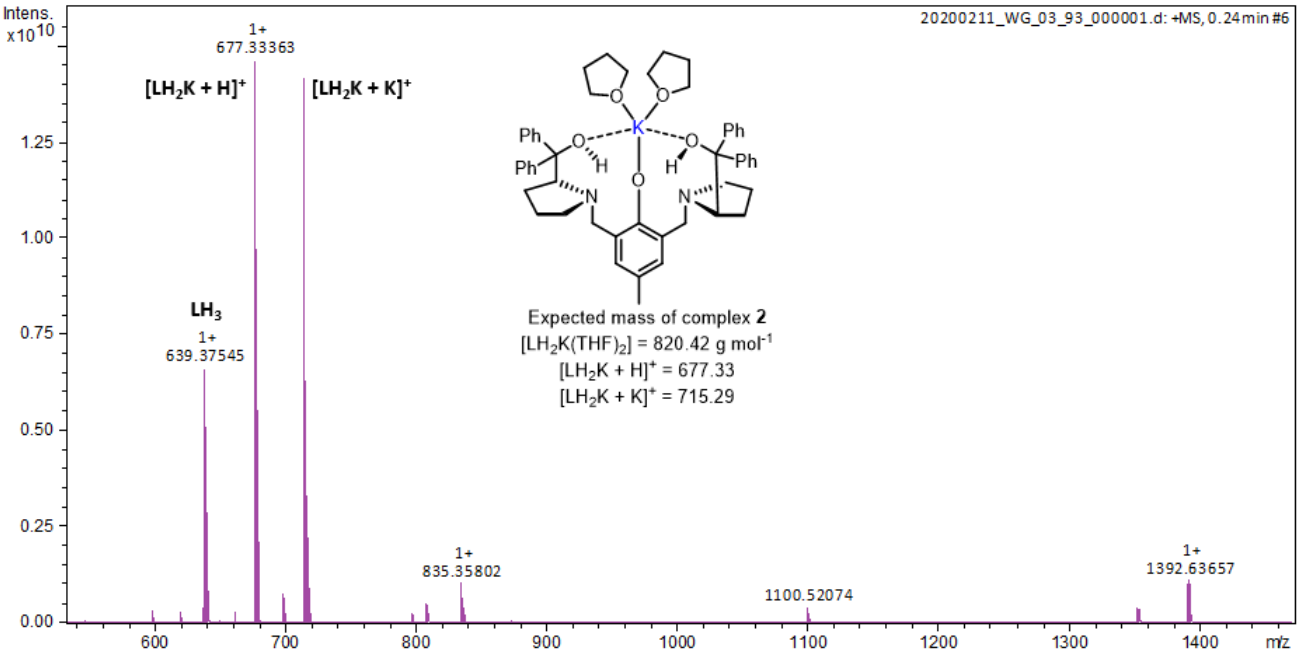

Figure S6. APPI-MS spectrum of complex 2.

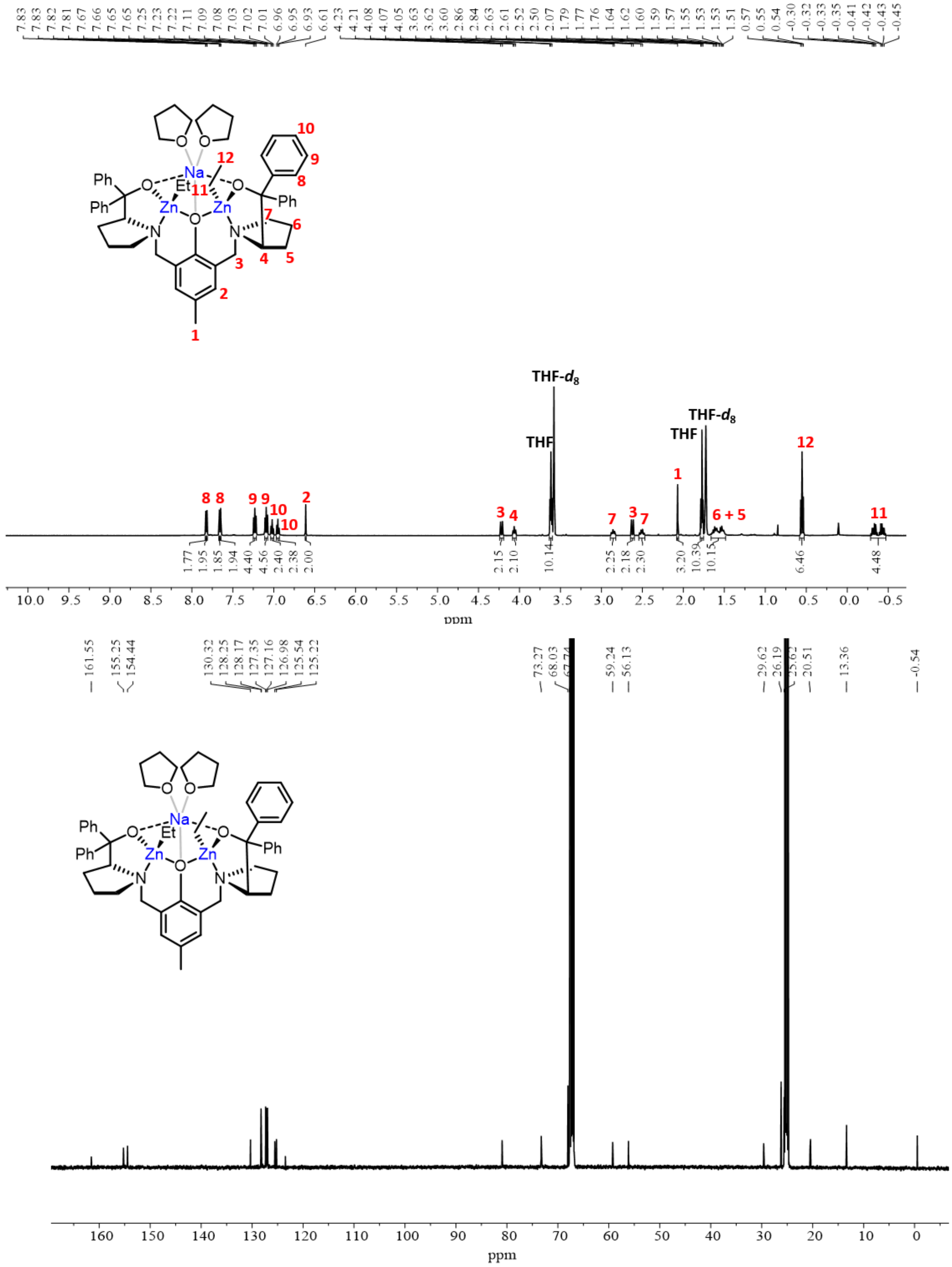

Figure S7. ^{1}H and ^{13}C NMR spectra of complex **3** in THF-d_8 (298 K).

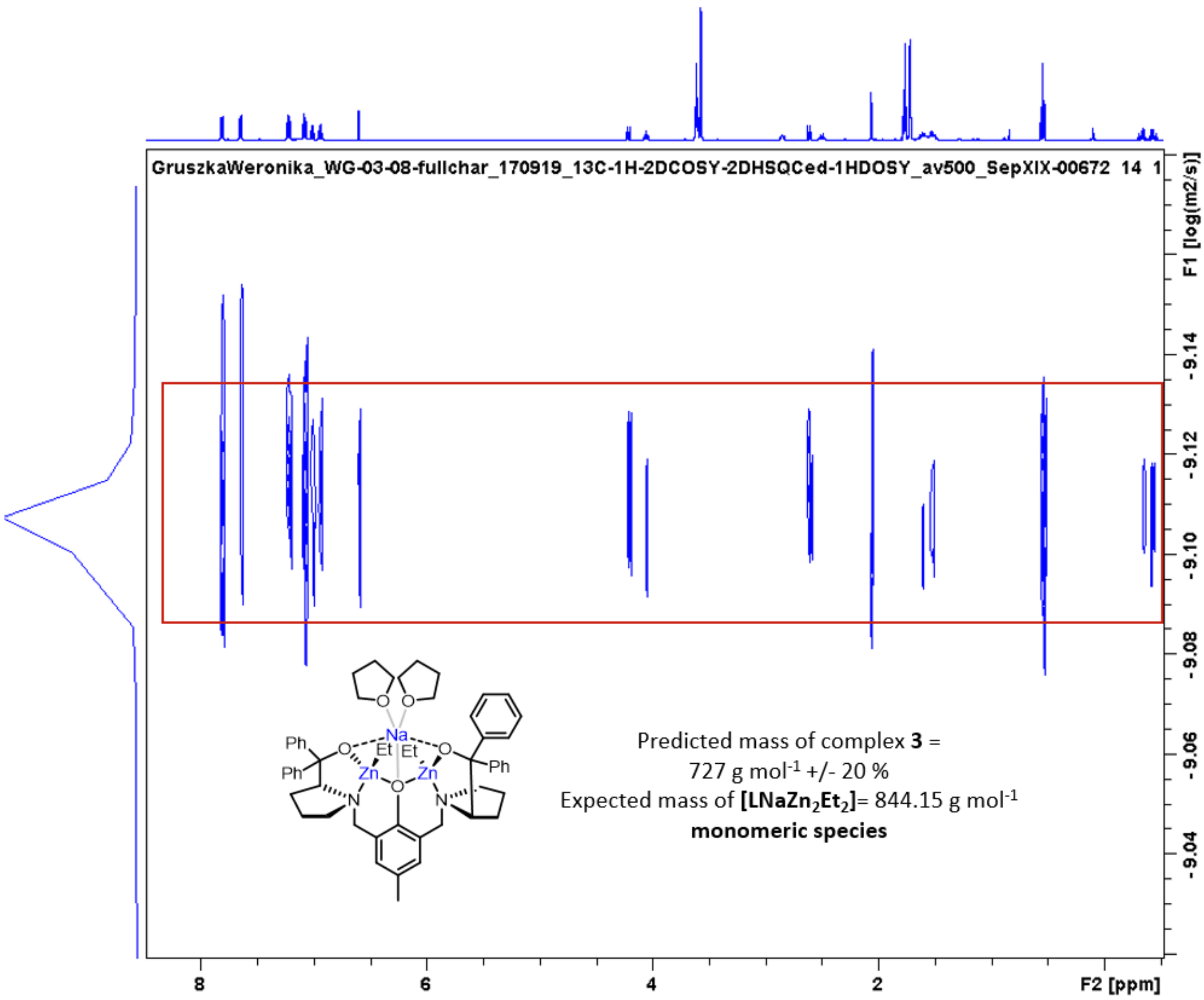

Figure S8. DOSY NMR spectrum of complex **3** in THF-d_8 (298 K).

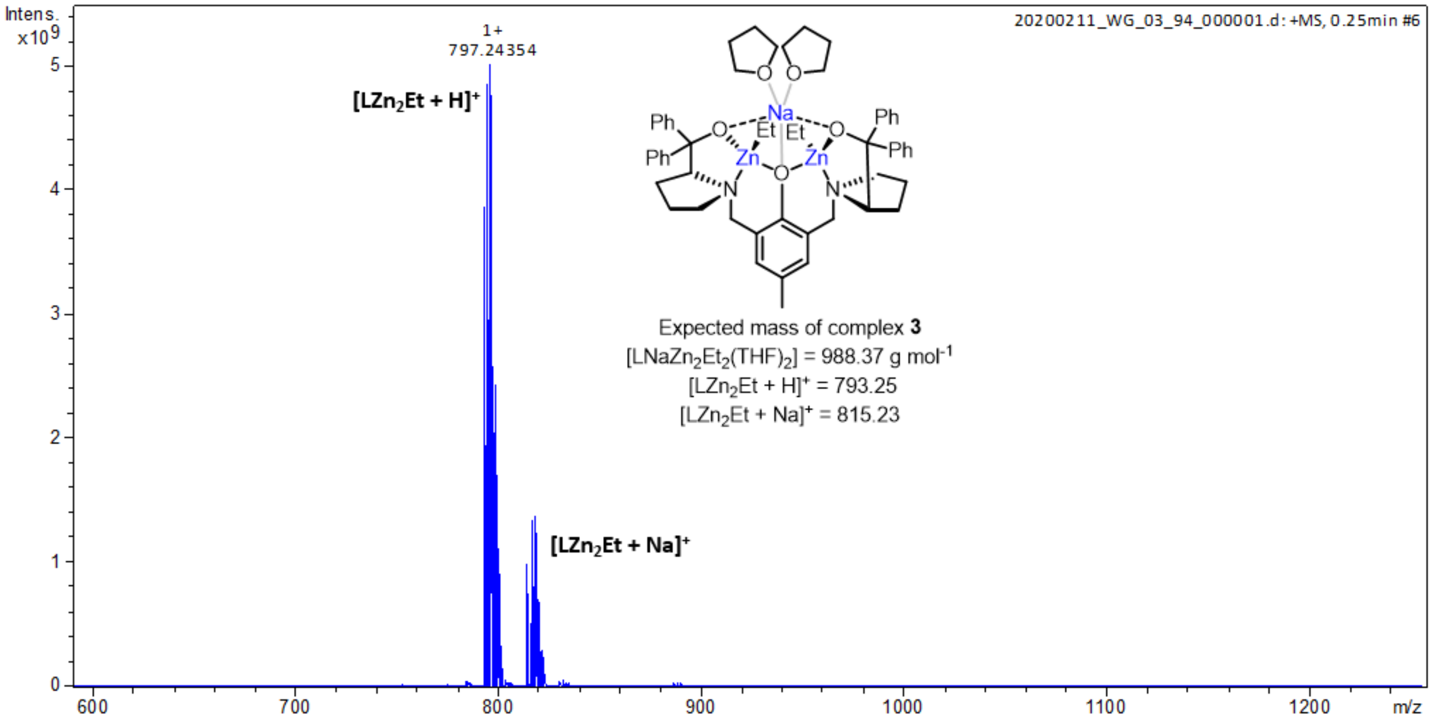

Figure S9. APPI-MS spectrum of complex **3**. Degradation of complex **3** to form [LZn_2Et + H^+] and [LZn_2Et + Na^+] was attributed to performing the APPI-MS measurement under non air-/moisture-sensitive conditions.

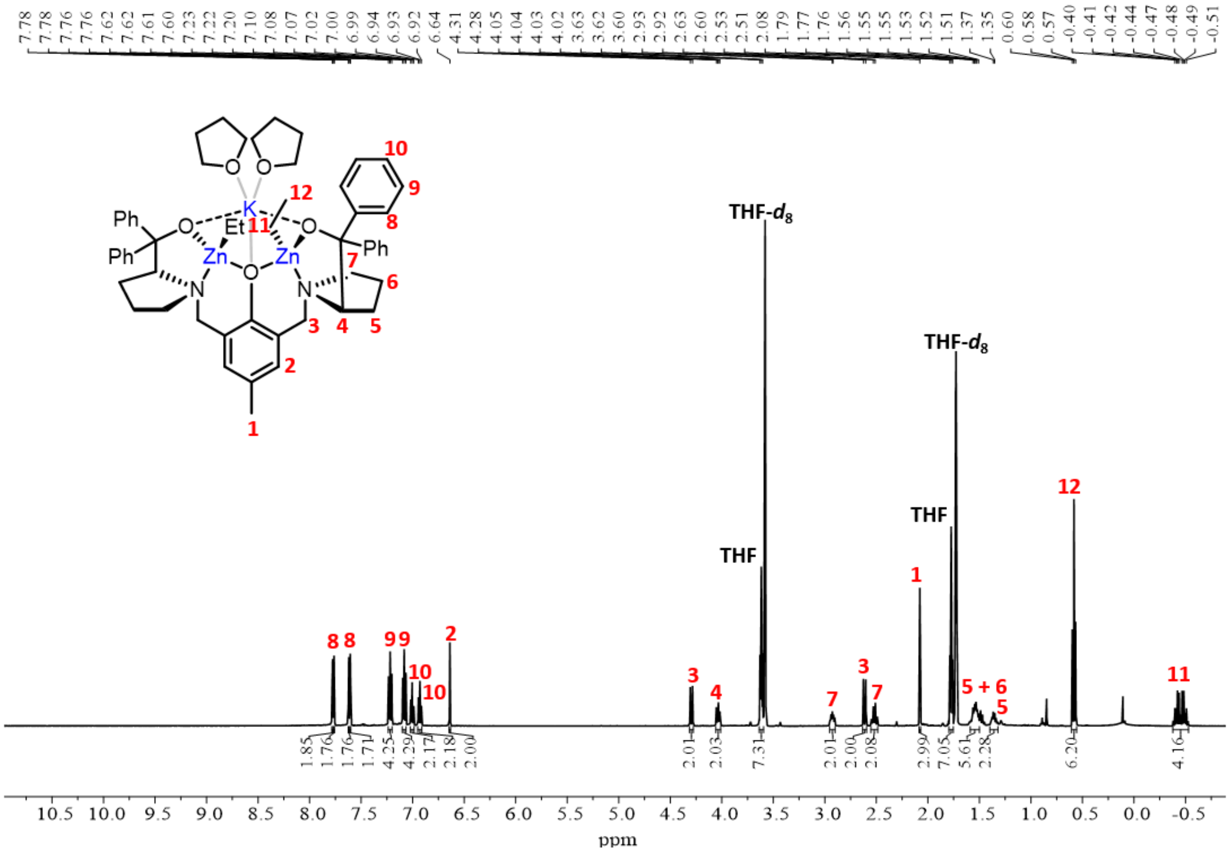

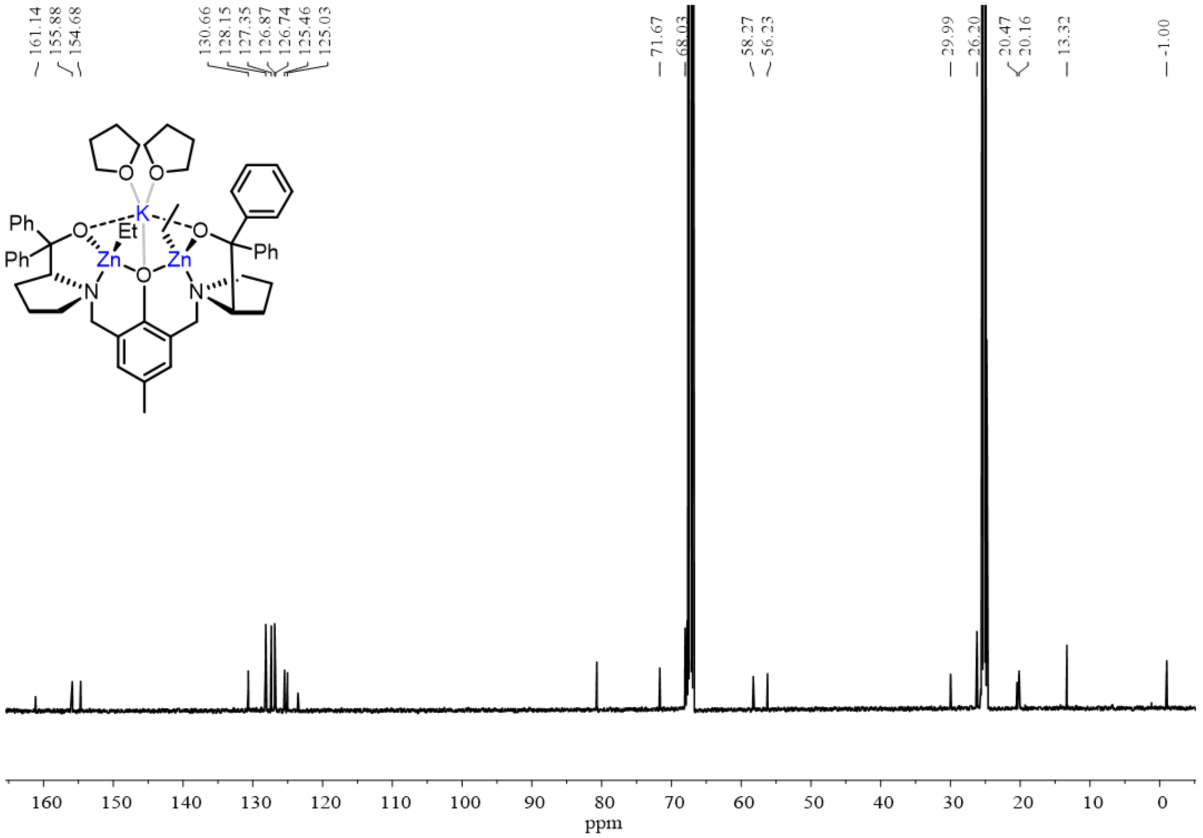

Figure S10. ^1H and ^{13}C NMR spectra of complex **4** in THF-d_8 (298 K).

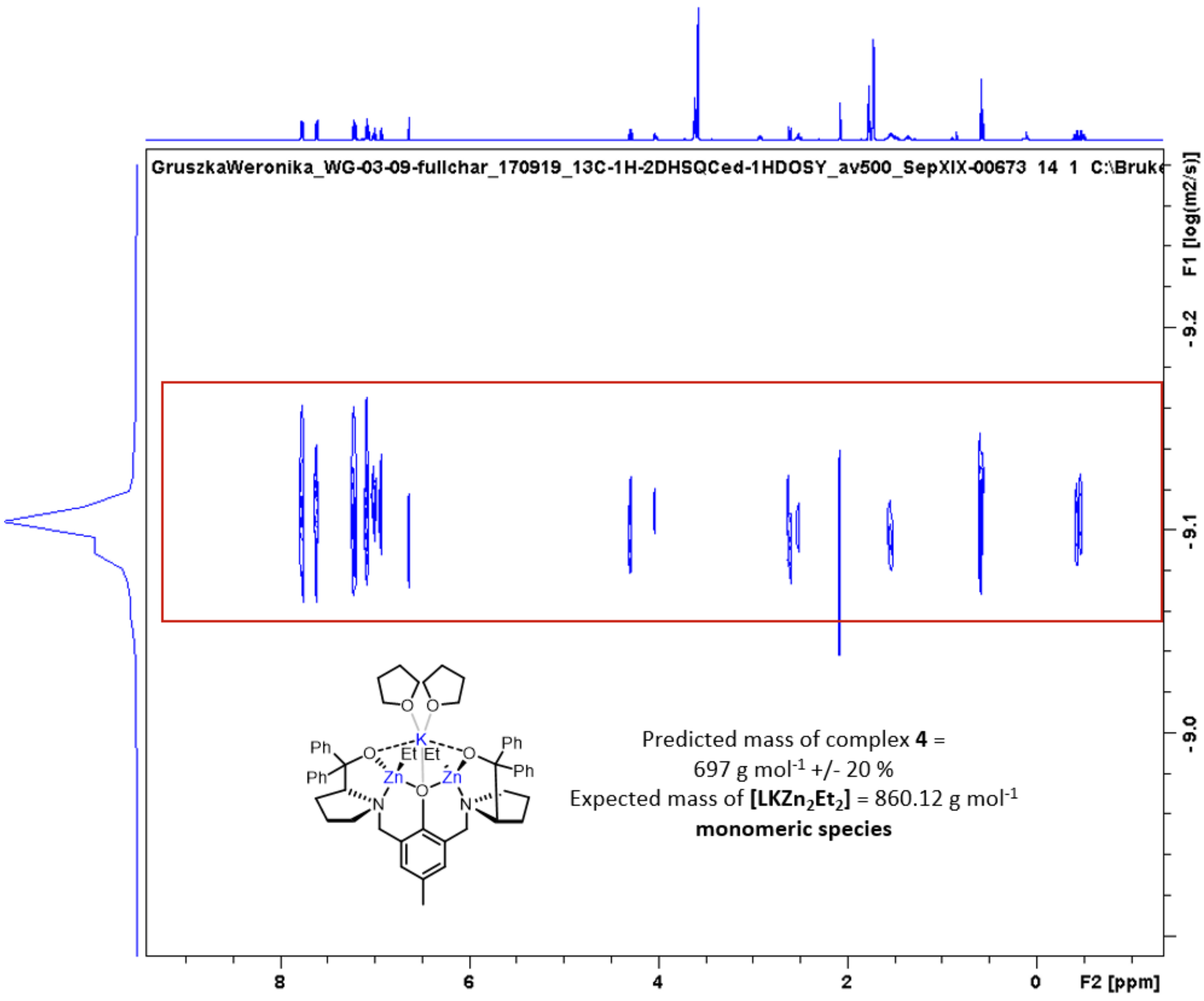

Figure S11. DOSY NMR spectrum of complex **4** in THF-d_8 (298 K).

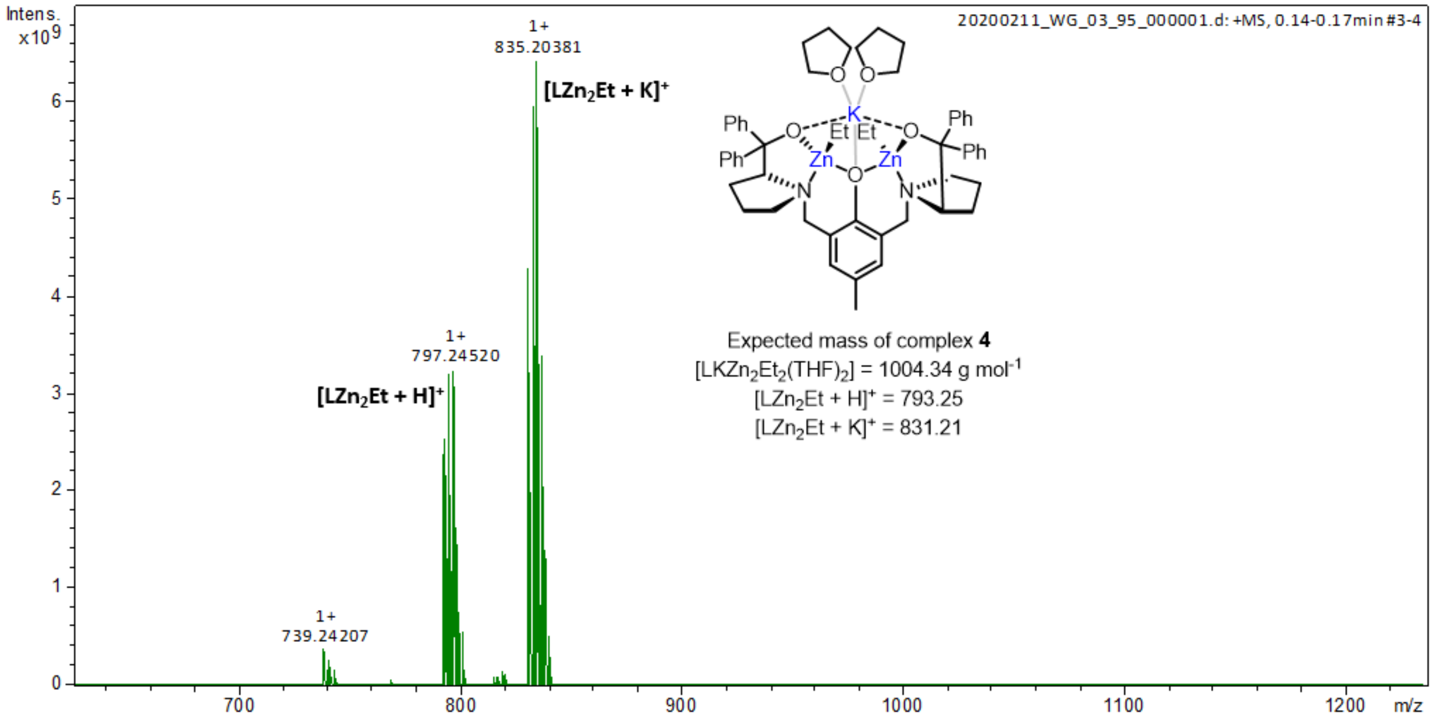

Figure S12. APPI-MS spectrum of complex **4**. Degradation of complex **4** to form [$LZn_2Et + H^+$] and [$LZn_2Et + K^+$] was attributed to performing the APPI-MS measurement under non air-/moisture-sensitive conditions.

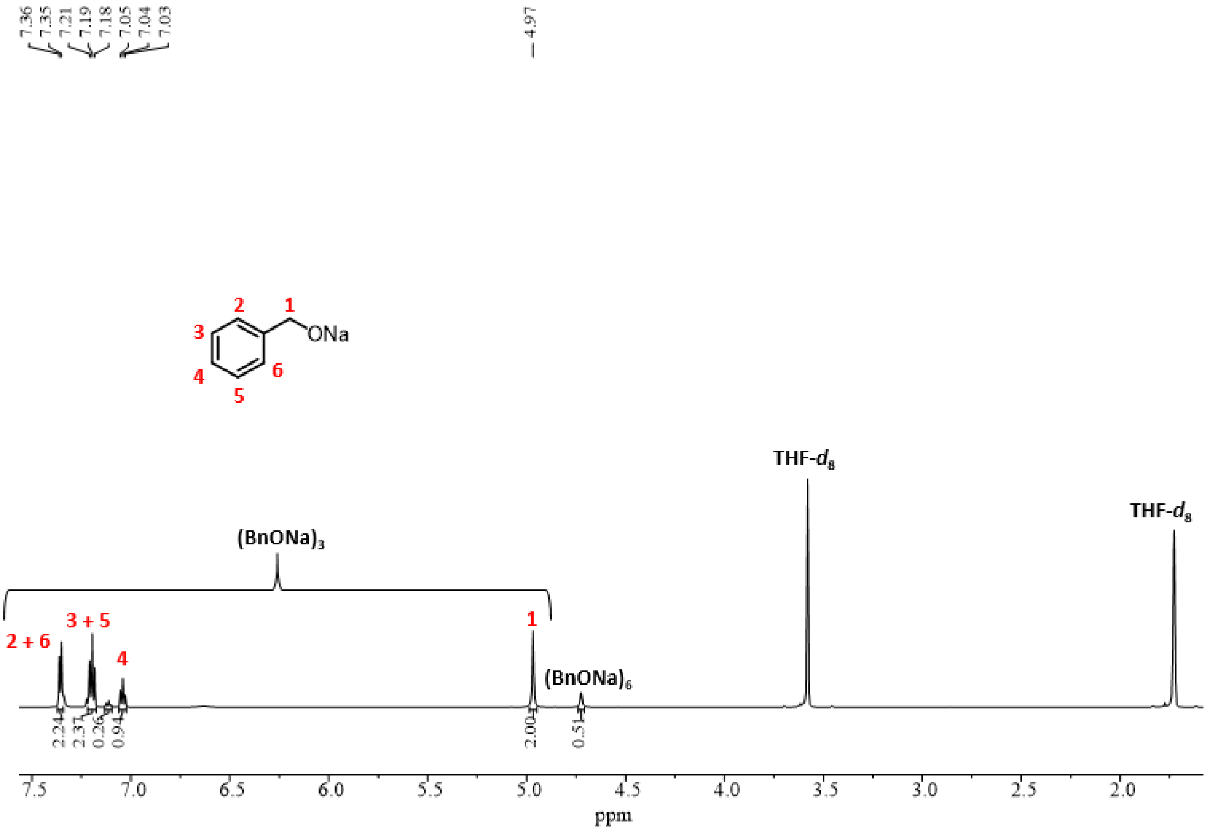

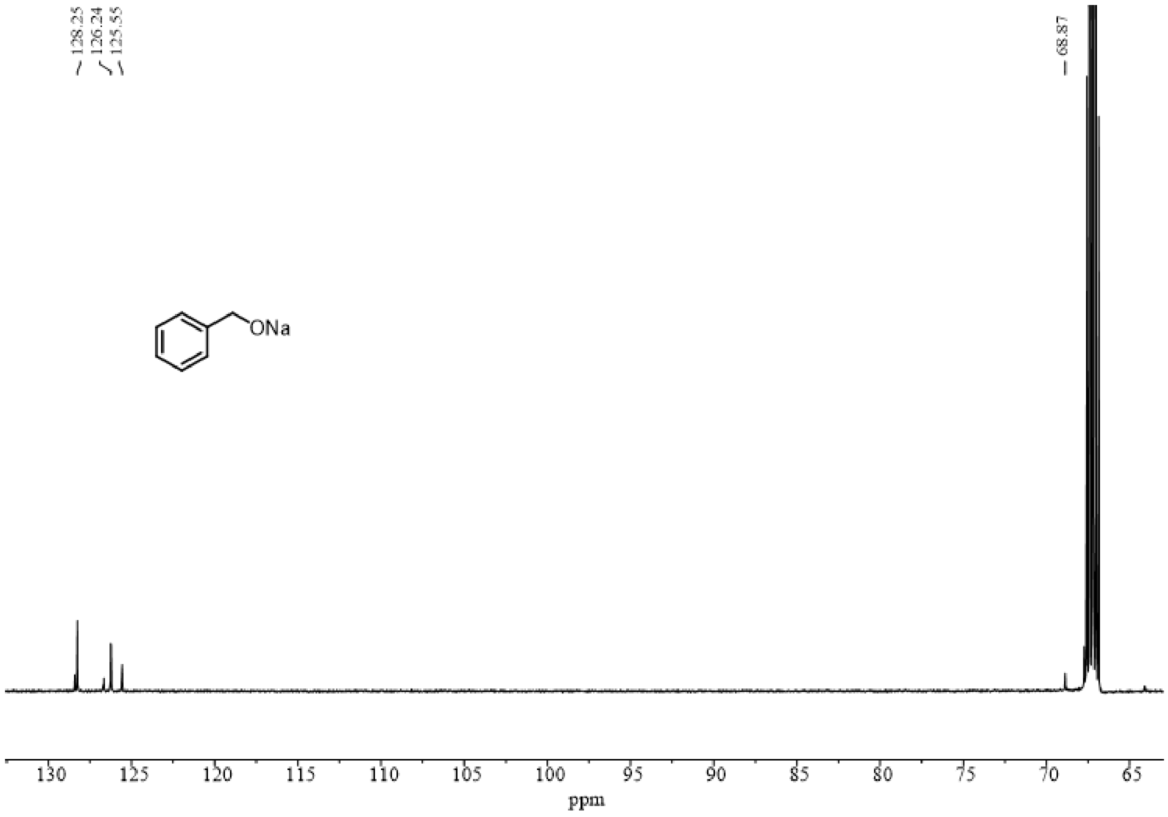

Figure S13. ^{1}H and ^{13}C NMR spectra of **[BnONa]** in THF-d_8 (298 K). Trimeric and hexameric nature of **[BnONa]** was assigned *via* DOSY NMR (Figure S14).

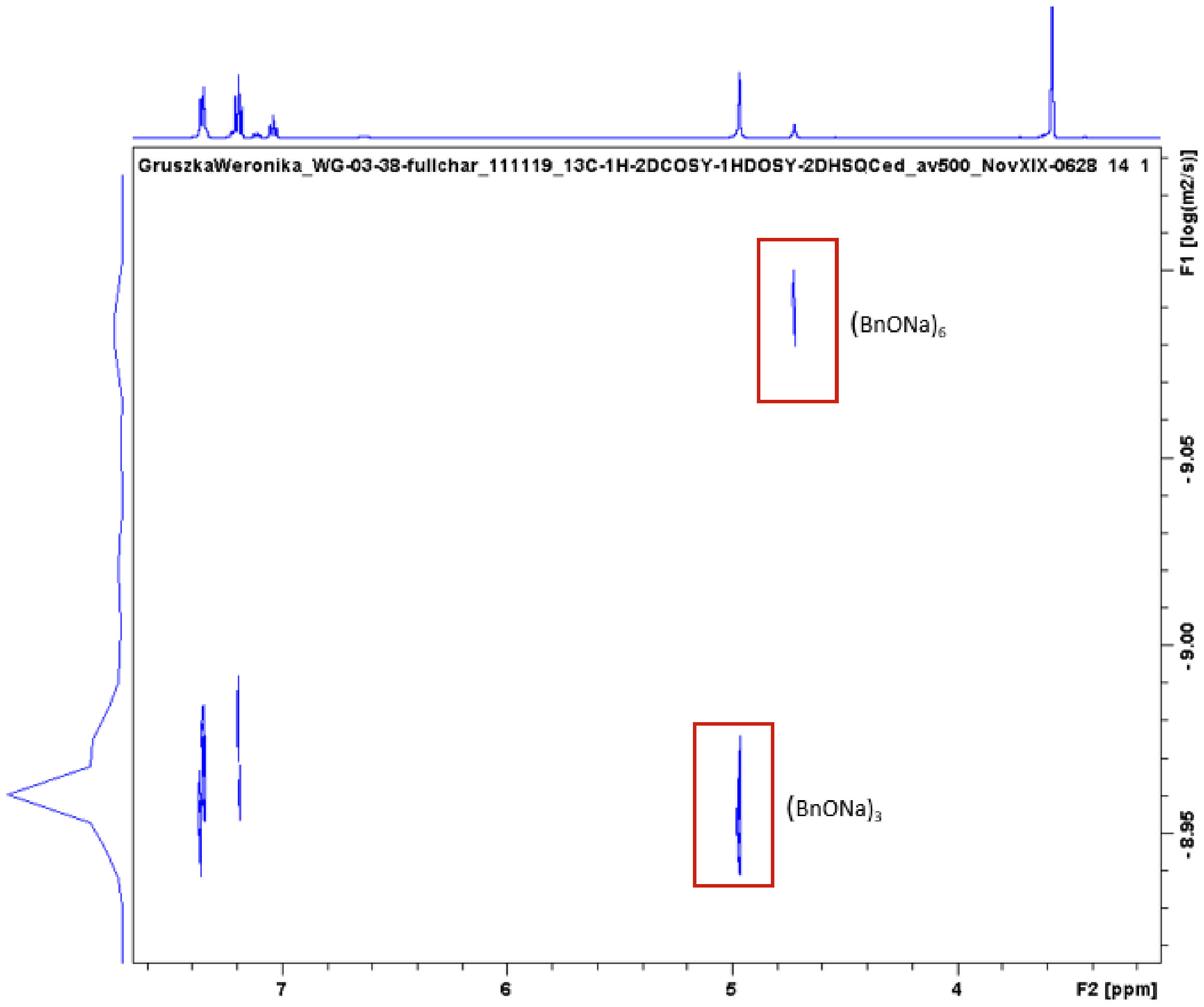

Figure S14. DOSY NMR spectrum of **[BnONa]** in THF-d_8 (298 K).

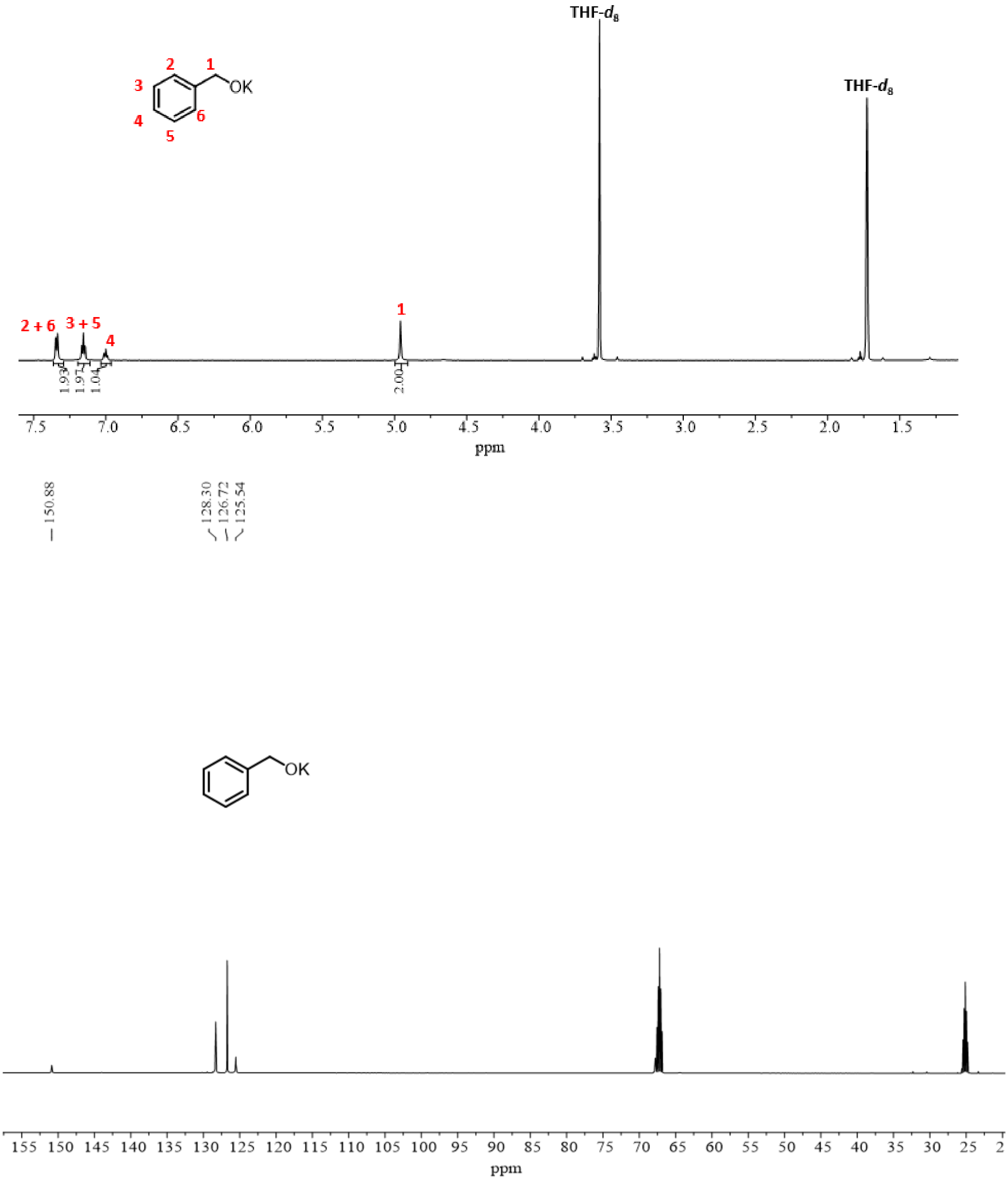

Figure S15. ^{1}H and ^{13}C NMR spectra of **[BnOK]** in THF-d_8 (298 K). The aggregation state of **[BnOK]** was investigated by DOSY NMR (Figure S16).

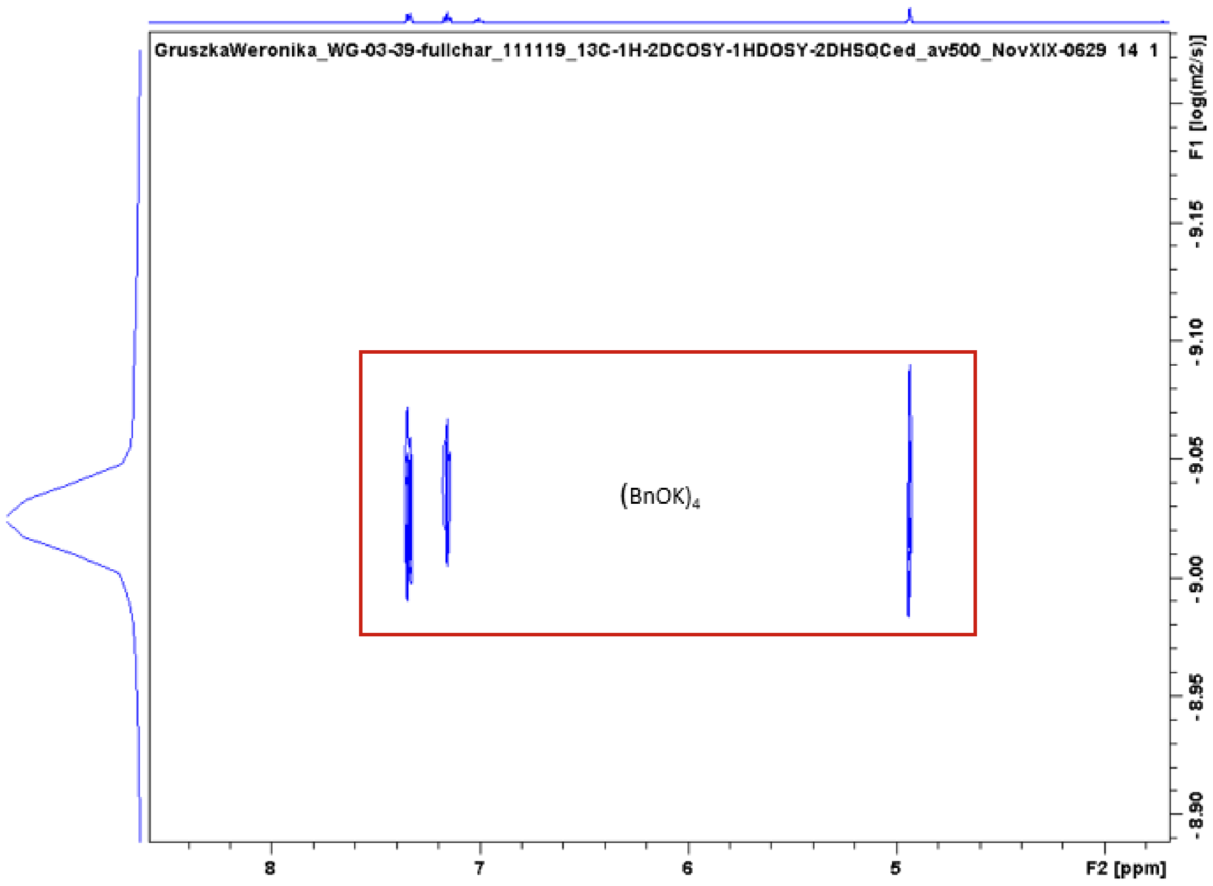

Figure S16. DOSY NMR spectrum of [BnOK] in THF-d_8 (298 K).

Table S1. Full table of results for the ROP of *rac*-LA catalysed by complexes **3** and **4** in the presence of 2 eq. of BnOH in THF at R.T.

Entry	Cat.	LA:cat:BnOH	Time (min)	Conv.[a] (%)	$M_{n,obs}$[b] (Da)	$M_{n,calc}$[c] (Da)	*Đ*[b]	P_i[d]
**1**	**3**	100:1:0	2.5	12	-	-	-	-
**2**	**3**	100:1:2	0.33	47	2100	3400	1.27	-
**3**	**3**	100:1:2	1.25	51	2600	3700	1.15	0.49
**4**	**3**	100:1:2	2.5	71	2300	5100	1.23	-
**5**	**3**	100:1:2	5	79	3400	5700	1.39	0.53
**6**	**3**	100:1:2	7.5	86	3900	6200	1.38	-
**7**	**3**	100:1:2	10	87	5000	6300	1.41	0.50
**8**	**3**	200:1:2	15	74	6100	10700	1.28	-
**9**	**3**	300:1:2	22.5	69	9100	15000	1.47	-
**10**	**3**	400:1:2	30	61	9200	17600	1.29	-
**11**	**3**	500:1:2	37.5	7	-	-	-	-
**12**	**3**	100:1:1	5	15	-	-	-	-
**13**	**3**	100:1:10	1.25	86	1100	1200[e]	1.15	-
**14**	**4**	100:1:0	2.5	13	-	-	-	-
**15**	**4**	100:1:2	0.08	45	1900	3300	1.74	-
**16**	**4**	100:1:2	0.167	53	2400	3800	1.80	0.62
**17**	**4**	100:1:2	0.33	60	2500	4300	1.36	0.60
**18**	**4**	100:1:2	0.67	67	2900	4800	1.45	0.49
**19**	**4**	100:1:2	1.25	81	3900	5800	1.42	0.53
**20**	**4**	100:1:2	2	93	4300	6700	1.40	-
**21**	**4**	200:1:2	4	84	9200	12100	1.48	-
**22**	**4**	300:1:2	6	73	12100	15800	1.39	-
**23**	**4**	400:1:2	8	48	9900	13800	1.30	-
**24**	**4**	500:1:2	10	51	11200	18400	1.32	-
**25**	**4**	100:1:1	1.25	20	-	-	-	-
**26**	**4**	100:1:10	0.67	94	1200	1300[e]	1.32	-

[LA] = 1 M in THF. [a] Conversion calculated using ^{1}H NMR spectroscopy.[b] $M_{n,obs}$ and Đ determined by gel permeation chromatography using polystyrene standards in THF. Values corrected by Mark-Houwink factor (0.58).[9] [c] $M_{n,calc}$ of polymers calculated from the monomer conversion $M_{n,calc}$ = M_0 x ([M]/[I]) x conversion assuming 2 chains per catalyst. [d] Determined by homodecoupled ^{1}H NMR spectroscopy. [e] $M_{n,calc}$ calculated from the monomer conversion $M_{n,calc}$ = M_0 x ([M]/[I]) x conversion assuming 10 chains per catalyst.

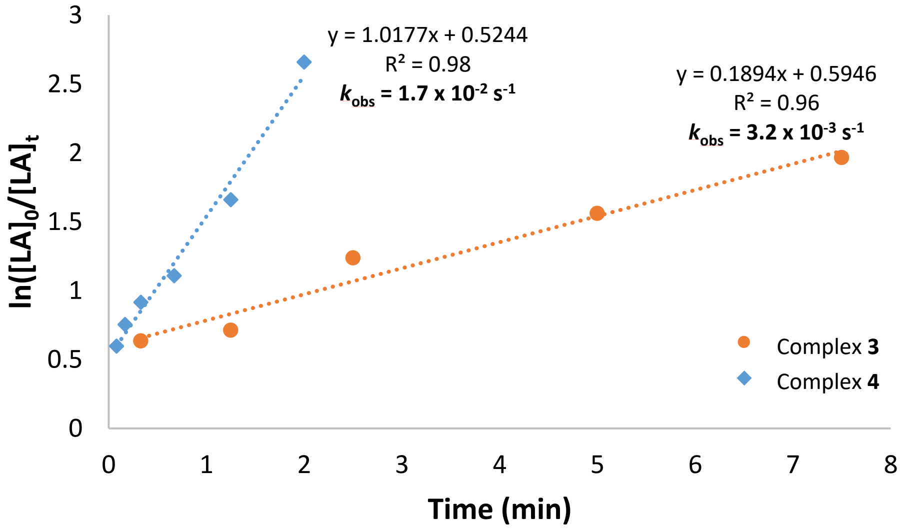

Figure S17. Plots of ln([LA]$_0$/[LA]$_t$) *vs*. time (min) for ROP of *rac*-LA with complexes **3** and **4** ([LA] = 1 M in THF, R.T., 2 eq. BnOH used).

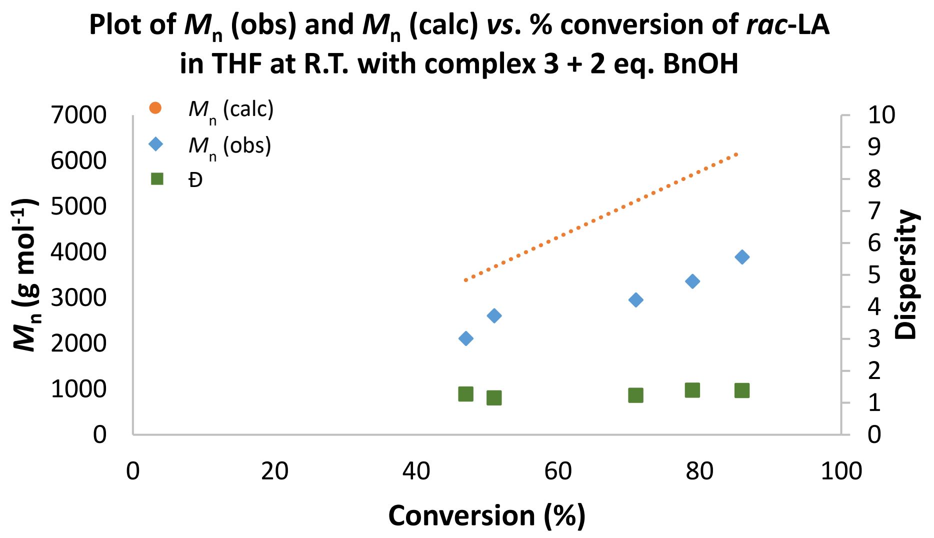

Figure S18. Comparison between experimental and calculated M_n values and dispersity values at increasing conversions of *rac*-LA in presence of complex **3** + 2 eq. BnOH in THF at R.T.

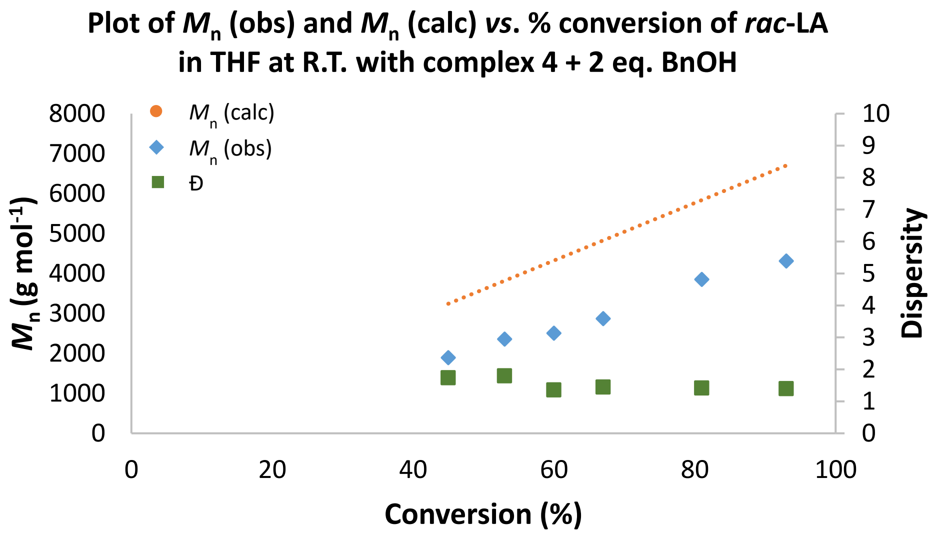

Figure S19. Comparison between experimental and calculated M_n values and dispersity values at increasing conversions of *rac*-LA in presence of complex **4** + 2 eq. BnOH in THF at R.T.

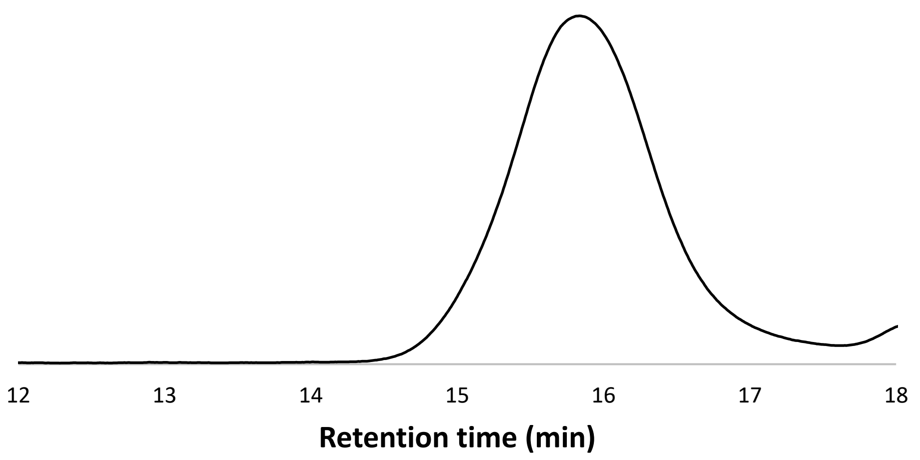

Figure S20. Example GPC trace of PLA generated in presence of complex **3** + 2 eq. BnOH in THF at R.T. (Entry 5, Table S1).

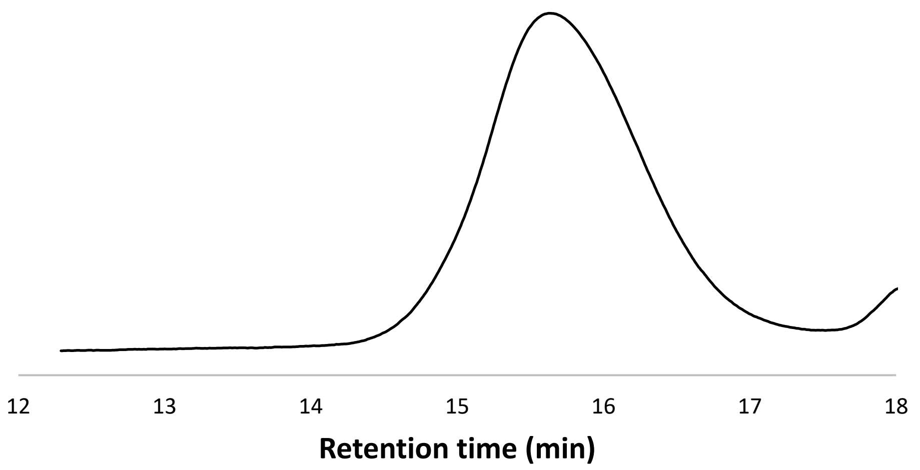

Figure S21. Example GPC trace of PLA generated in presence of complex **4** + 2 eq. BnOH in THF at R.T. (Entry 15, Table S1).

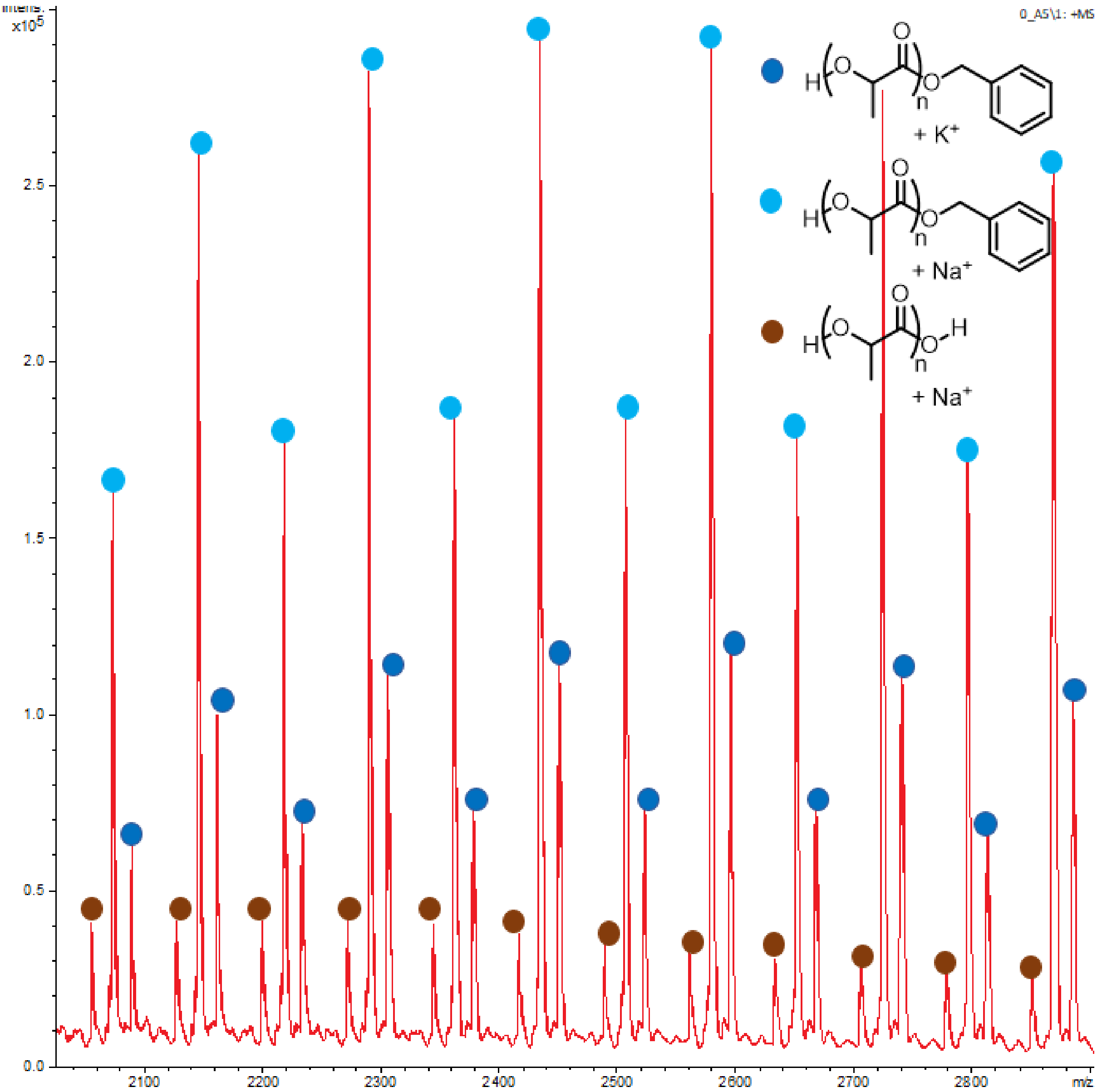

Figure S22. MALDI-TOF spectrum of PLA resulting from 47 % conversion of *rac*-LA in the presence of complex **3** + 2 eq. BnOH (THF, R.T.).

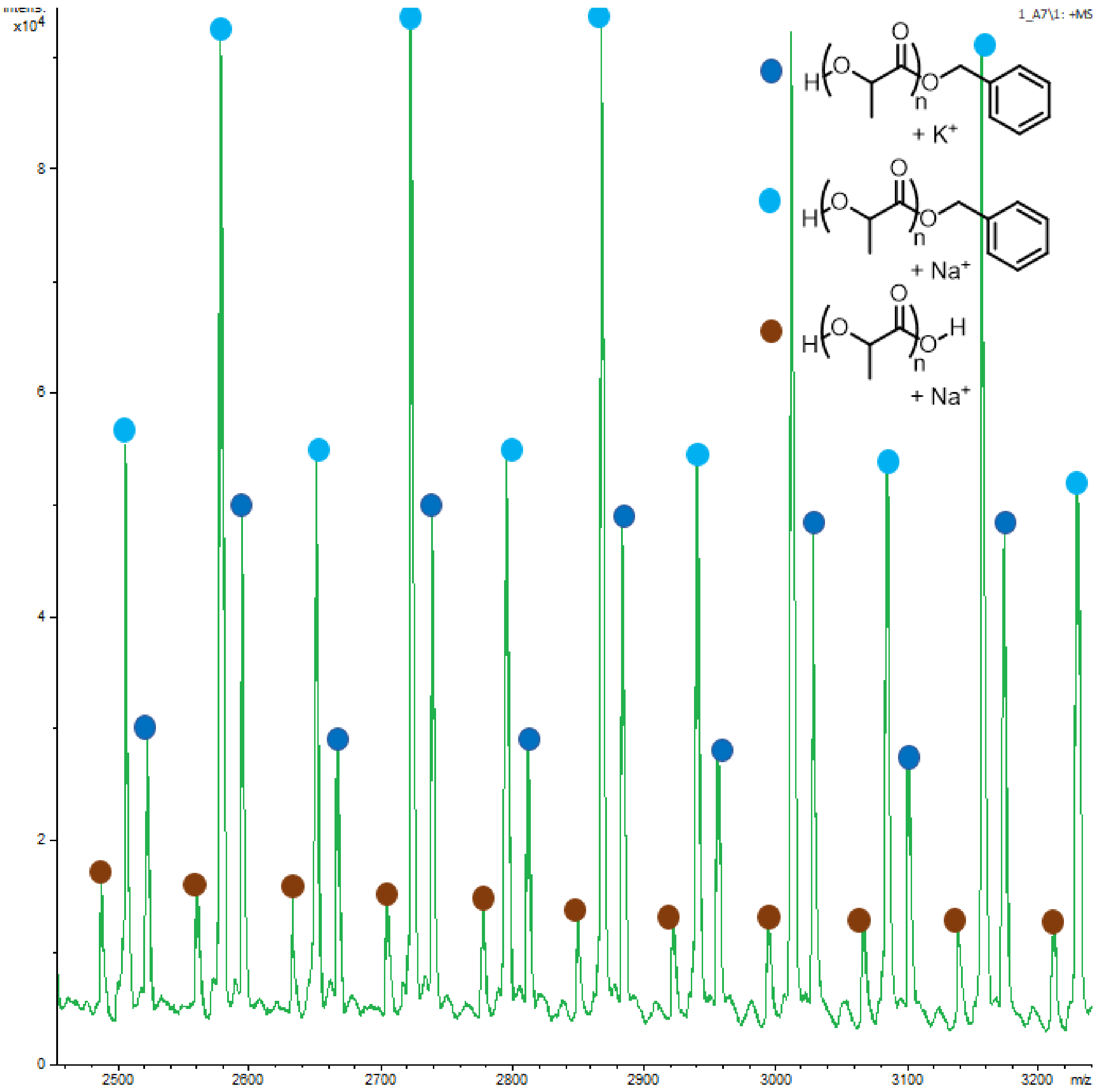

Figure S23. MALDI-TOF spectrum of PLA resulting from 71 % conversion of *rac*-LA in the presence of complex **3** + 2 eq. BnOH (THF, R.T.).

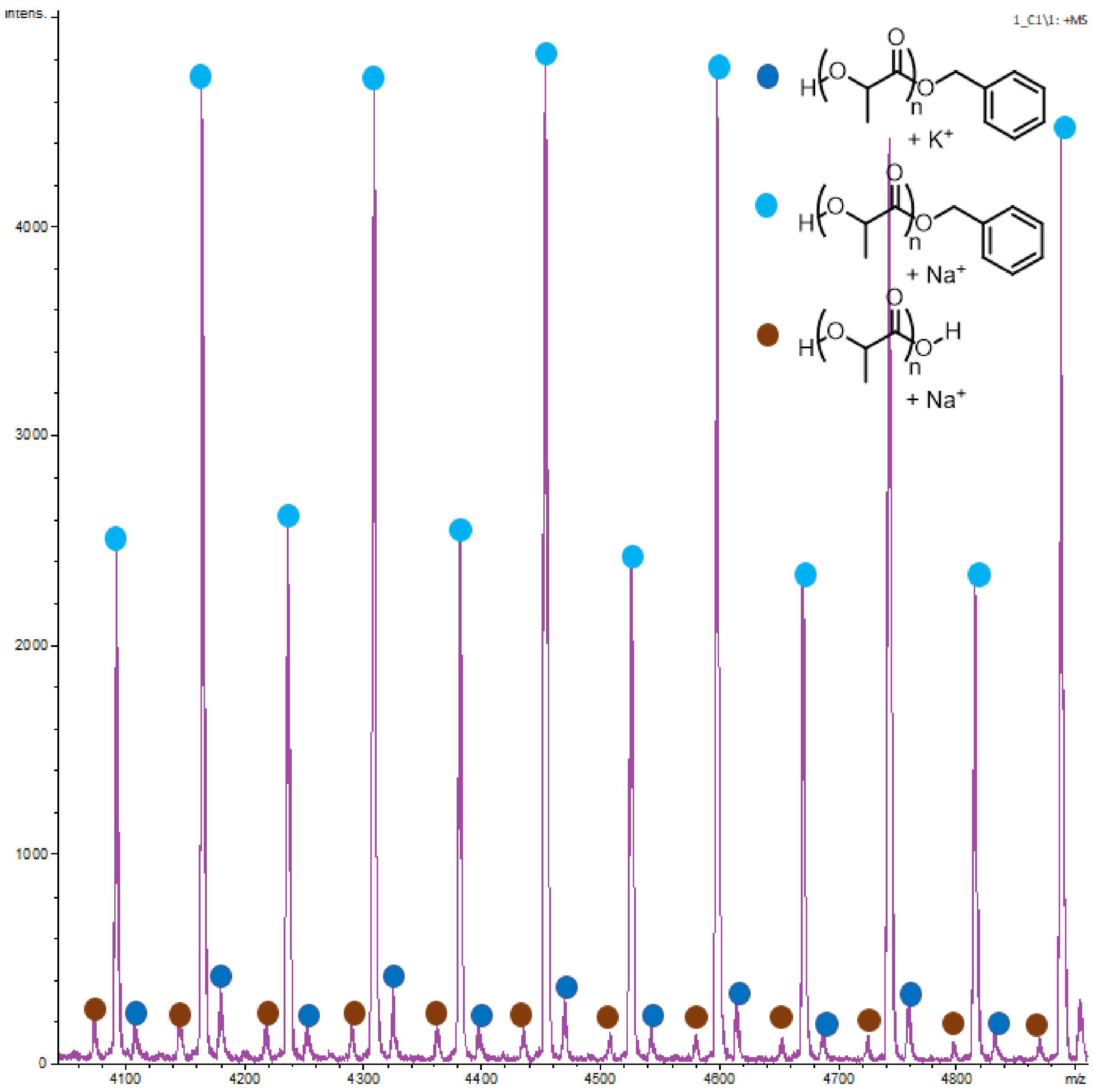

Figure S24. MALDI-TOF spectrum of PLA resulting from 86 % conversion of *rac*-LA in the presence of complex **3** + 2 eq. BnOH (THF, R.T.).

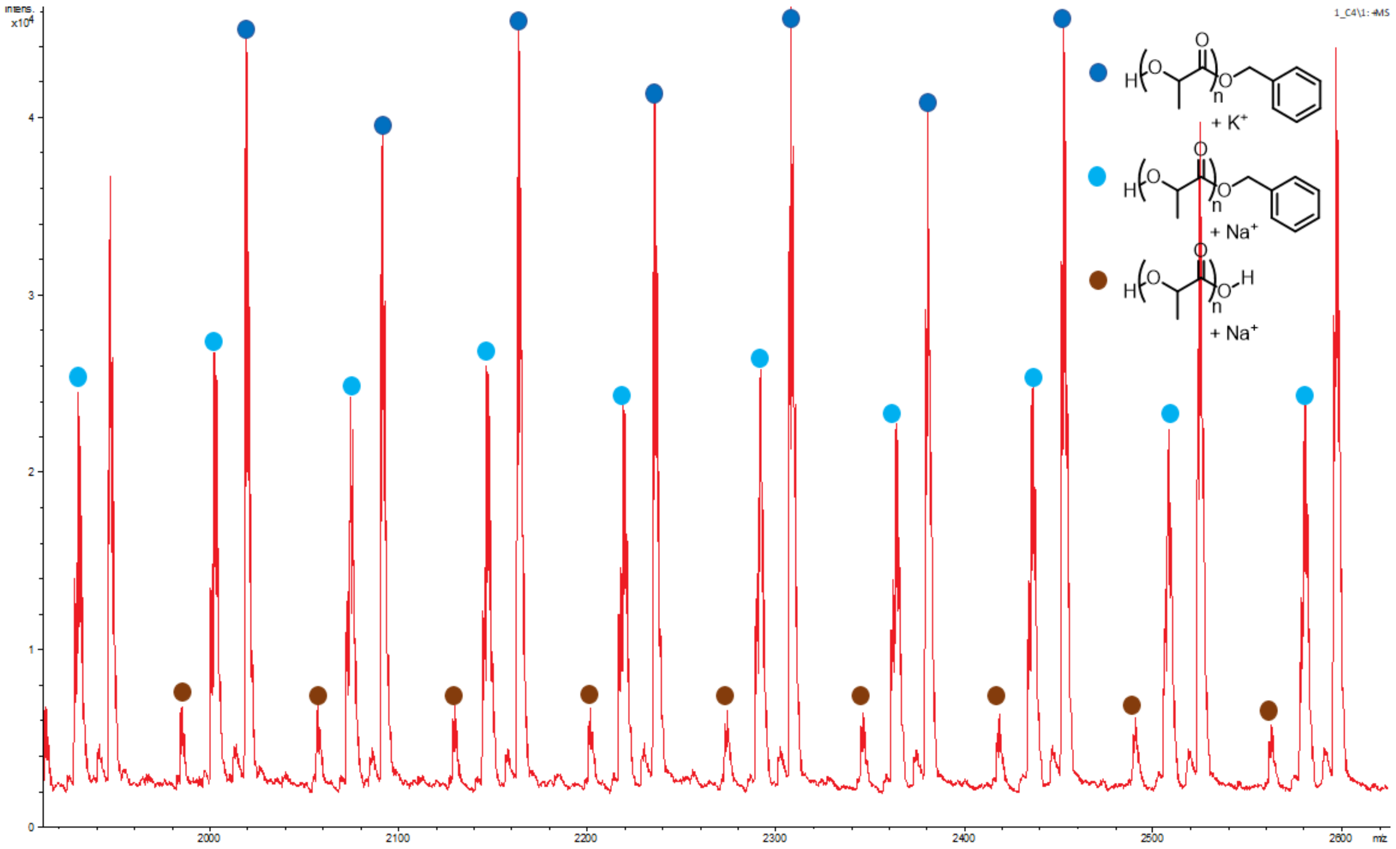

Figure S25. MALDI-TOF spectrum of PLA resulting from 53 % conversion of *rac*-LA in the presence of complex **4** + 2 eq. BnOH (THF, R.T.).

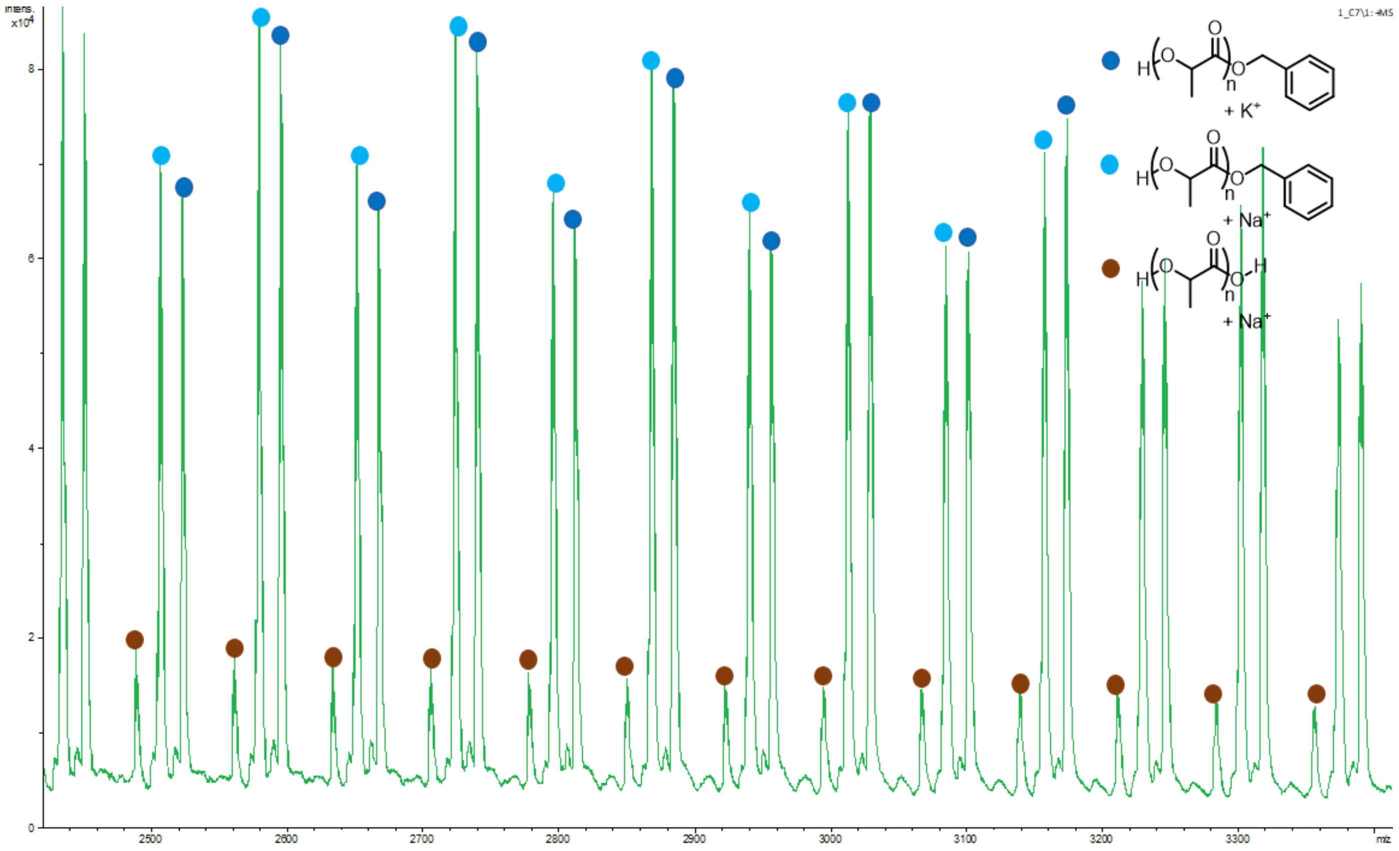

Figure S26. MALDI-TOF spectrum of PLA resulting from 67 % conversion of *rac*-LA in the presence of complex **4** + 2 eq. BnOH (THF, R.T.).

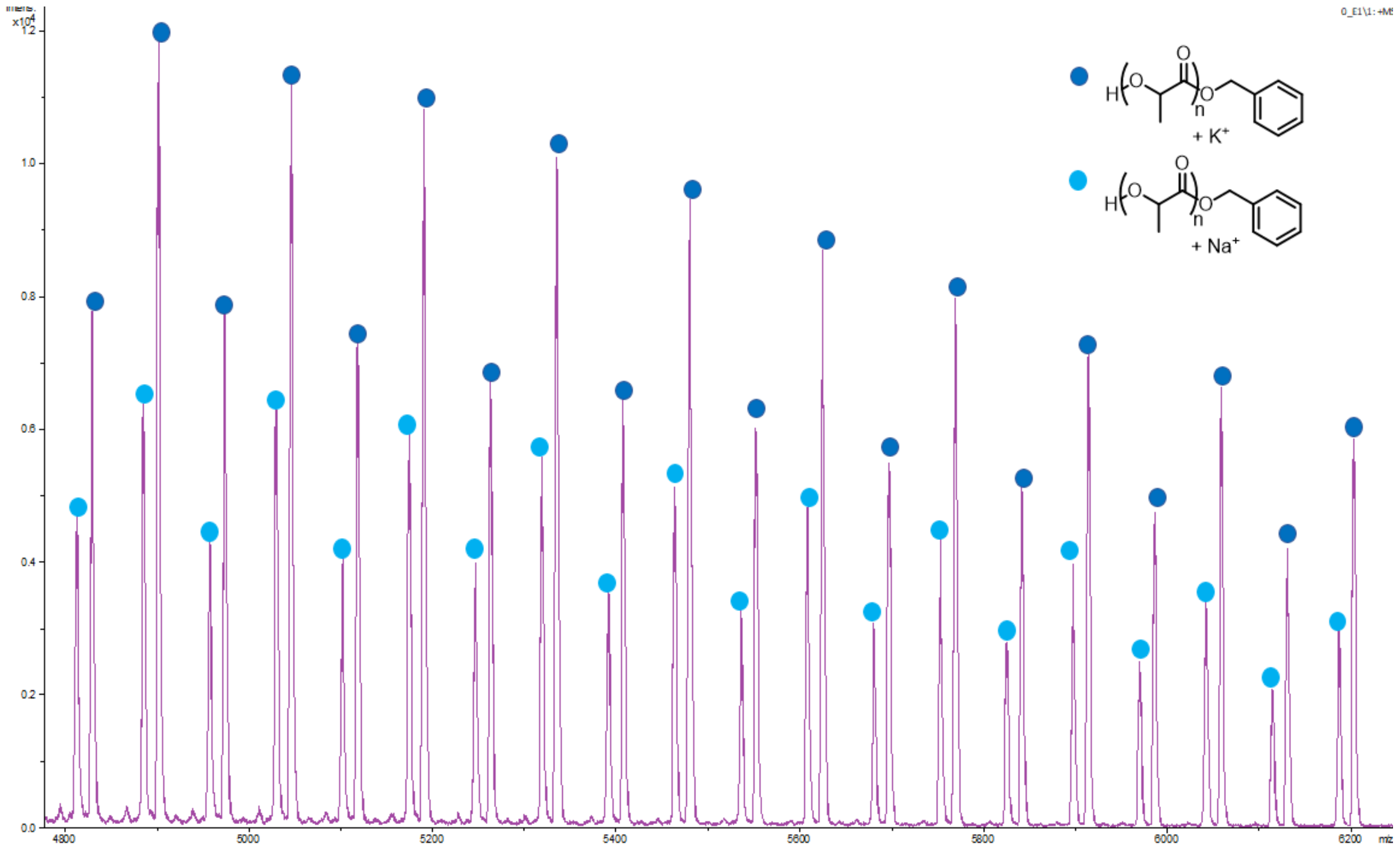

Figure S27. MALDI-TOF spectrum of PLA resulting from 93 % conversion of *rac*-LA in the presence of complex **4** + 2 eq. BnOH (THF, R.T.).

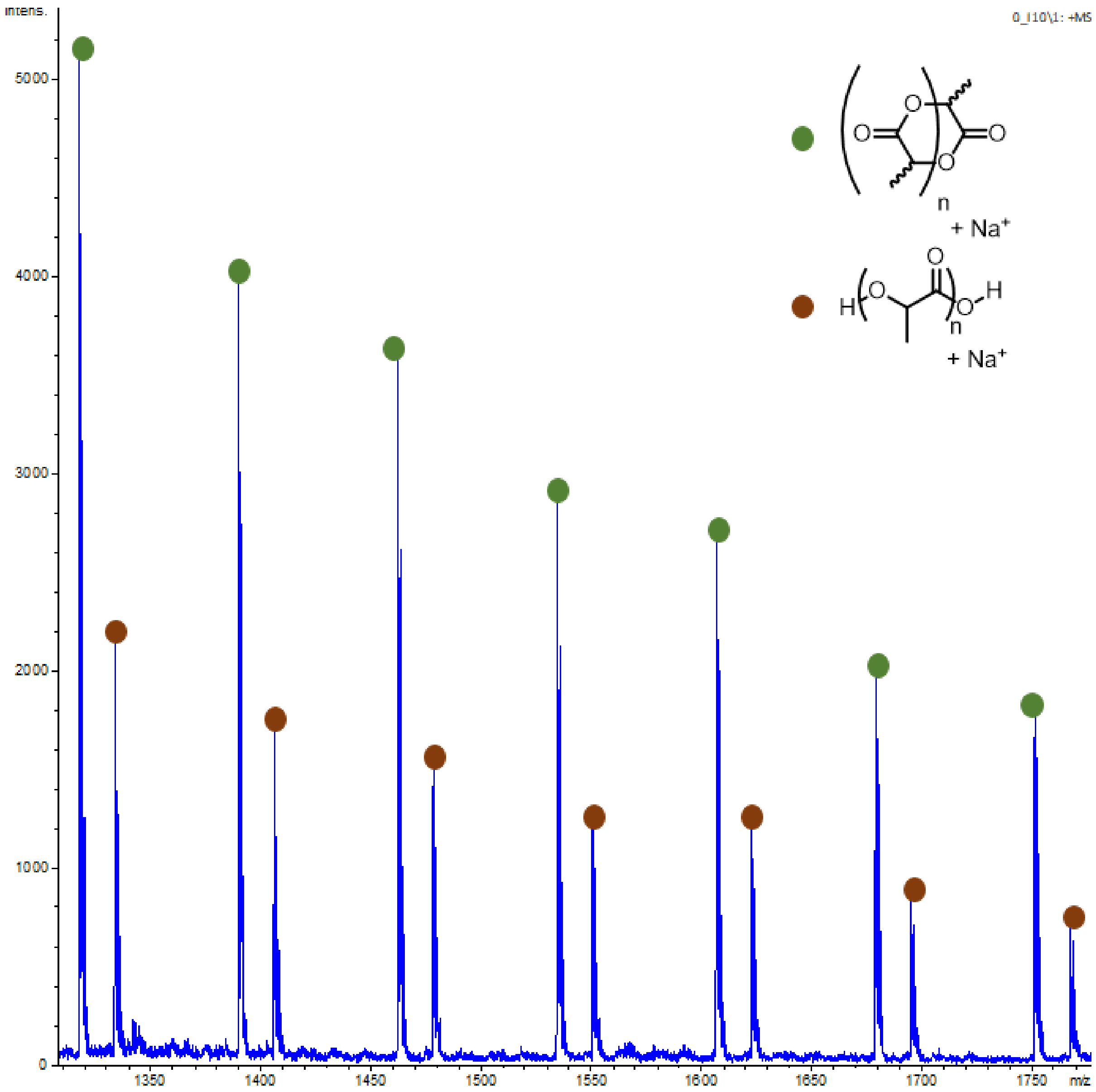

Figure S28. MALDI-TOF spectrum of PLA resulting from 79 % conversion of *rac*-LA in the presence of homometallic complex **1** (THF, R.T.).

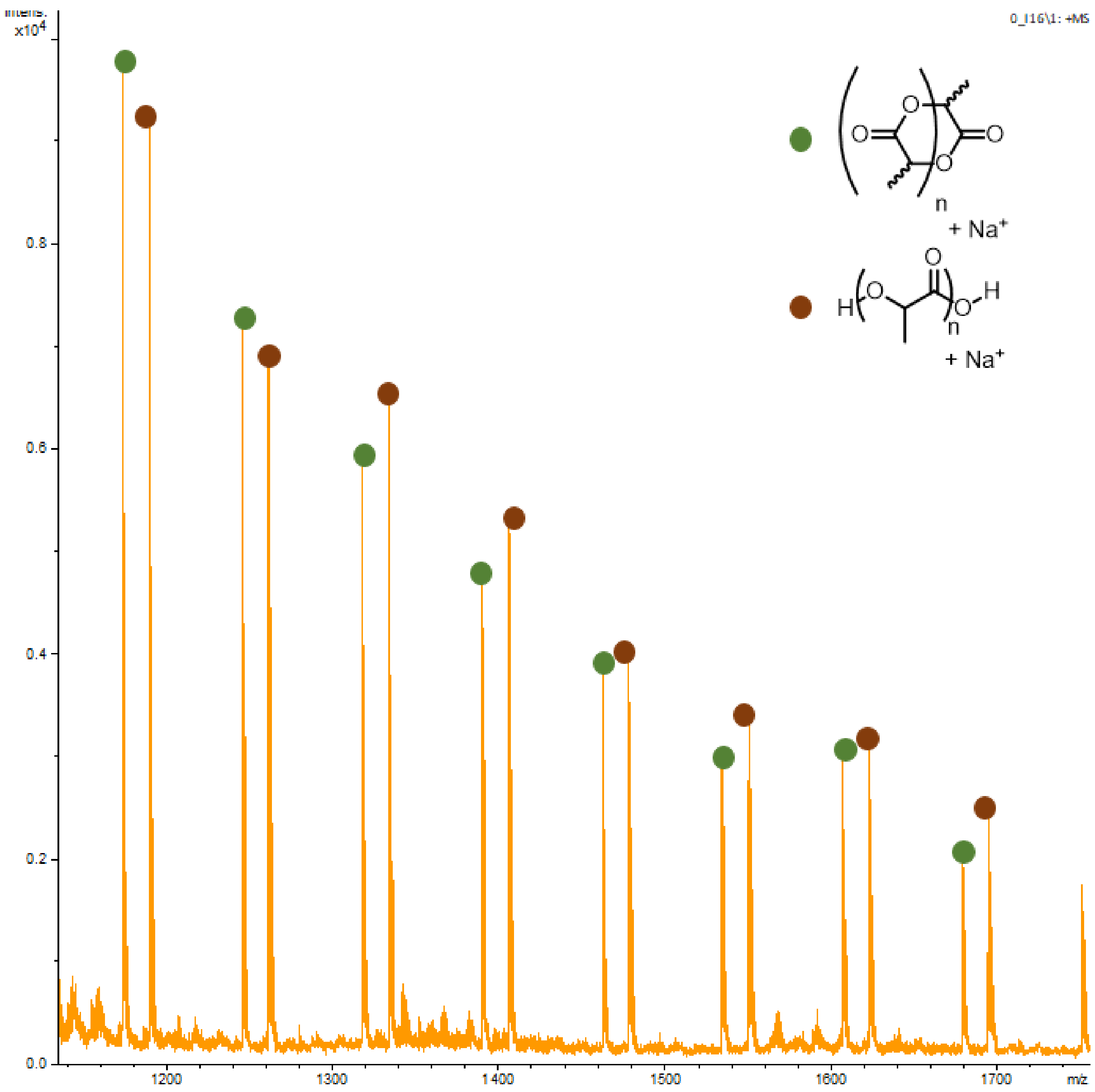

Figure S29. MALDI-TOF spectrum of PLA resulting from 72 % conversion of *rac*-LA in the presence of homometallic complex **2** (THF, R.T.).

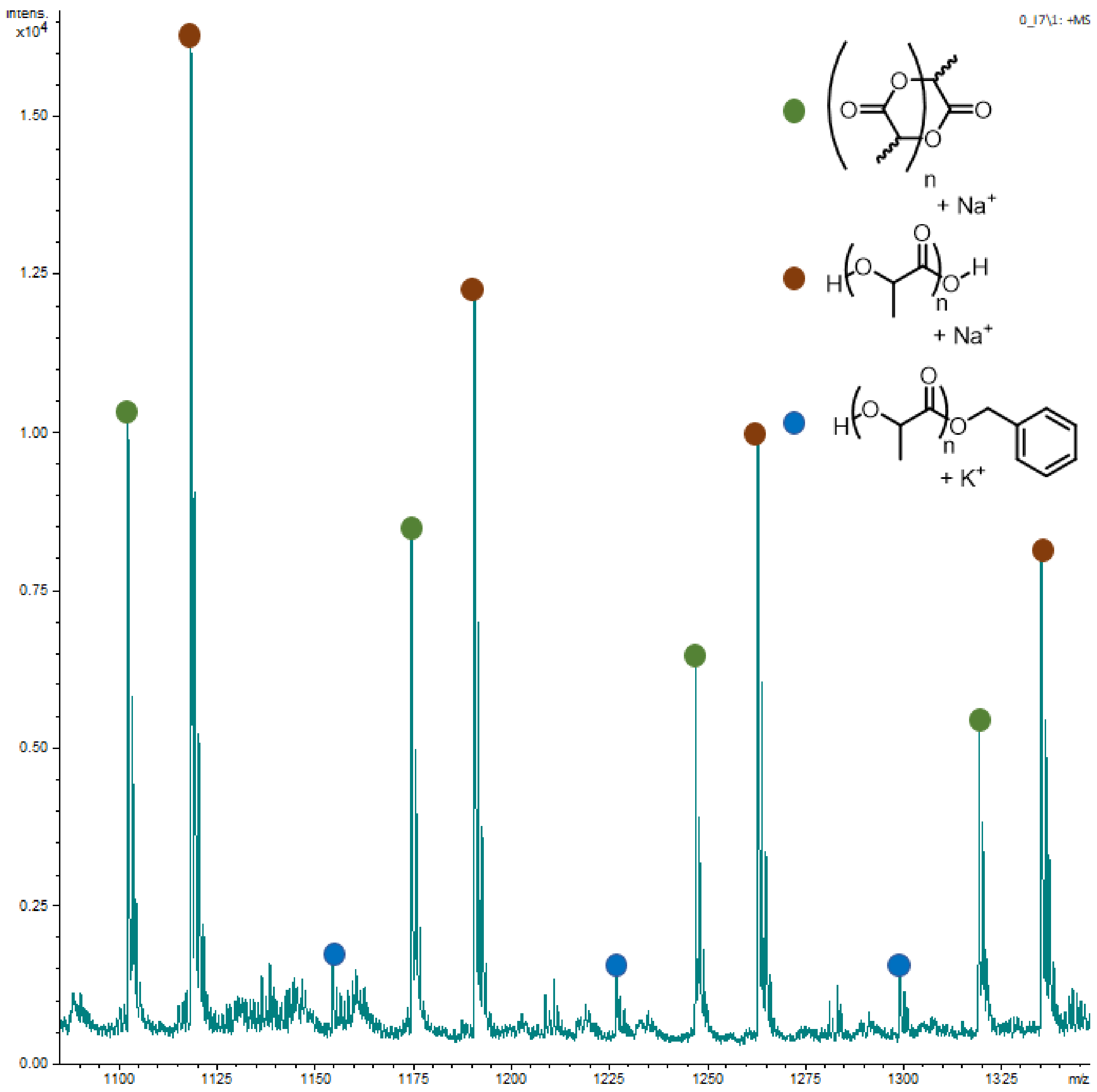

Figure S30. MALDI-TOF spectrum of PLA resulting from 88 % conversion of *rac*-LA in the presence of **[BnONa]** (THF, R.T.).

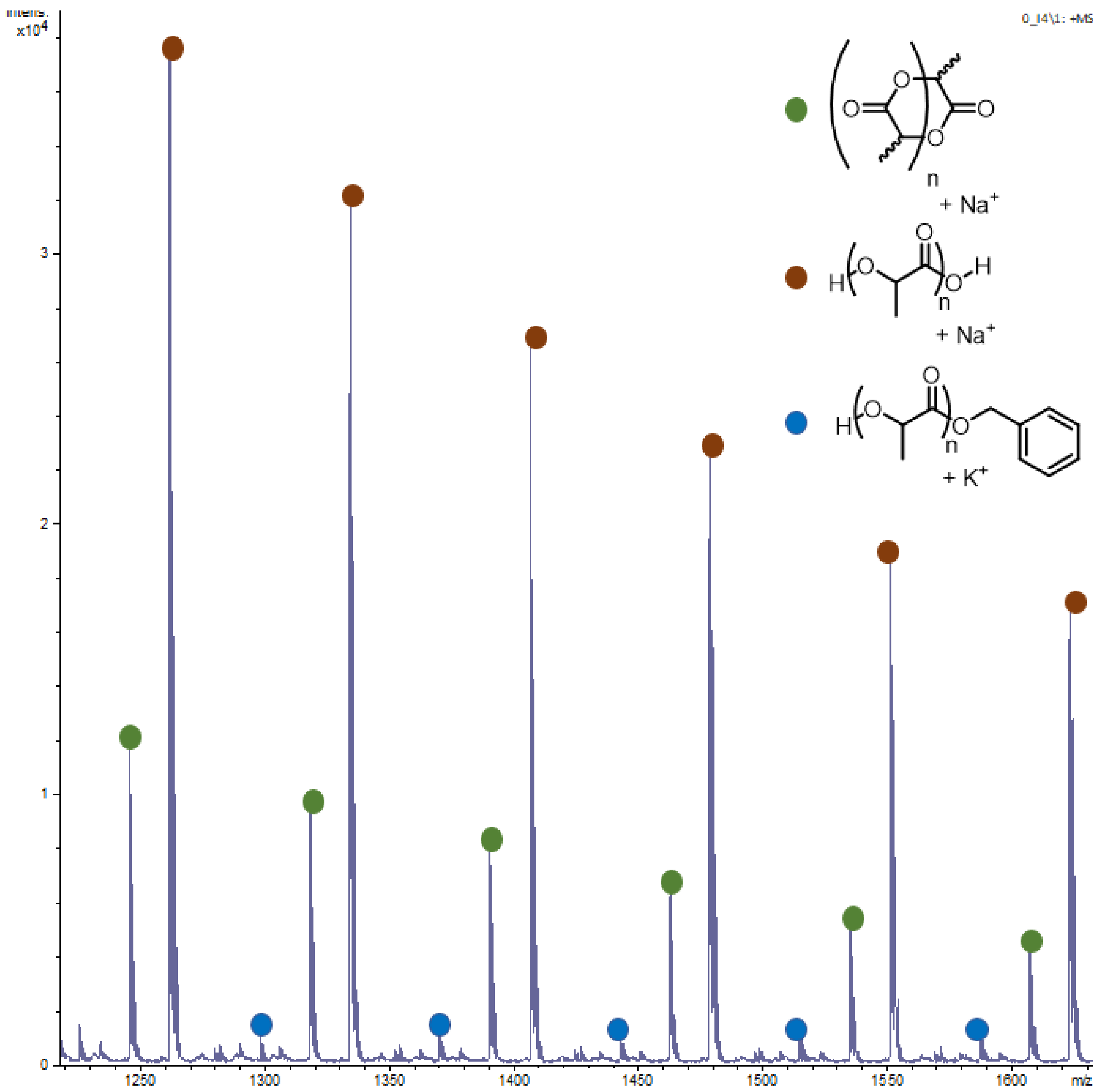

Figure S31. MALDI-TOF spectrum of PLA resulting from 94 % conversion of *rac*-LA in the presence of [BnOK] (THF, R.T.).

Table S2. ROP of *rac*-LA catalysed by complexes **3** and **4** in the presence of 2 eq. of BnOH in THF at 60 °C.

Entry	Cat.	Time (min)	Conv.[a] (%)	$M_{n,obs}$[b] (Da)	$M_{n,calc}$[c] (Da)	*Đ*[b]	P_i[d]
**1**[e]	**3**	2.5	12	-	-	-	-
**2**	**3**	0.08	40	1500	2900	1.13	0.49
**3**	**3**	0.67	64	3000	4600	1.19	-
**4**	**3**	2.5	87	3900	6300	1.48	-
**5**[e]	**4**	2.5	13	-	-	-	-
**6**	**4**	0.08	66	3200	4800	1.76	0.49
**7**	**4**	0.33	85	4100	6100	1.86	0.53
**8**	**4**	0.67	94	4600	6800	1.71	0.52

[LA] = 1 M in THF.[a] Conversion calculated using ^{1}H NMR spectroscopy.[b] $M_{n,obs}$ and Đ determined by gel permeation chromatography using polystyrene standards in THF. Values corrected by Mark-Houwink factor (0.58).[9] [c] $M_{n,calc}$ of polymers calculated from the monomer conversion $M_{n,calc}$ = M_0 x ([M]/[I]) x conversion assuming 2 chains per catalyst.[d] Determined by homodecoupled ^{1}H NMR spectroscopy.[e] No BnOH used.

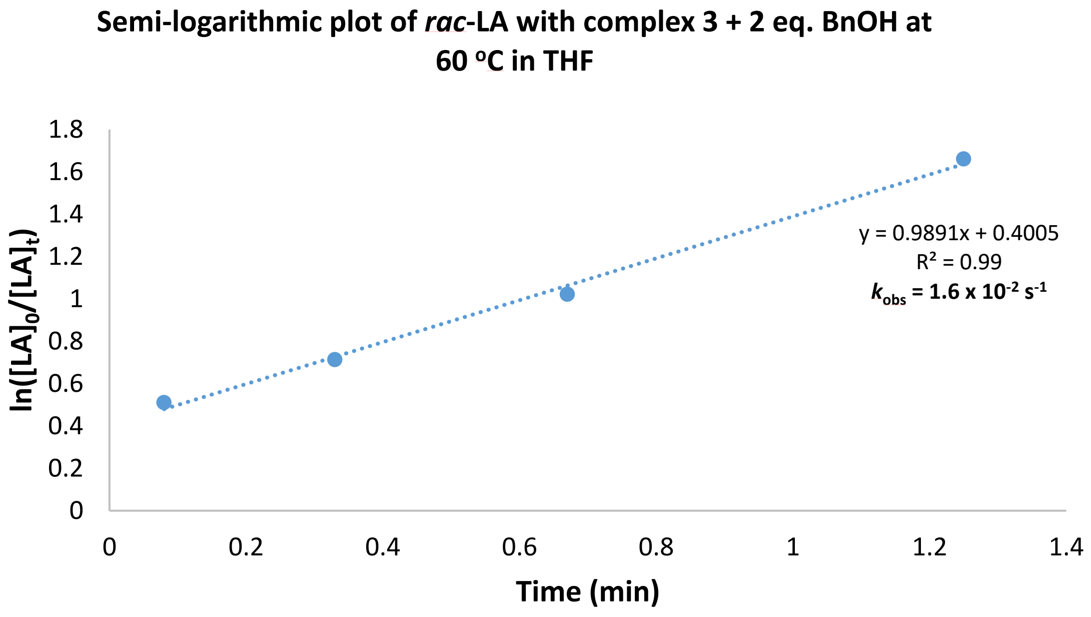

Figure S32. Plot of ln($[LA]_0$/$[LA]_t$) *vs*. time (min) for ROP of *rac*-LA with complex **3** and 2 eq. BnOH ([LA] = 1 M in THF, 60 °C).

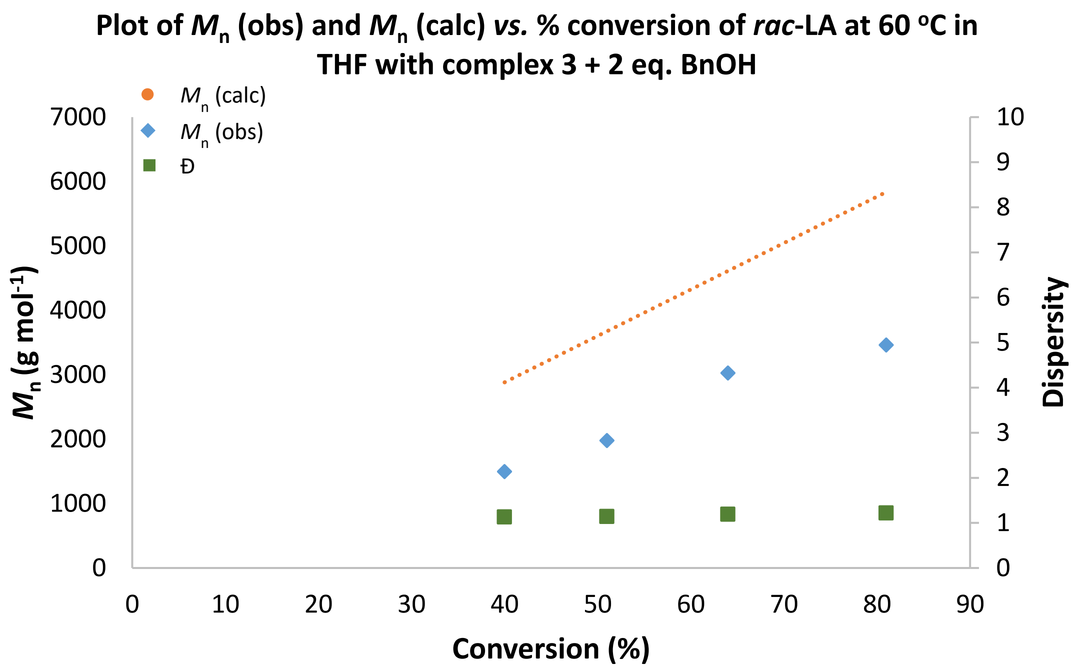

Figure S33. Comparison between experimental and calculated M_n values and dispersity values at increasing conversions of *rac*-LA in the presence of complex **3** + 2 eq. BnOH in THF at 60 °C.

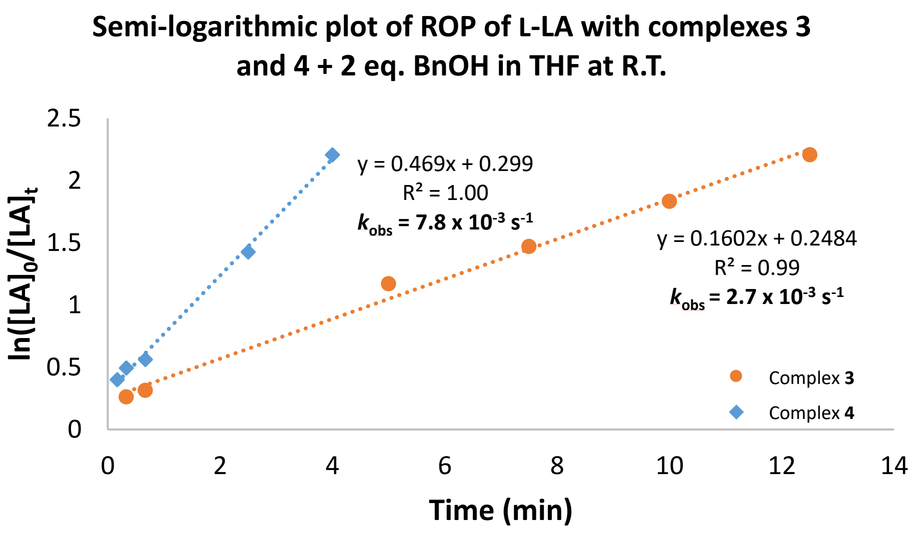

Figure S34. Plot of $\ln([LA]_0/[LA]_t)$ *vs*. time (min) for ROP of L-LA with complexes **3** and **4** and 2 eq. BnOH ([LA] = 1 M in THF, R.T.).

Table S3. ROP of *rac*-LA catalysed by complexes **3** and **4** in the presence of 2 eq. of BnOH in toluene at 60 °C.

Entry	Cat.	Time (min)	Conv.[a] (%)	$M_{n,obs}$[b] (Da)	$M_{n,calc}$[c] (Da)	$Đ$[b]	P_i[d]
**1**[e]	**3**	2.5	10	-	-	-	-
**2**	**3**	0.08	35	1100	2500	1.17	-
**3**	**3**	0.33	49	1400	3500	1.15	-
**4**	**3**	0.67	56	2000	4000	1.17	0.48
**5**	**3**	1.25	74	2300	5300	1.19	0.49
**6**	**3**	2.5	89	2600	6400	1.17	0.51
**7**[e]	**4**	1	8	-	-	-	-
**8**	**4**	0.08	68	2300	4900	1.28	0.48
**9**	**4**	0.33	74	2900	5300	1.41	0.55
**10**	**4**	1	86	3900	6200	1.39	0.55
**11**	**4**	2	99	3800	7100	1.61	0.54
**12**[f]	**[LZn$_2$OBn]**	5	56	4500	8100[g]	1.22	0.48
**13**[f]	**[LZn$_2$OBn]**	10	87	8100	12500[g]	1.07	0.50
**14**[e,h]	**[LZn$_2$OBn]**	0.33	66	12000	9500[g]	1.42	-
**15**[e,h]	**[LZn$_2$OBn]**	2	99	19900	14300[g]	1.35	-
**16**[e,i]	**[BnOK] + [LZn$_2$OBn]**	0.33	82	8400	5900	2.00	-
**17**[e,i]	**[BnOK] + [LZn$_2$OBn]**	2	88	7400	6300	1.69	-

[LA] = 1 M in toluene. LA and pre-catalyst pre-stirred separately for 3 min in toluene at 60 °C before mixing and initiation with BnOH. [a] Conversion calculated using ^{1}H NMR spectroscopy. [b] $M_{n,obs}$ and $Đ$ determined by gel permeation chromatography using polystyrene standards in THF. Values corrected by Mark-Houwink factor (0.58).[9] [c] $M_{n,calc}$ of polymers calculated from the monomer conversion $M_{n,calc}$ = M_0 x ([M]/[I]) x conversion assuming 2 chains per catalyst. [d] Determined by homodecoupled ^{1}H NMR spectroscopy. [e] No BnOH used. [f] **[LZn$_2$OBn]** generated *in situ* from **[LZn$_2$Et]** + 1 eq. BnOH. [g] $M_{n,calc}$ of polymers calculated from the monomer conversion $M_{n,calc}$ = M_0 x ([M]/[I]) x conversion assuming 1 chain per catalyst. [h] Isolated **[LZn$_2$OBn]** used. [i] **[BnOK]** *in situ* generated from KH and BnOH before mixing with isolated **[LZn$_2$OBn]**.

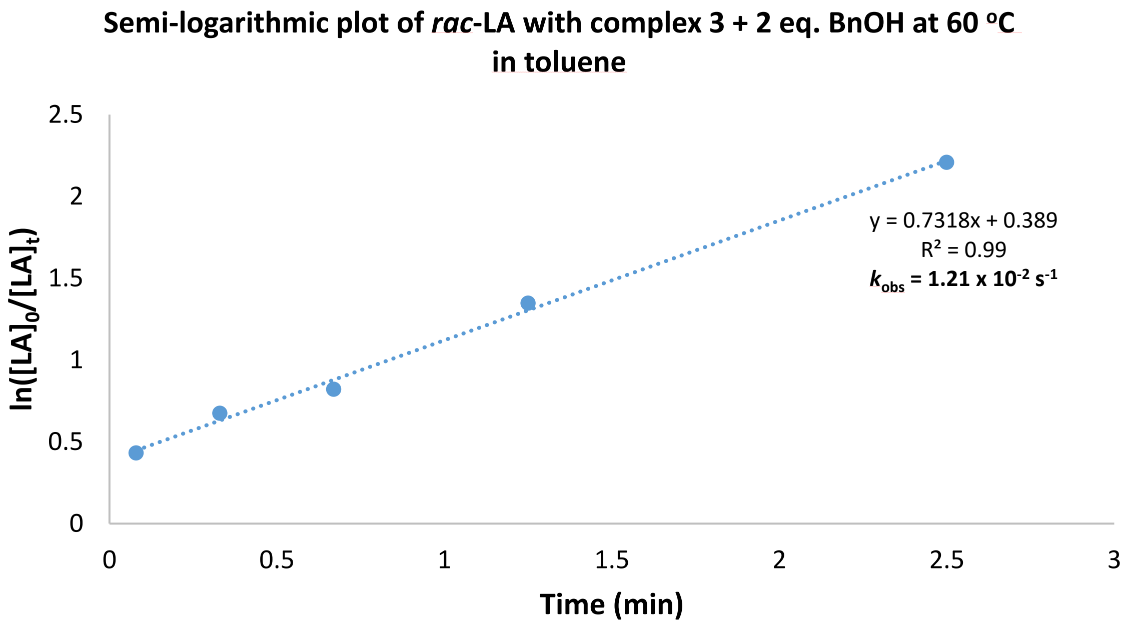

Figure S35. Plot of ln($[LA]_0/[LA]_t$) *vs*. time (min) for ROP of *rac*-LA with complex **3** and 2 eq. BnOH ([LA] = 1 M in toluene, 60 °C).

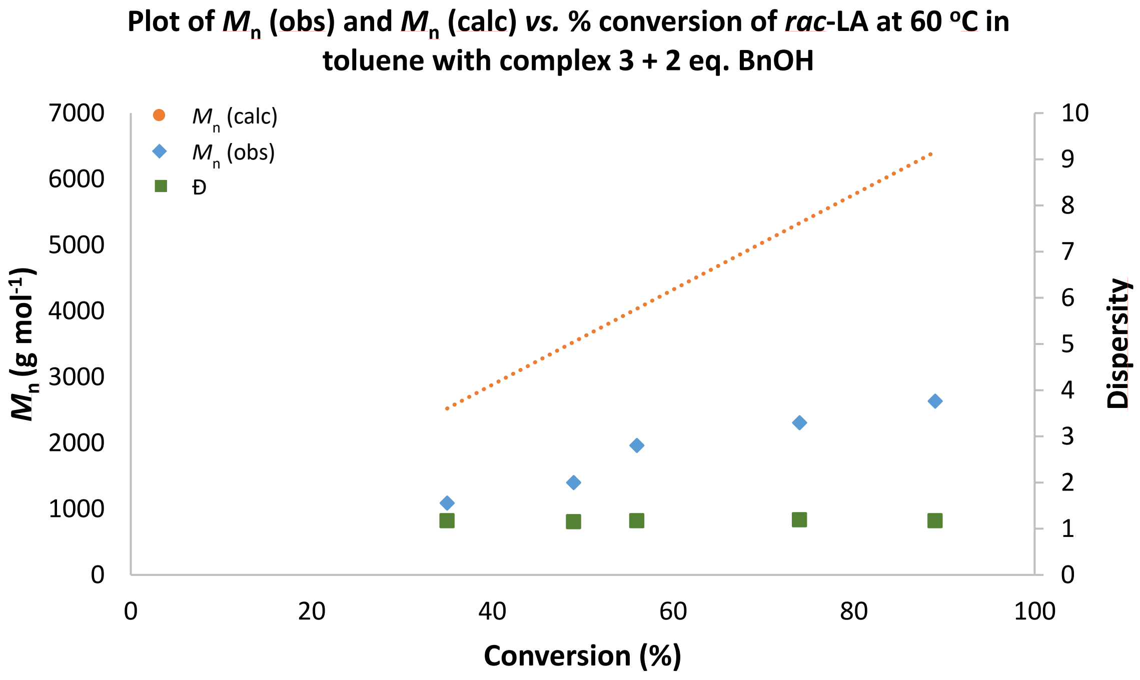

Figure S36. Comparison between experimental and calculated M_n values and dispersity values at increasing conversions of *rac*-LA in the presence of complex **3** + 2 eq. BnOH in toluene at 60 °C.

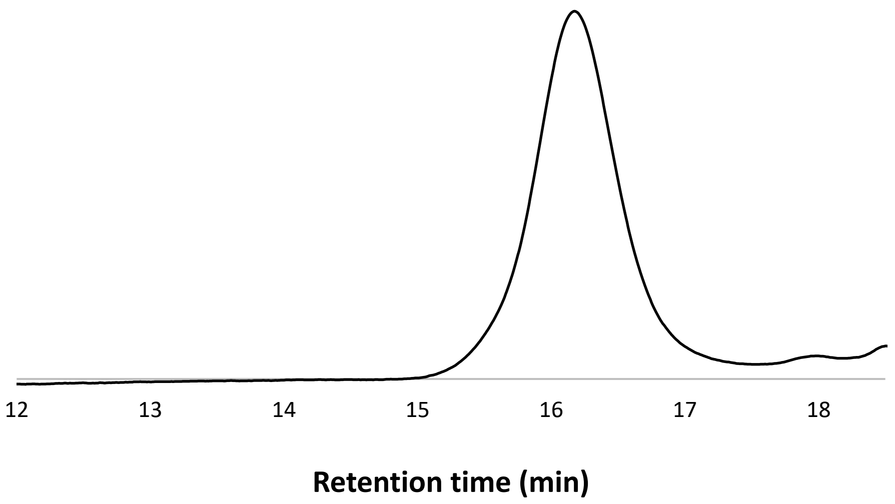

Figure S37. Example GPC trace of PLA generated in the presence of complex **3** + 2 eq. BnOH in toluene at 60 °C (Entry 6, Table S3).

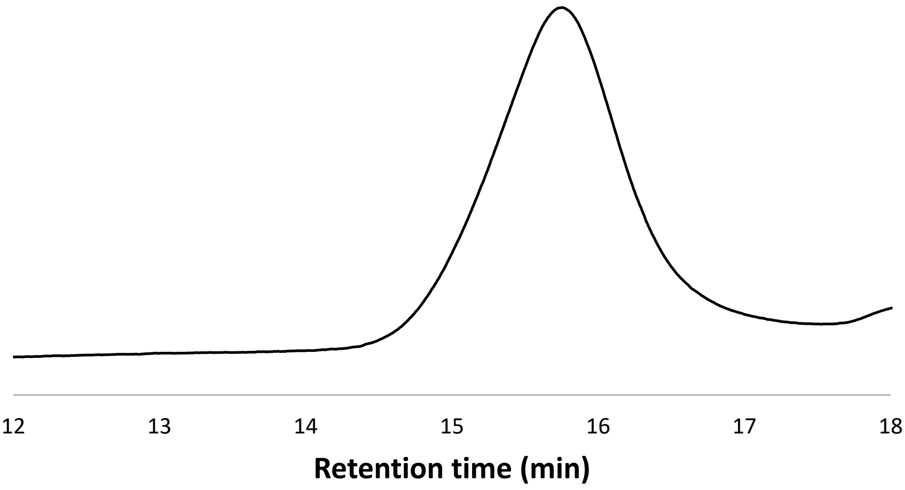

Figure S38. Example GPC trace of PLA generated in the presence of complex **4** + 2 eq. BnOH in toluene at 60 °C (Entry 10, Table S3).

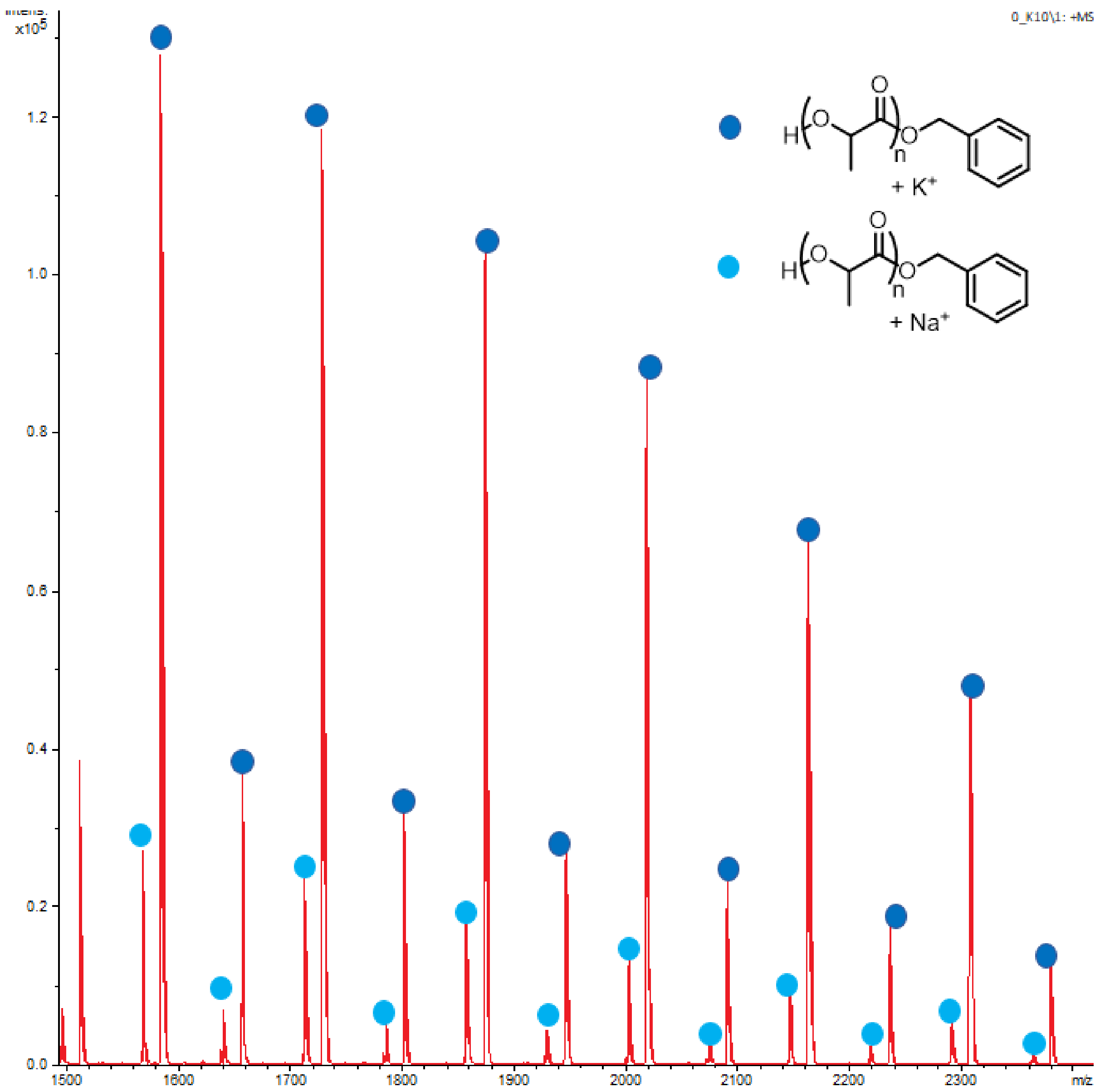

Figure S39. MALDI-TOF spectrum of PLA resulting from 35 % conversion of *rac*-LA in the presence of complex **3** + 2 eq. BnOH (toluene, 60 °C).

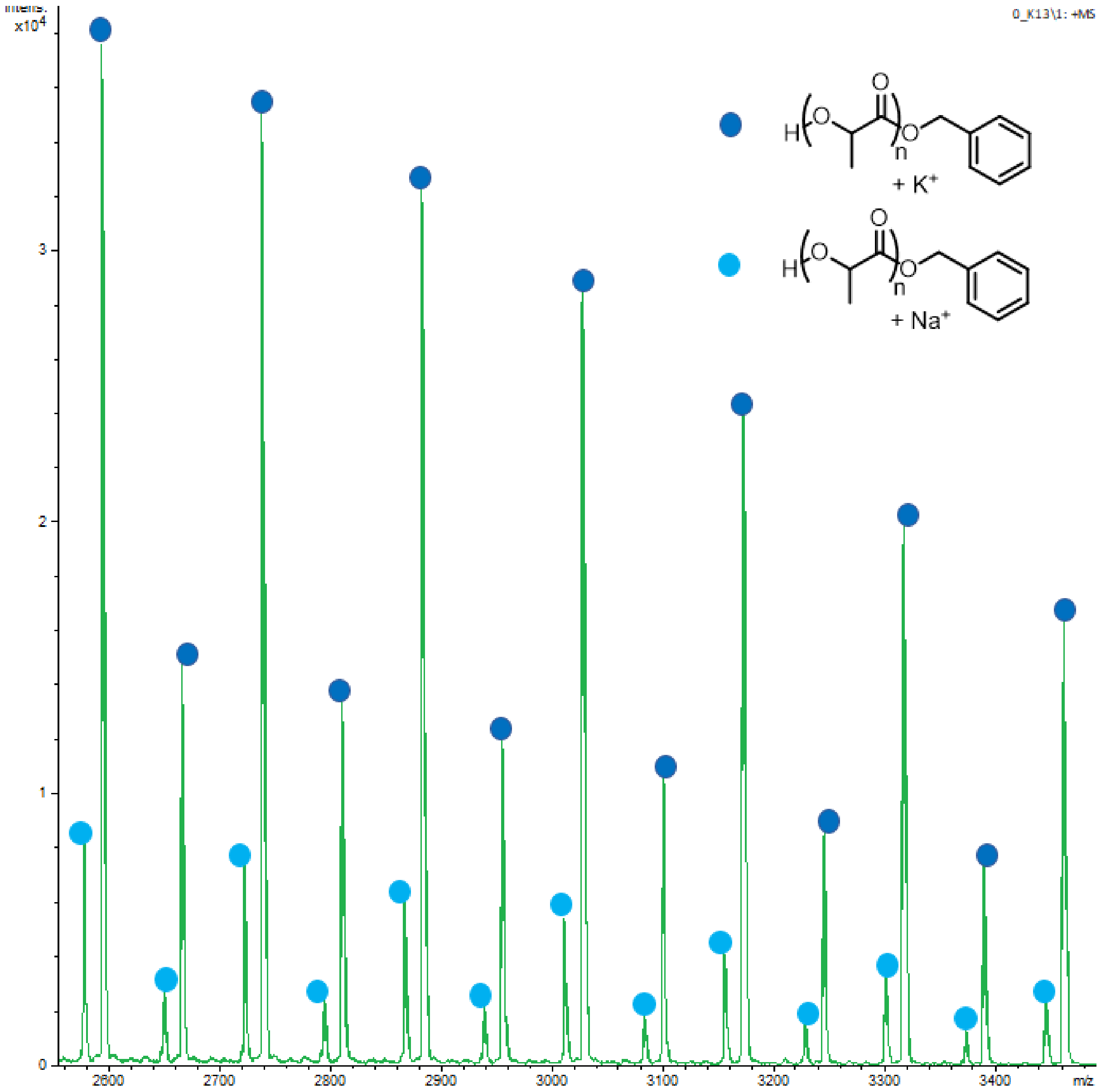

Figure S40. MALDI-TOF spectrum of PLA resulting from 56 % conversion of *rac*-LA in the presence of complex **3** + 2 eq. BnOH (toluene, 60 °C).

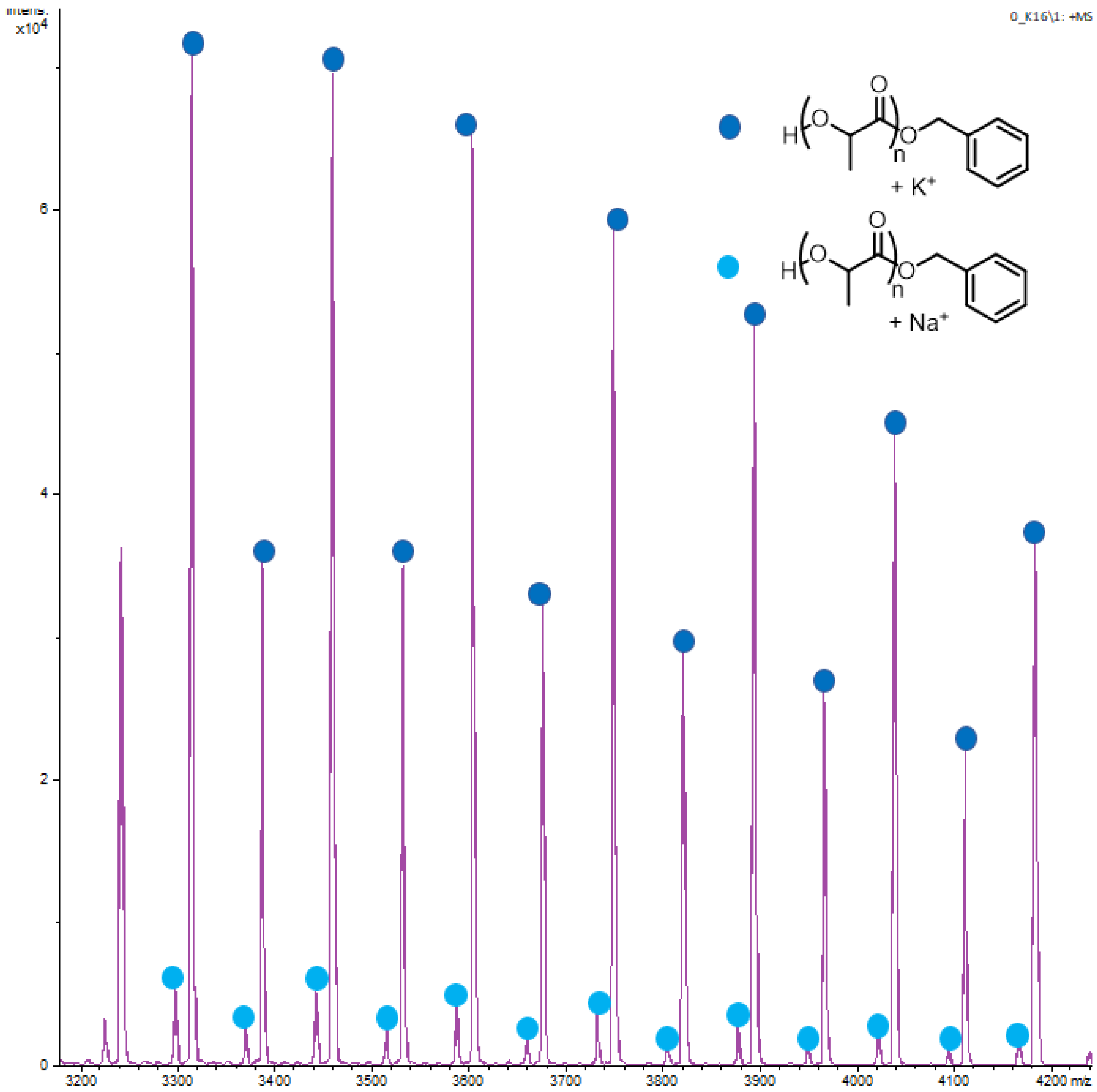

Figure S41. MALDI-TOF spectrum of PLA resulting from 89 % conversion of *rac*-LA in the presence of complex **3** + 2 eq. BnOH (toluene, 60 °C).

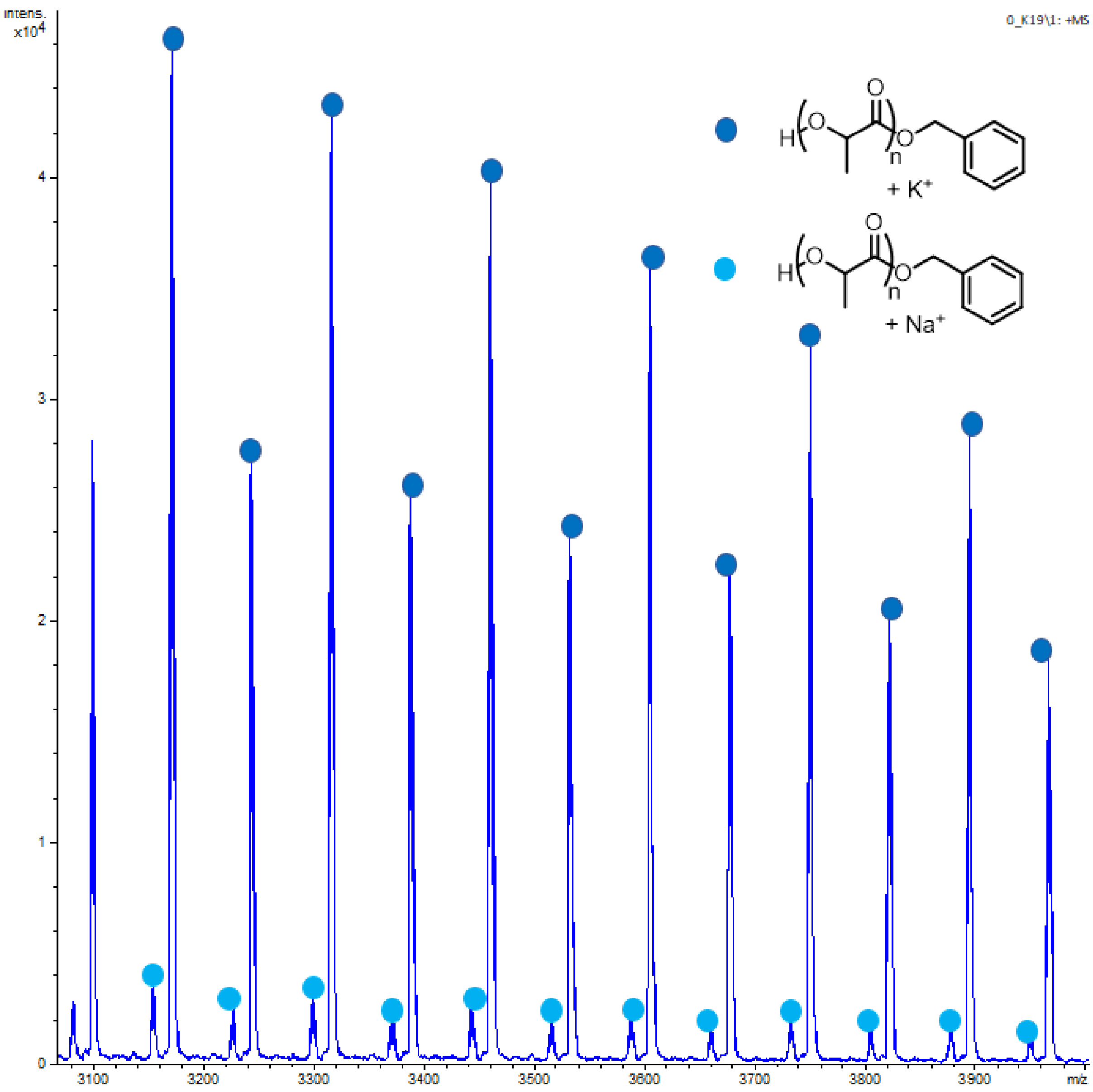

Figure S42. MALDI-TOF spectrum of PLA resulting from 68 % conversion of *rac*-LA in the presence of complex **4** + 2 eq. BnOH (toluene, 60 °C).

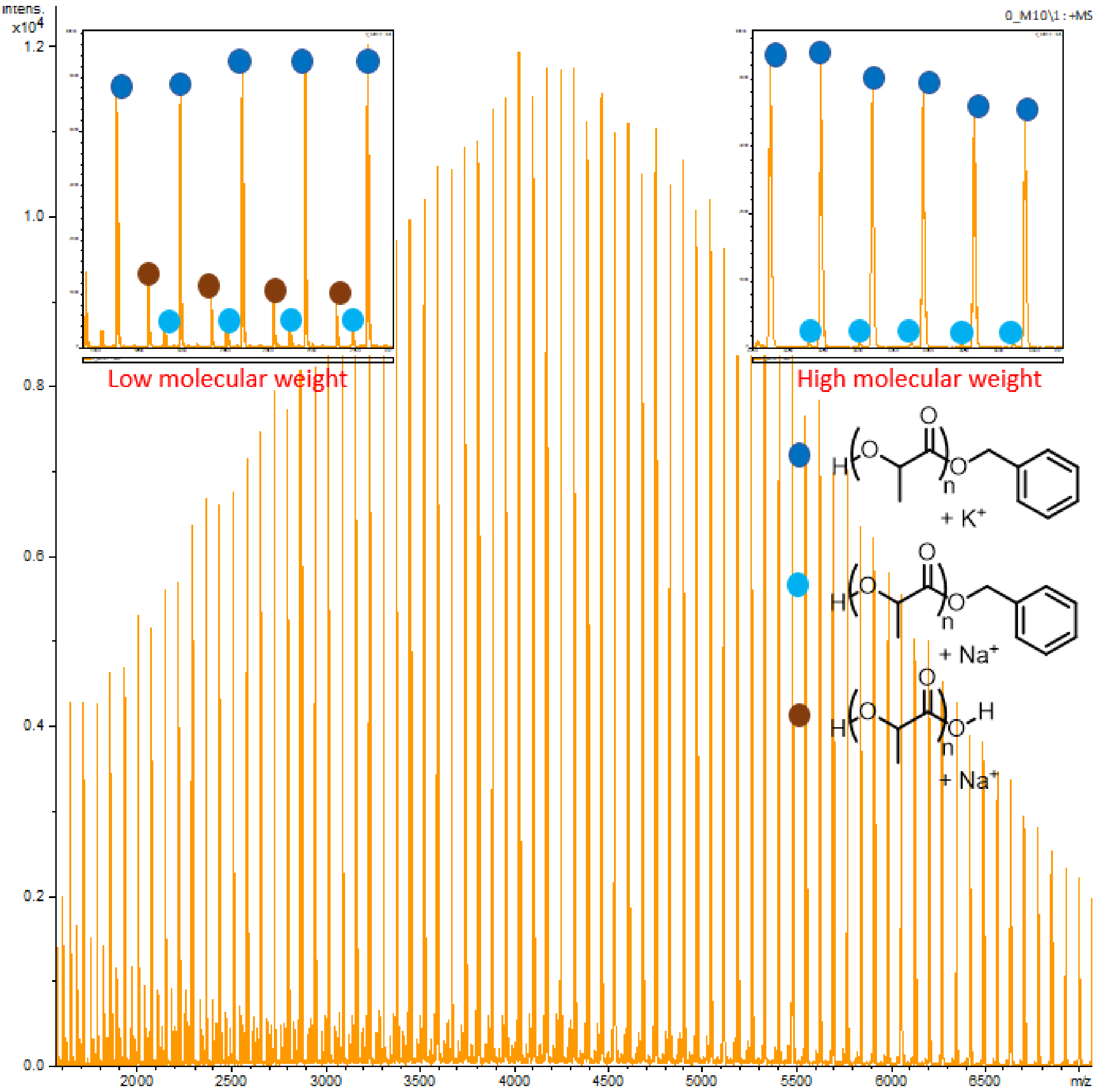

Figure S43. MALDI-TOF spectrum of PLA resulting from 86 % conversion of *rac*-LA in the presence of complex **4** + 2 eq. BnOH (toluene, 60 °C).

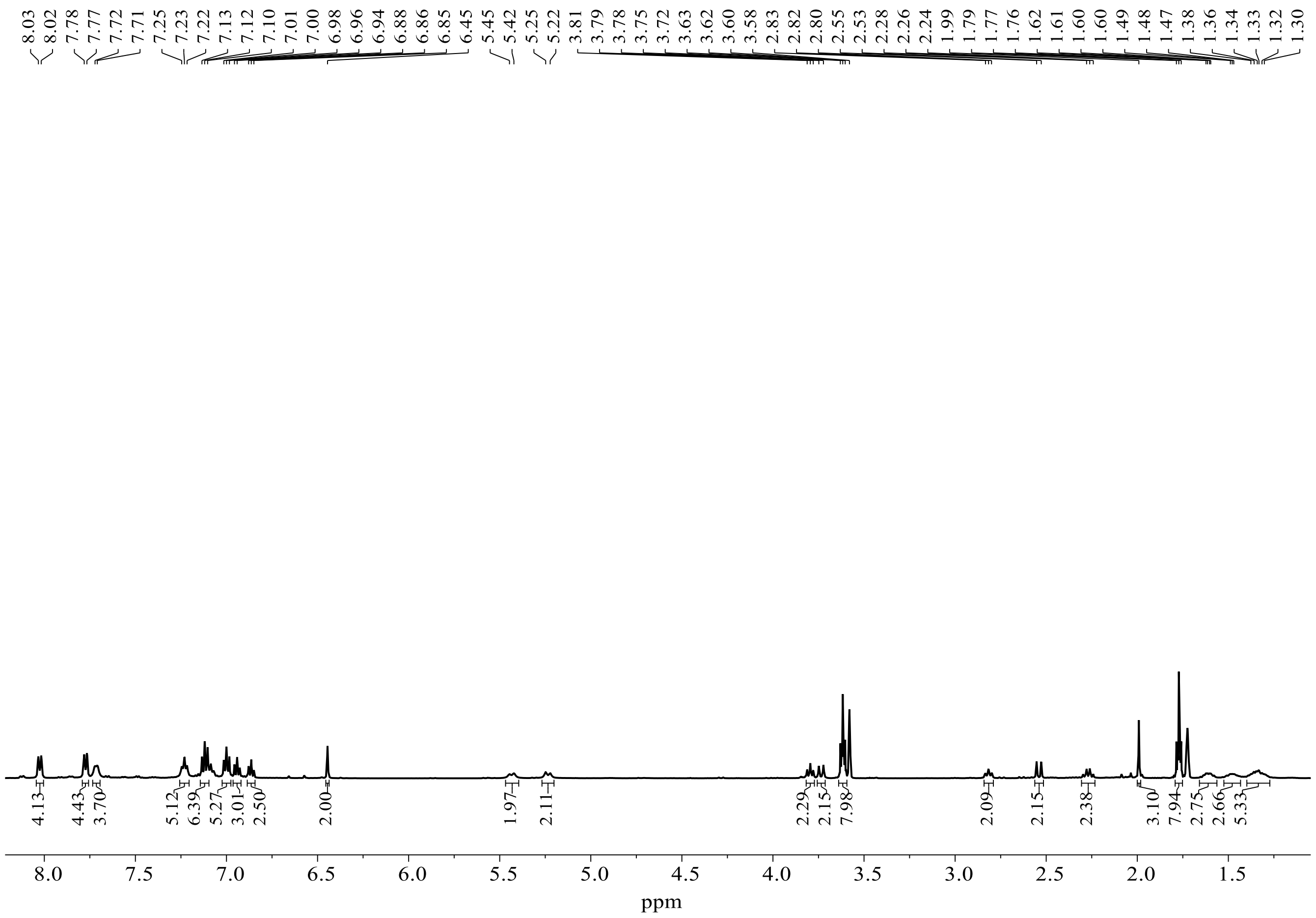

Figure S44. ^{1}H NMR spectrum of *in situ* generated complex **5** in THF-d_8 (298 K).

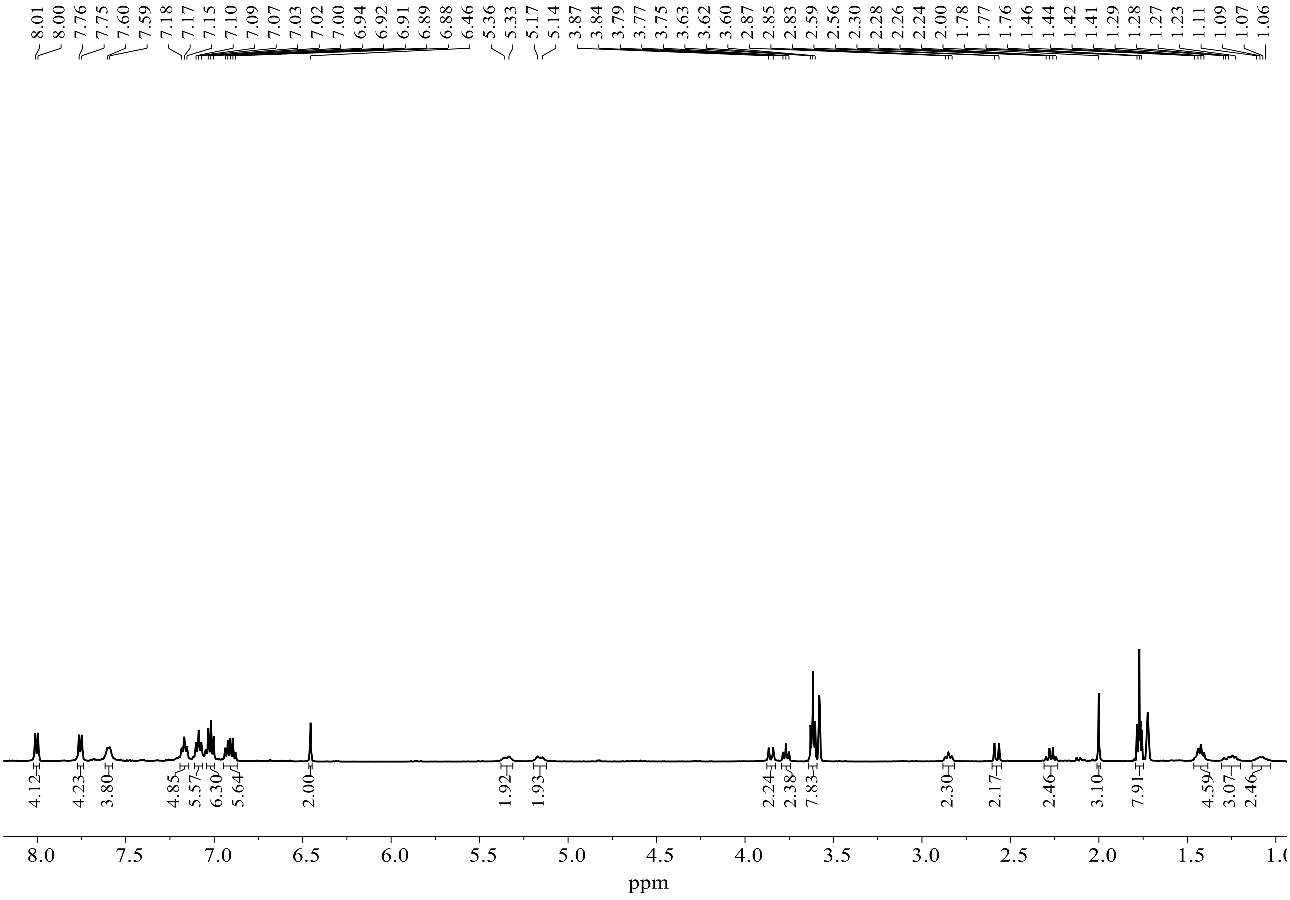

Figure S45. ^{1}H NMR spectrum of *in situ* generated complex **6** in THF-d_8 (298 K).

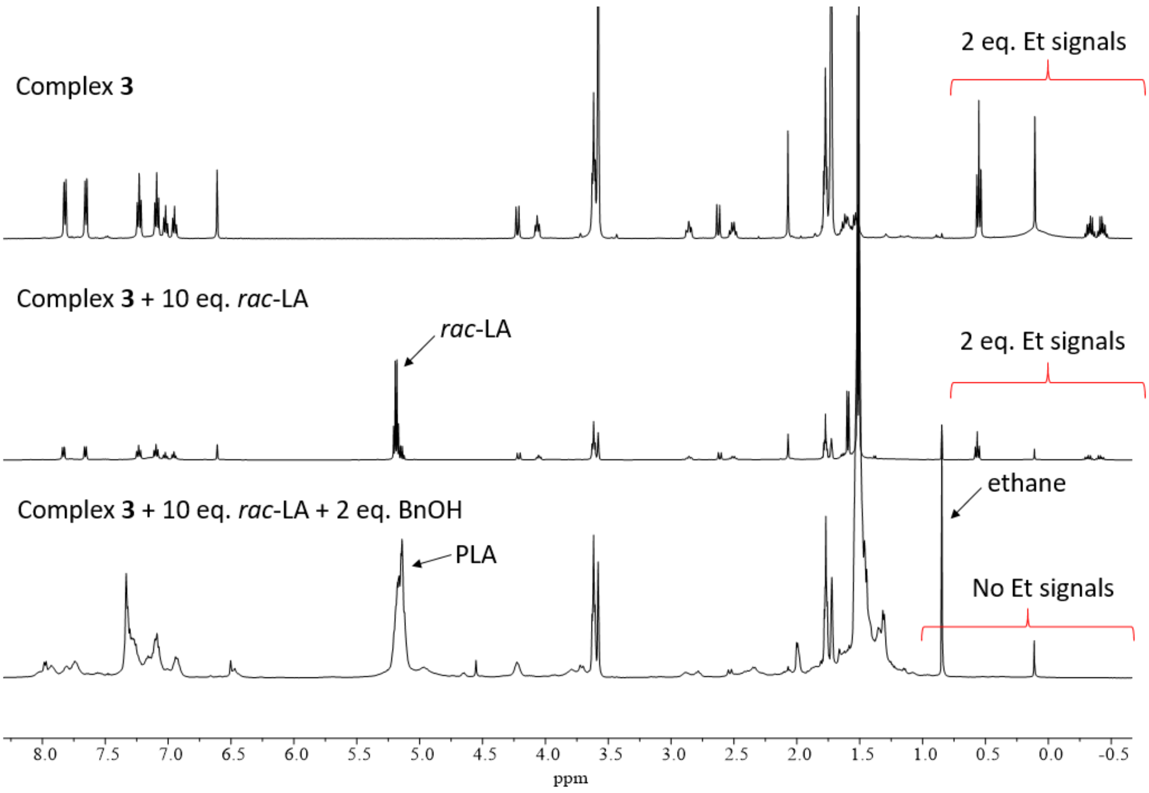

Figure S46. An overlay of ^{1}H NMR spectra of complex **3** in the presence and absence of 10 eq. *rac*-LA and 2 eq. BnOH in THF-d_8 (298 K).

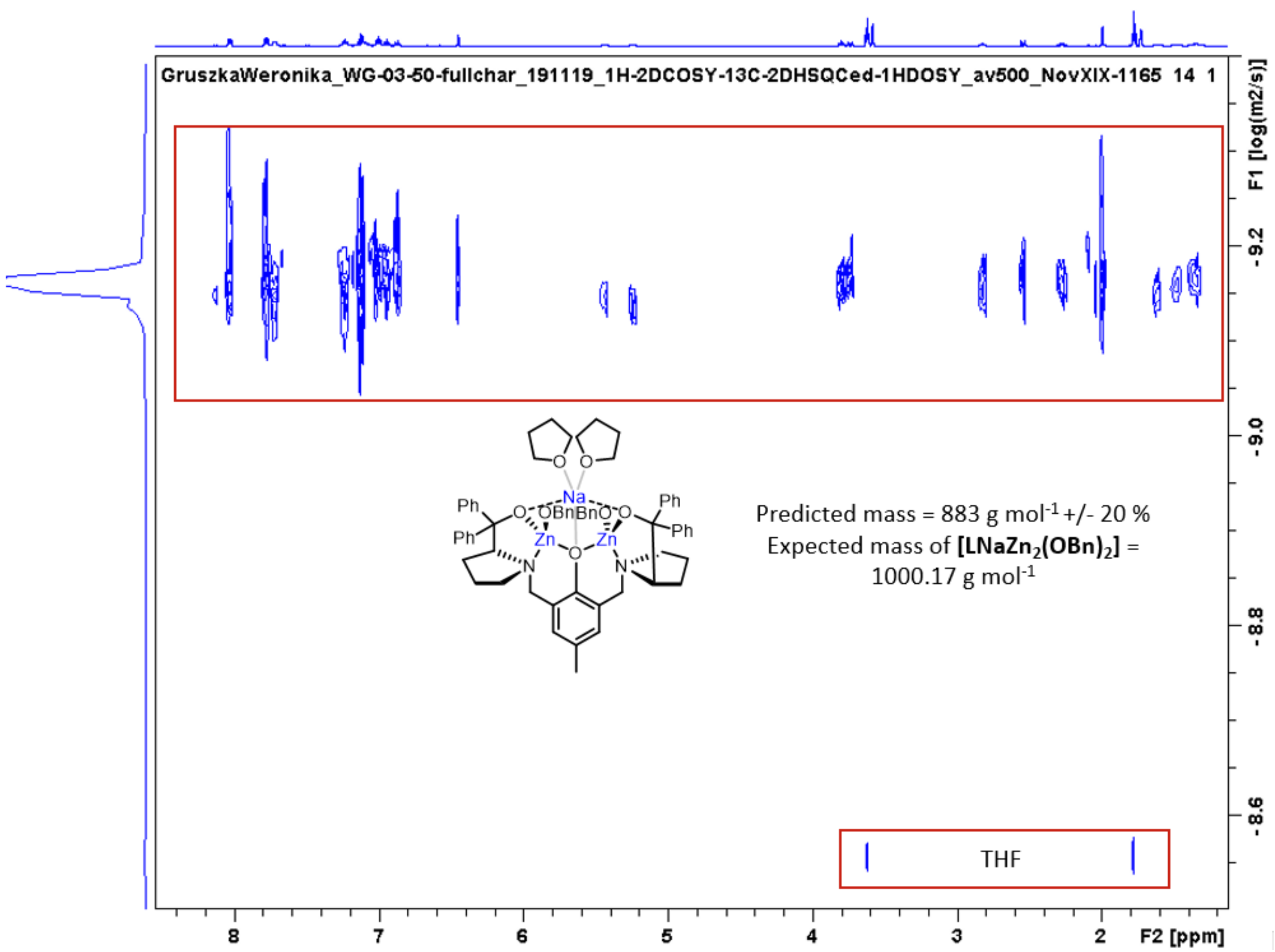

Figure S47. DOSY NMR spectrum of *in situ* generated complex **5** in THF-d_8 (298 K).

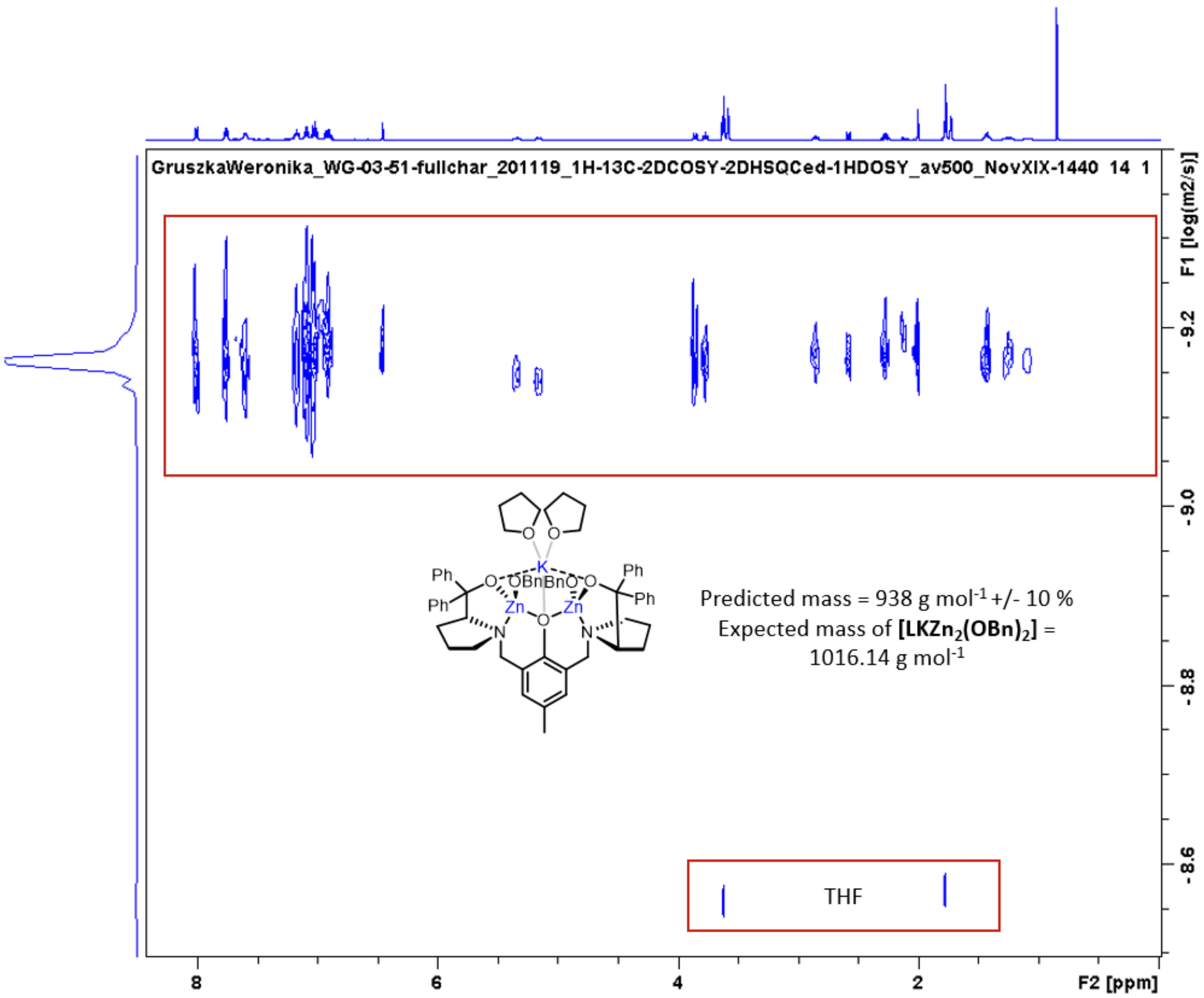

Figure S48. DOSY NMR spectrum of *in situ* generated complex **6** in THF-d_8 (298 K).

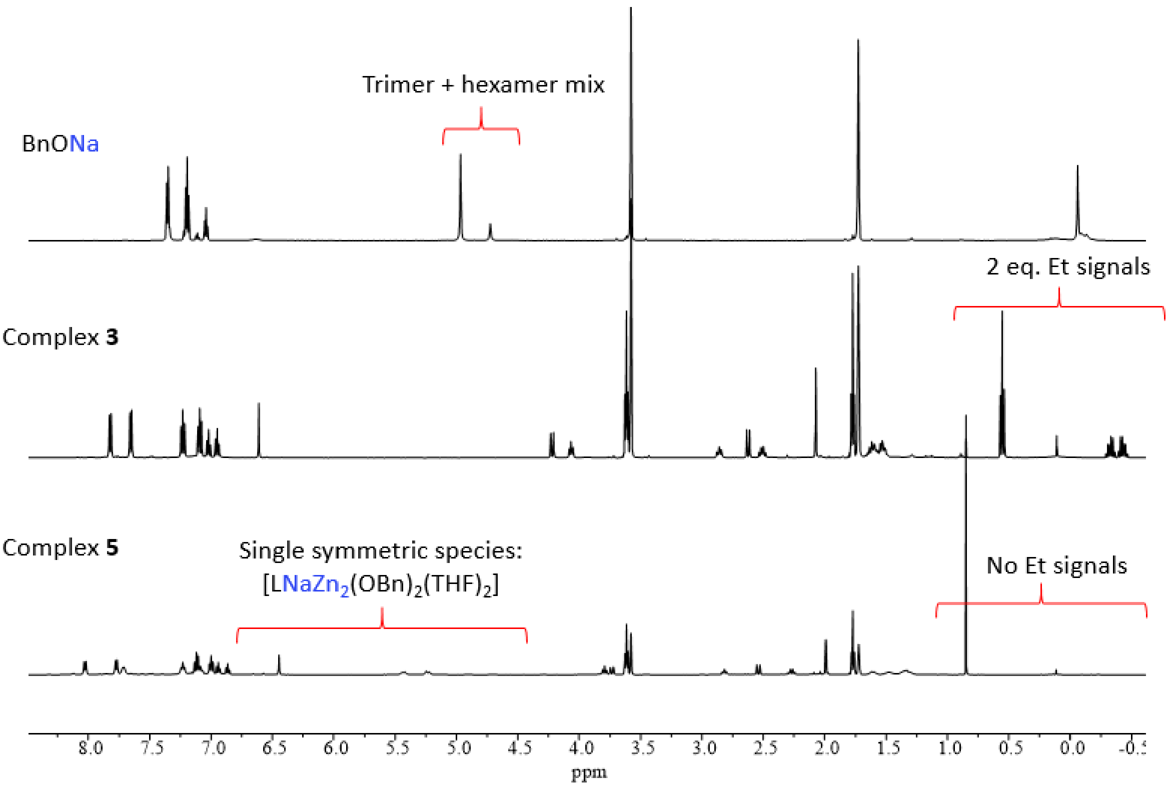

Figure S49. An overlay of ^{1}H NMR spectra of complexes **3**, *in situ* generated **5** and **[BnONa]** in THF-d_8 (298 K).

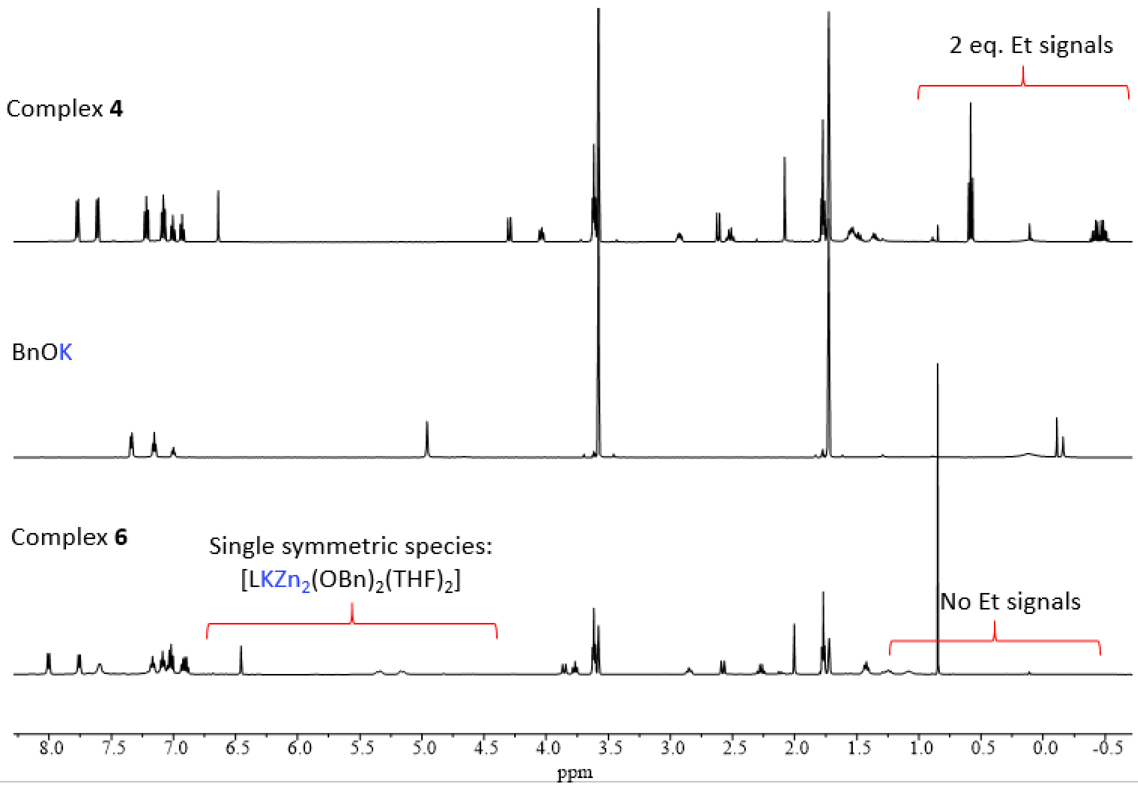

Figure S50. An overlay of ^{1}H NMR spectra of complexes **4**, *in situ* generated **6** and **[BnOK]** in THF-d_8 (298 K).

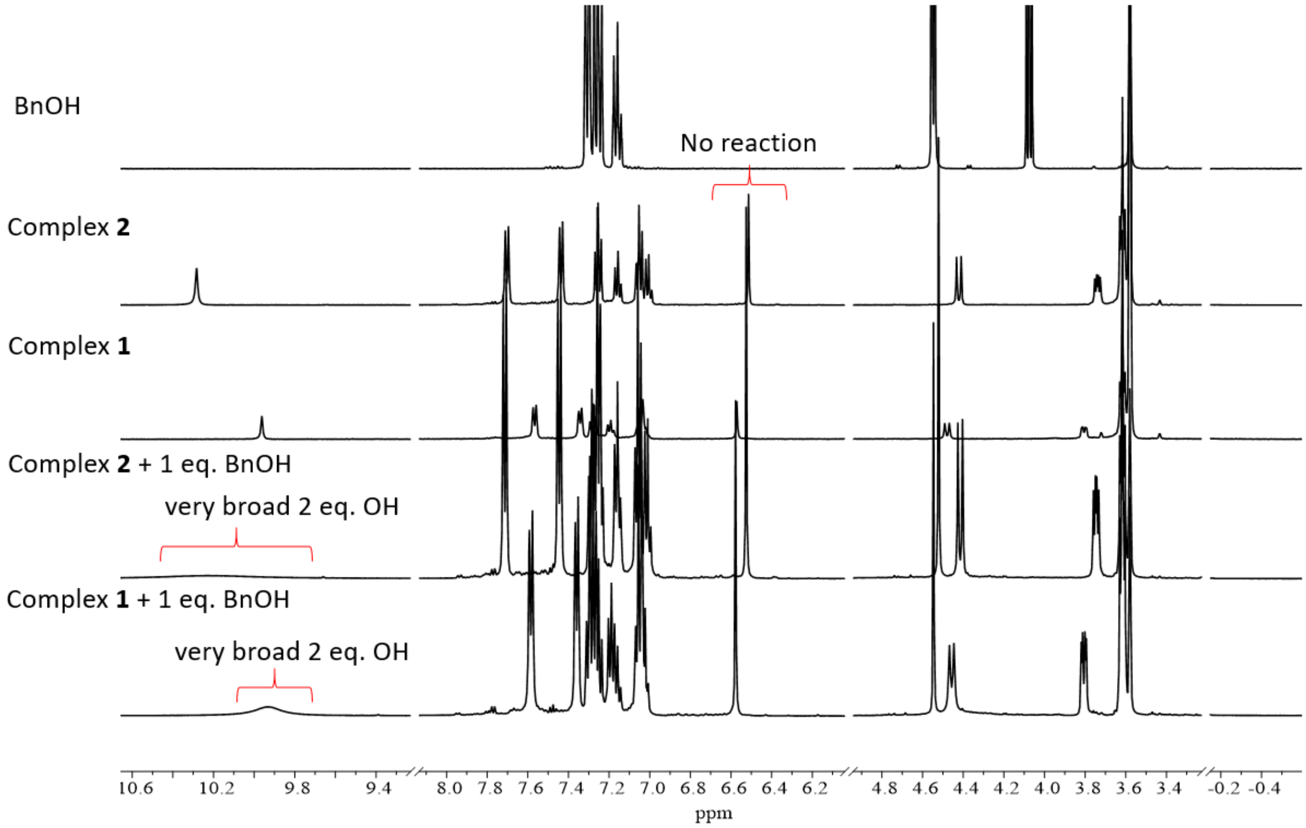

Figure S51. An overlay of ^{1}H NMR spectra of complexes **1** and **2** in the presence and absence of 1 eq. BnOH in THF-d_8 (298 K). No resonances for **[BnONa]** or **[BnOK]** were observed, suggesting that no reaction occurs between BnOH and **1** or **2**. However, Lewis coordination of BnOH may occur, as indicated by a minor shift of the PhCH_2OH resonance (from 4.55 ppm (reference BnOH) to 4.51 ppm (BnOH and complex **2**)) and a change in the PhCH_2OH splitting pattern (from a doublet (BnOH) to a singlet (in the presence of **1** or **2**)). Similarly, the benzylic OH signals of complexes **1** and **2** shift and broaden (9.93 and 10.24 ppm, respectively).

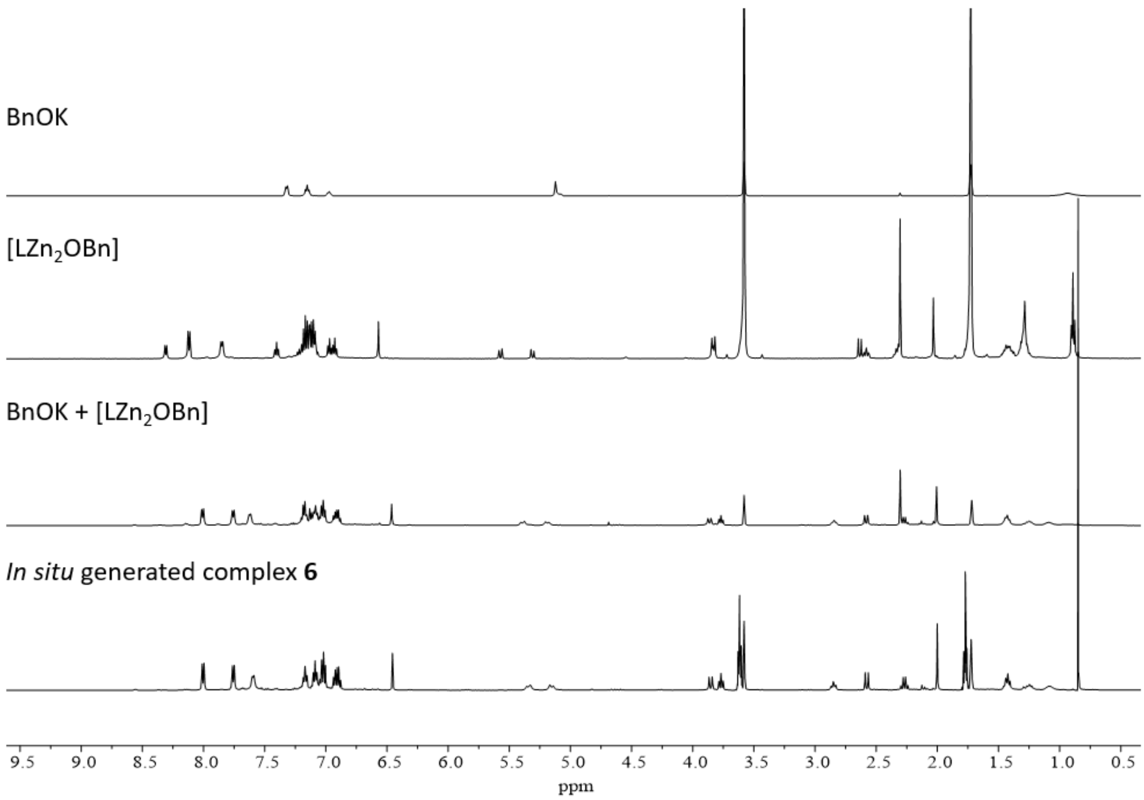

Figure S52. An overlay of ${}^{1}H$ NMR spectra of [BnOK], [LZn₂OBn], *in situ* generated complex **6** (from the reaction of **4** with 2 eq. BnOH) and product of the *in situ* reaction between [LZn₂OBn] and [BnOK] in THF-d_8 (298 K).

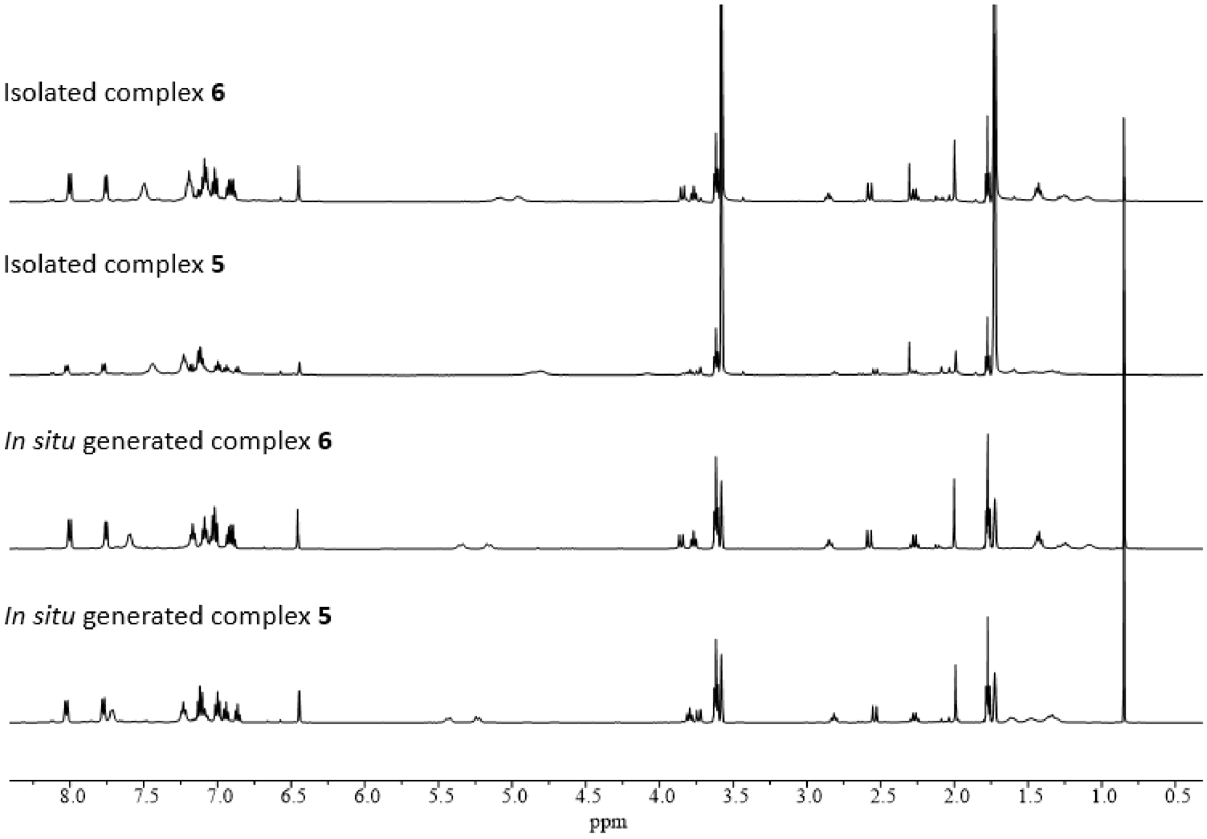

Figure S53. An overlay of ^{1}H NMR spectra of *in situ* generated and isolated complexes **5** and **6** in THF-d_8 (298 K).

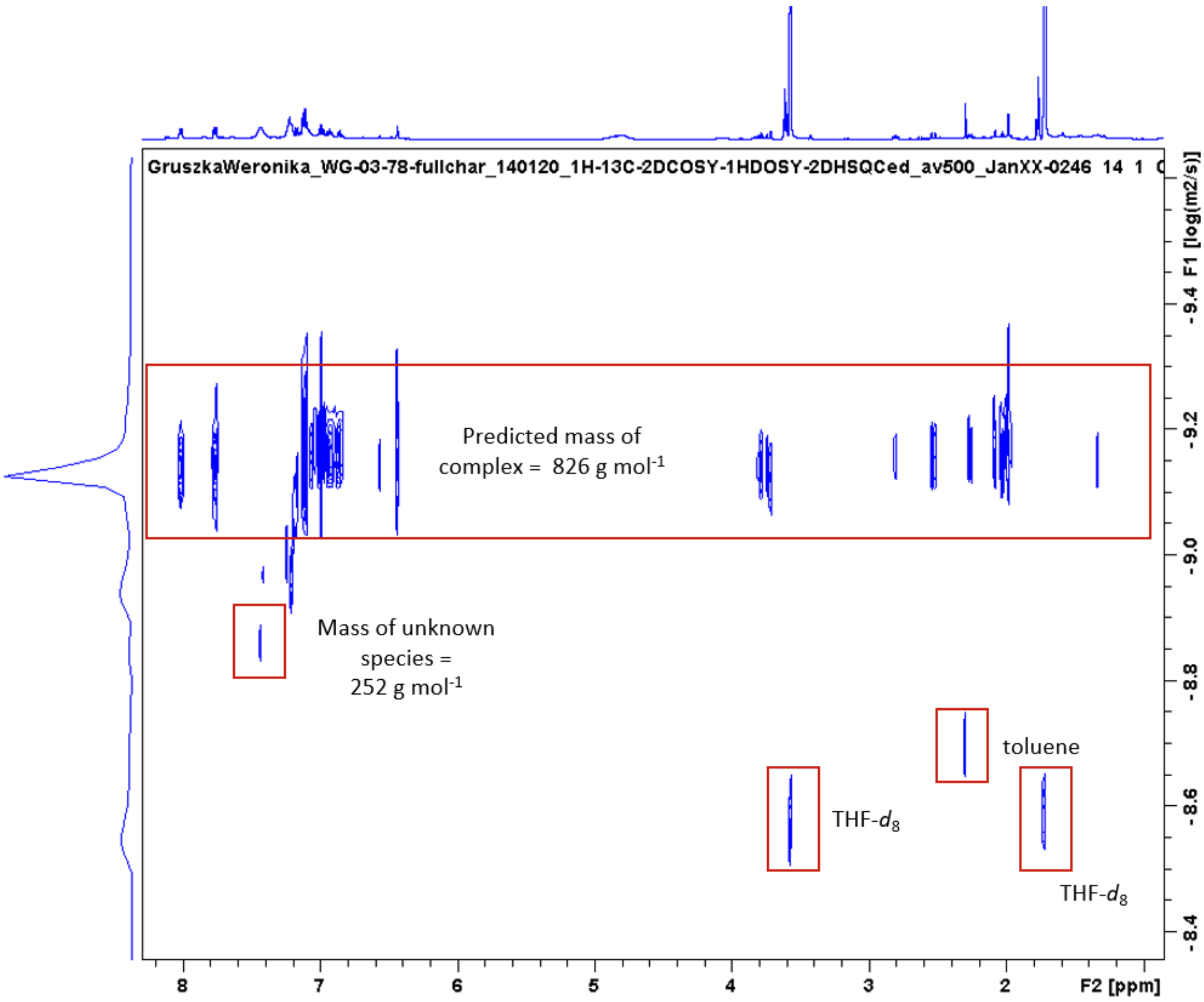

Figure S54. DOSY NMR spectrum of the 2-component mixture attained upon the attempted isolation of complex **5** in THF-d_8 (298 K).

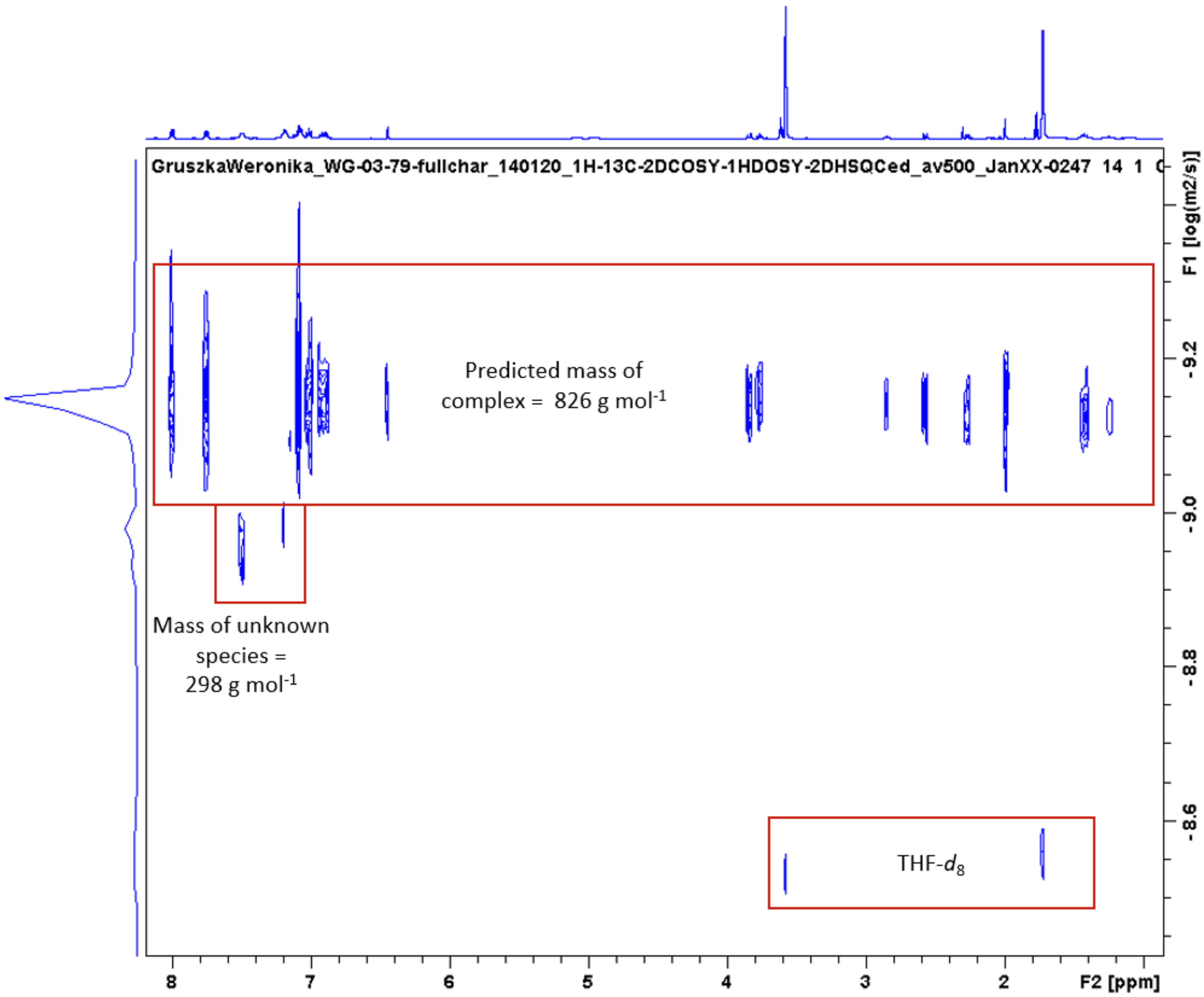

Figure S55. DOSY NMR spectrum of the 2-component mixture attained upon the attempted isolation of complex **6** in THF-d_8 (298 K).

Table S4. ROP of ε-CL and δ-VL with complexes **3** and **4** and 2 eq. of BnOH in THF at R.T.

Entry	Cat.	Monomer	Time (min)	Conv.[a] (%)	$M_{n,obs}$[b] (Da)	$M_{n,calc}$[c] (Da)	$Đ$[b]
**1**	**3**	ε-CL	0.08	53	5200	3000	1.52
**2**	**3**	ε-CL	0.33	83	4600	4700	1.62
**3**[d]	**3**	ε-CL	2	86	17200	24500	1.66
**4**[e]	**3**	ε-CL	4	76	19600	43400	1.36
**5**	**3**	δ-VL	0.08	99	10700[f]	5000	1.23
**6**	**4**	ε-CL	1	19	700	1100	1.18
**7**	**4**	ε-CL	2.5	20	800	1100	1.16
**8**	**4**	δ-VL	0.08	94	8600[f]	4700	1.21

100:1:2 monomer:catalyst:BnOH, [monomer] = 1 M in THF. [a] Conversion calculated using ^{1}H NMR spectroscopy. [b] $M_{n,obs}$ and $Đ$ determined by gel permeation chromatography using polystyrene standards in THF. Values corrected by Mark-Houwink factor (0.56). [c] $M_{n,calc}$ of polymers calculated from the monomer conversion $M_{n,calc}$ = M_0 x ([M]/[I]) x conversion assuming 2 chains per catalyst. [d] 500 eq. ε-CL. [e] 1000 eq. ε-CL. [f] $M_{n,obs}$ values reported are uncorrected.

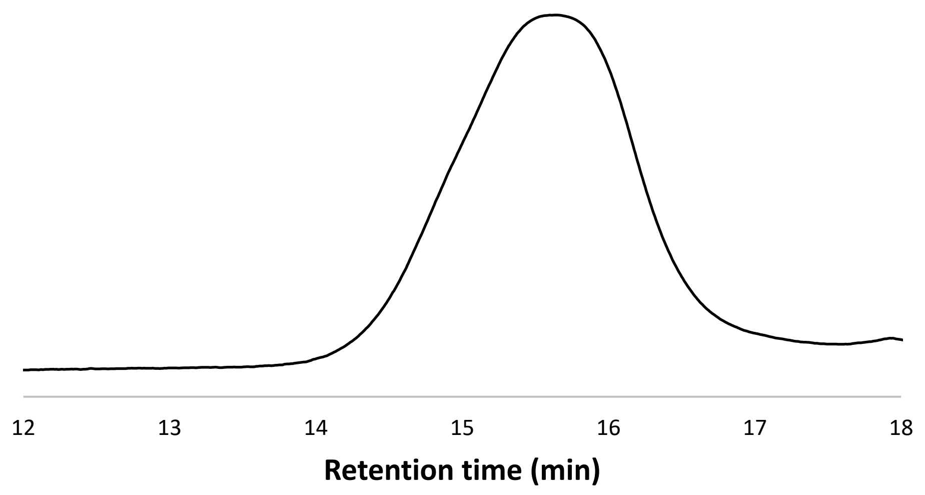

Figure S56. Example GPC trace of PCL generated in the presence of complex **3** + 2 eq. BnOH in THF at R.T. (Entry 2, Table S4).

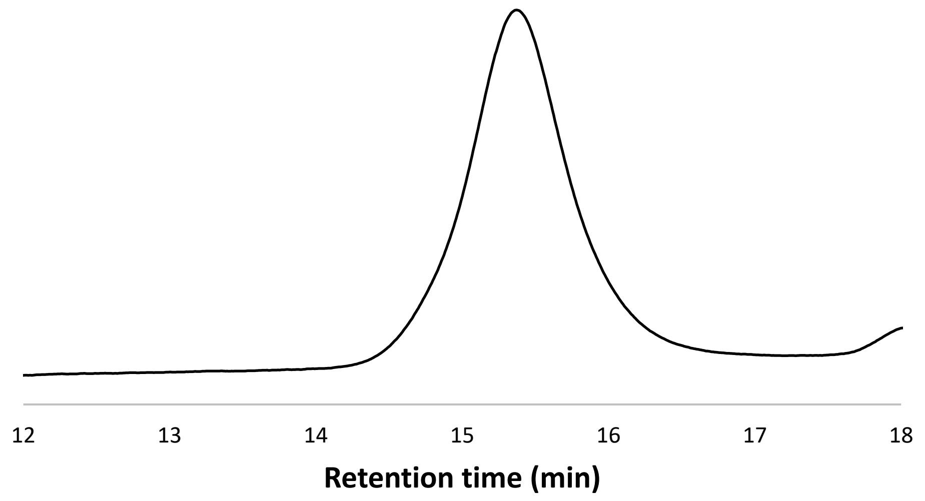

Figure S57. Example GPC trace of PVL generated in the presence of complex **3** + 2 eq. BnOH in THF at R.T. (Entry 5, Table S4).

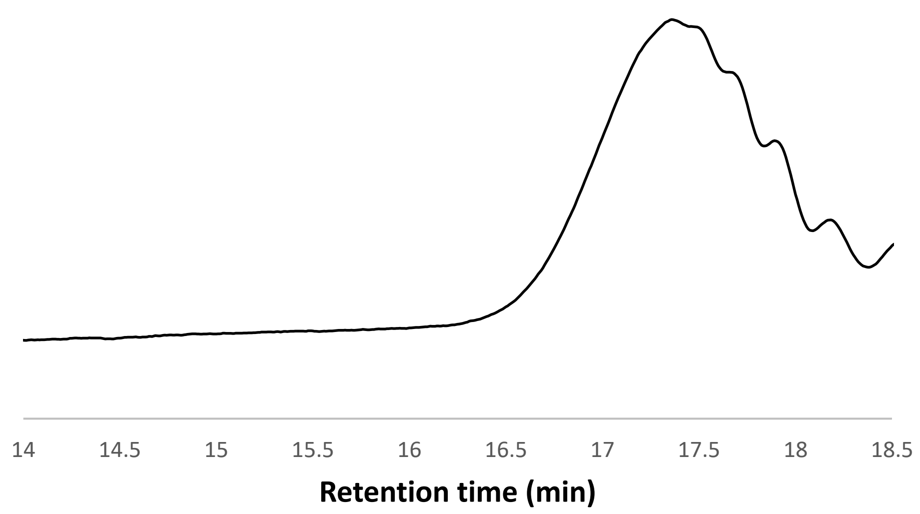

Figure S58. Example GPC trace of PCL generated in the presence of complex **4** + 2 eq. BnOH in THF at R.T. (Entry 6, Table S4).

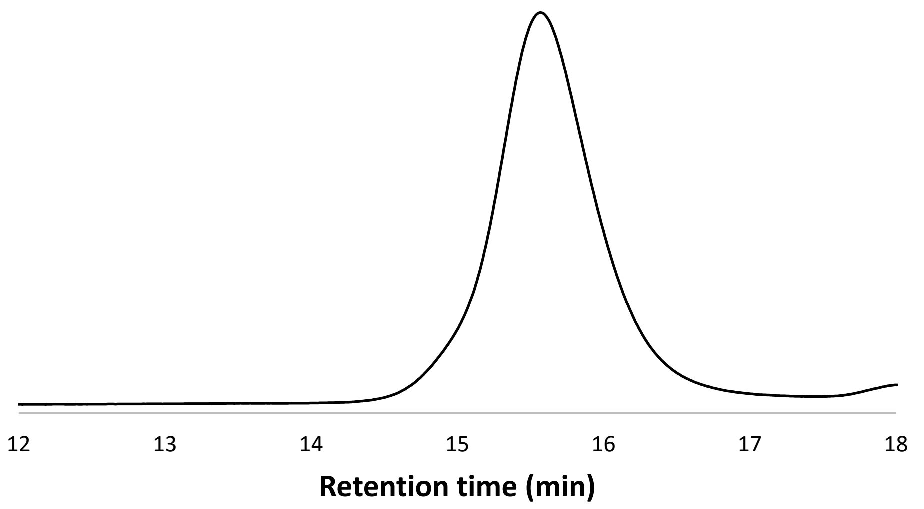

Figure S59. Example GPC trace of PVL generated in the presence of complex **4** + 2 eq. BnOH in THF at R.T. (Entry 8, Table S4).

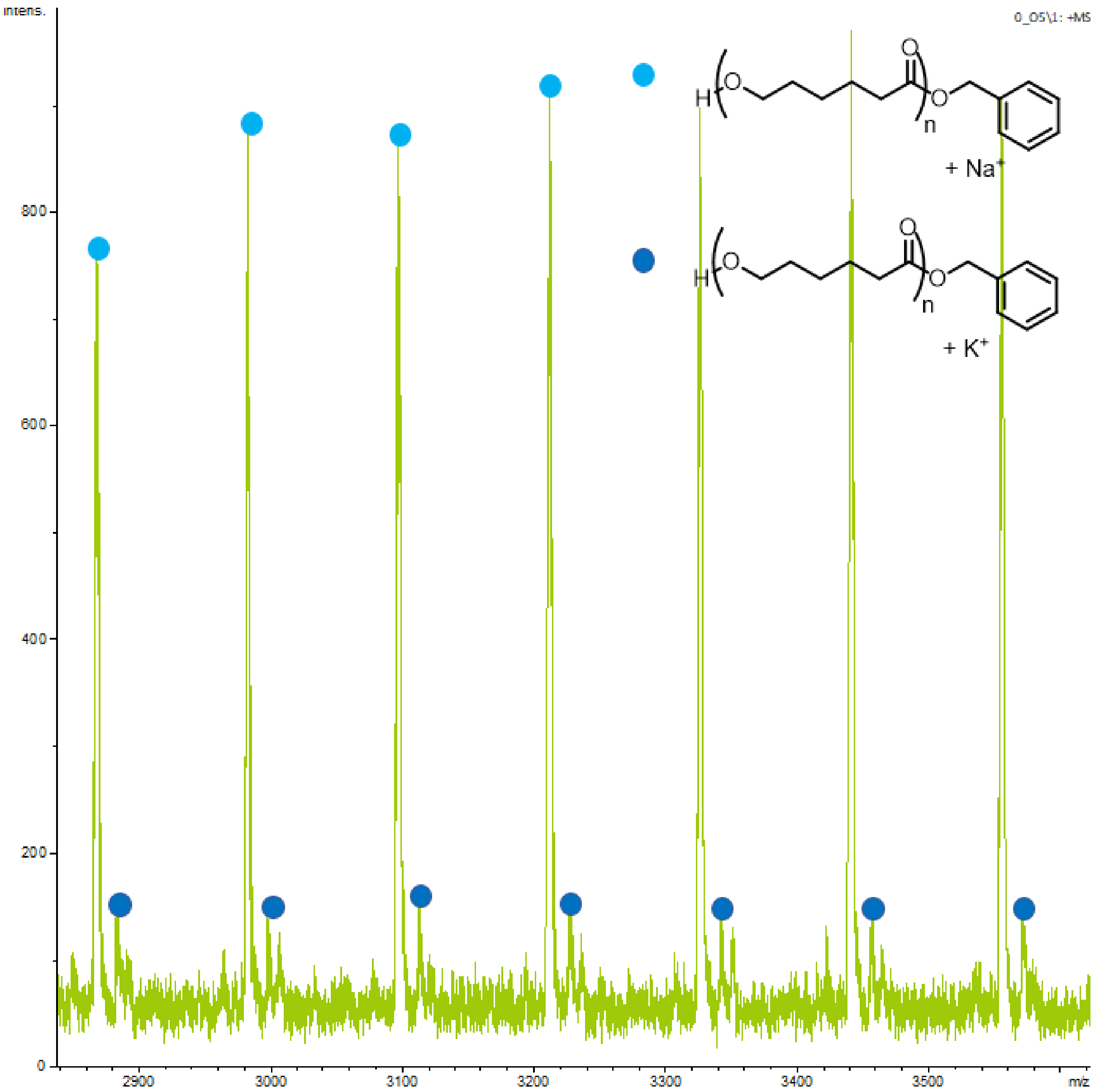

Figure S60. MALDI-TOF spectrum of PCL resulting from 53 % conversion of ε-CL in the presence of complex **3** + 2 eq. BnOH (THF, R.T.).

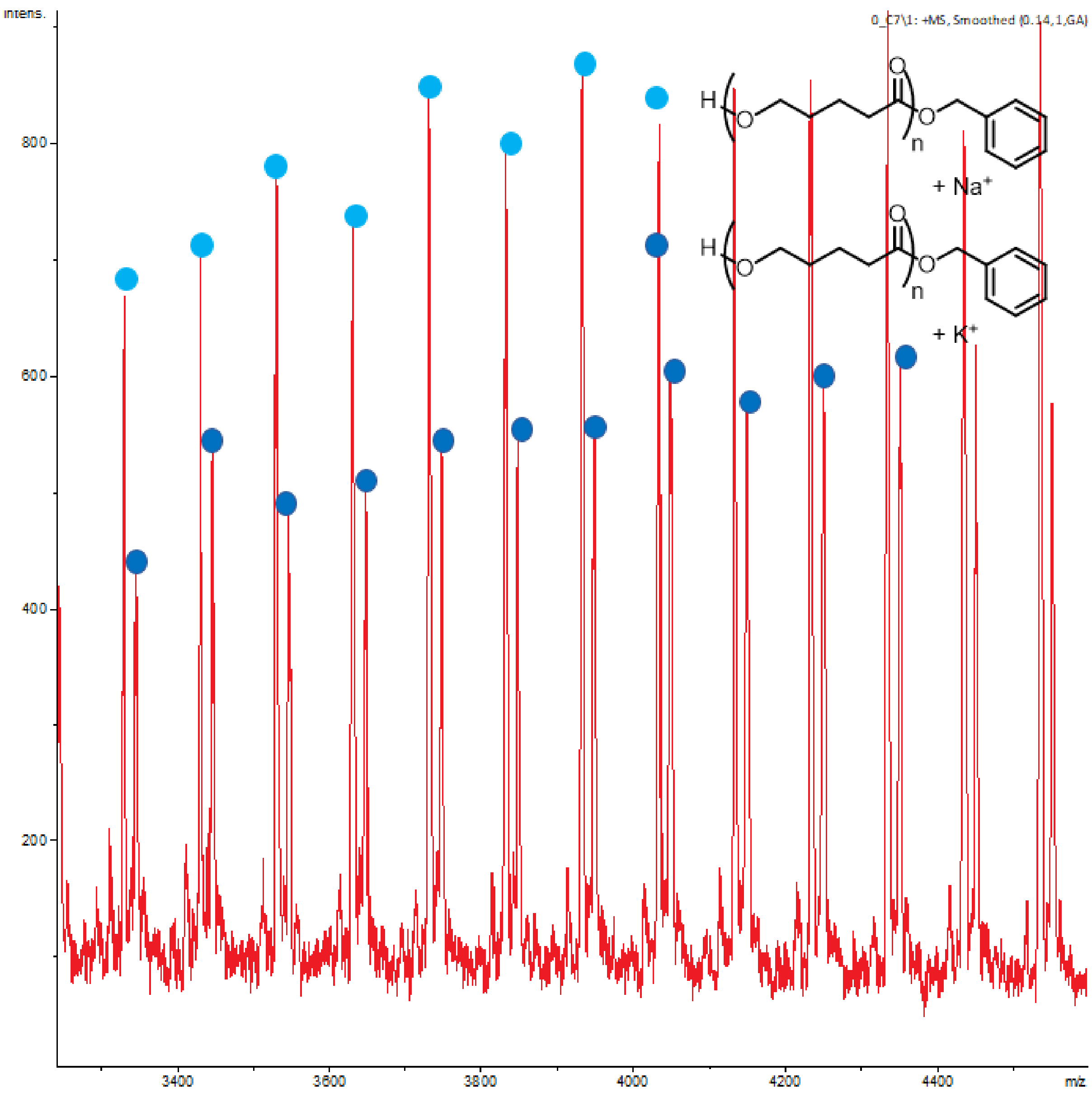

Figure S61. MALDI-TOF spectrum of PVL resulting from 99 % conversion of δ-VL in the presence of complex **3** + 2 eq. BnOH (THF, R.T.).

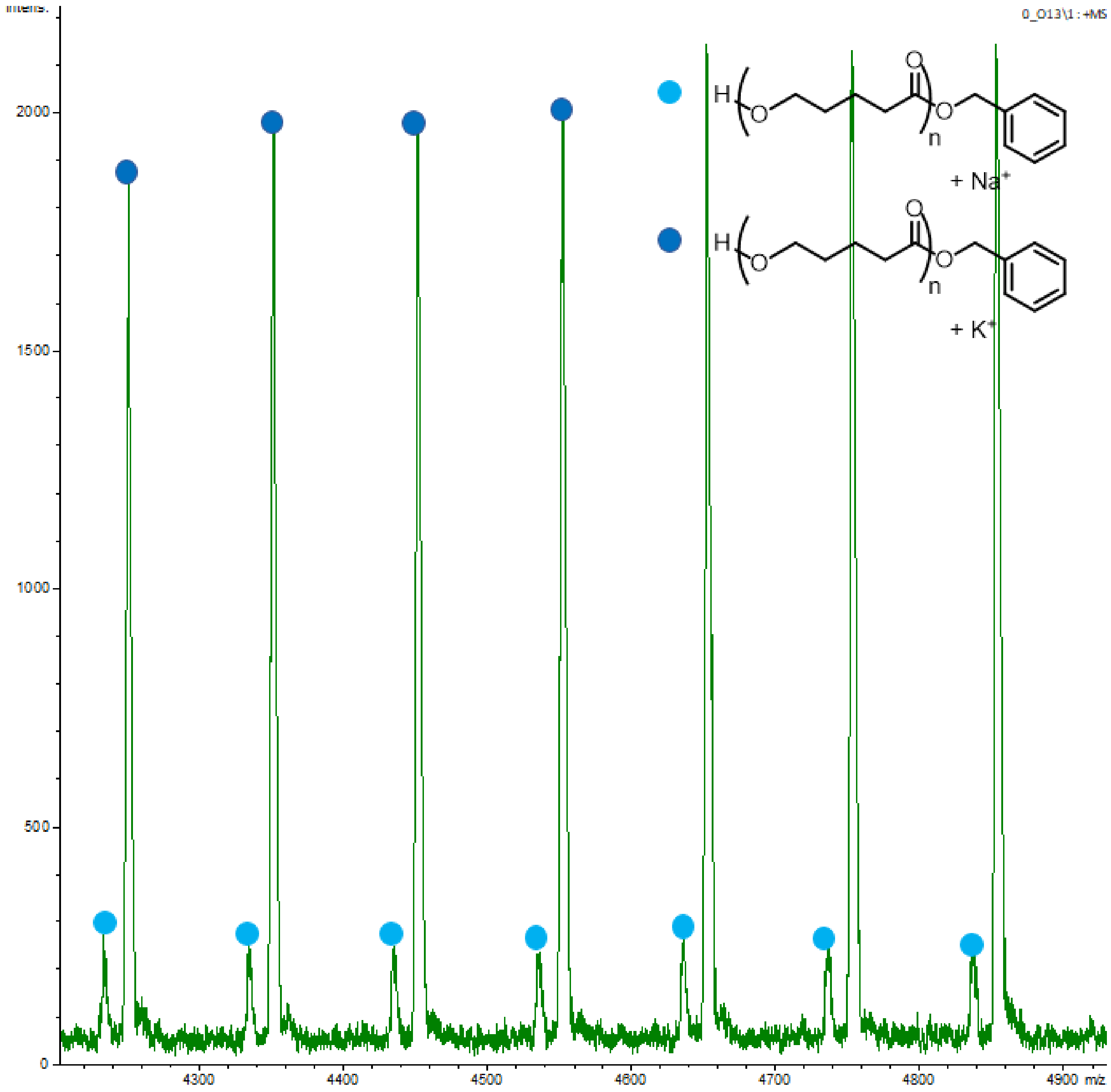

Figure S62. MALDI-TOF spectrum of PVL resulting from 94 % conversion of δ-VL in the presence of complex **4** + 2 eq. BnOH (THF, R.T.).

Table S5. Crystallographic data and collection and refinement details for complex **1** (CCDC reference number 2002496).

**Crystal data**	
Chemical formula	$C_{51}H_{61}N_2NaO_5 \cdot 2(C_4H_8O)$
M_r	949.21
Crystal system, space group	Tetragonal, $P4_3$
Temperature (K)	120
a, *c* (Å)	9.8142 (4), 54.0271 (12)
V (Å^3)	5203.8 (4)
Z	4
Radiation type	Cu $K\alpha$
μ (mm^{-1})	0.69
Crystal size (mm)	0.12 × 0.06 × 0.04
**Data collection**	
Diffractometer	Rigaku Oxford Diffraction SuperNova
Absorption correction	Multi-scan<br>*CrysAlis PRO* 1.171.40.61a (Rigaku Oxford Diffraction, 2019) Empirical absorption correction using spherical harmonics, implemented in SCALE3 ABSPACK scaling algorithm.
T_{min}, T_{max}	0.367, 1.000
No. of measured, independent and observed [$I > 2\sigma(I)$] reflections	10795, 10795, 7788
R_{int}	0.062
$(\sin \theta/\lambda)_{max}$ (Å^{-1})	0.602
**Refinement**	
$R[F^2 > 2\sigma(F^2)]$, $wR(F^2)$, S	0.069, 0.176, 0.97
No. of reflections	10795
No. of parameters	626
No. of restraints	71
H-atom treatment	H-atom parameters constrained
$\Delta\rangle_{max}$, $\Delta\rangle_{min}$ (e Å^{-3})	0.26, -0.26
Absolute structure	Classical Flack method preferred over Parsons because s.u. lower.
Absolute structure parameter	-0.06 (19)

Refinement special details: The O-bound H atoms were initially located from a difference Fourier map and controlled using the AFIX 147 restraint. The diffraction pattern was twinned. This was deconvoluted with CrysAlisPro.

Computed molecular structures of complex 3’

Potential molecular structures were optimised and their relative free enthalpies were compared. In particular, the relative position of the Zn-Et groups, the stereochemistry at the pyrrolidine nitrogen atoms as well as the coordination of one or two THF molecules to the alkali metal was investigated.

Table S6. Free enthalpies computed for various conformers of complex **3’** at the rωB97XD/6-311++g(d,p)/6-31g(d)/cpcm=tetrahydrofuran/298K level of theory.

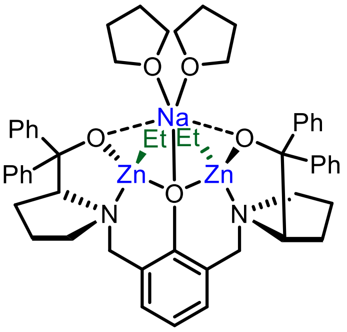

3'-1

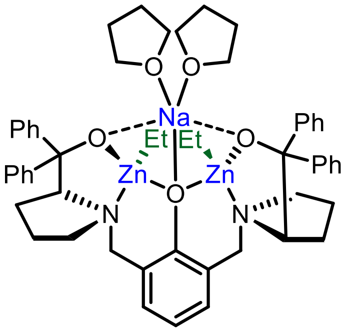

3'-2

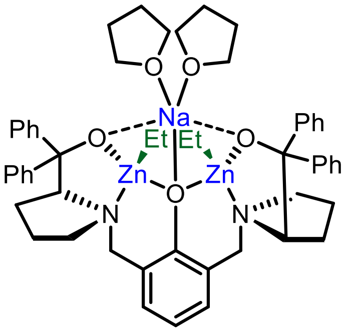

3'-3

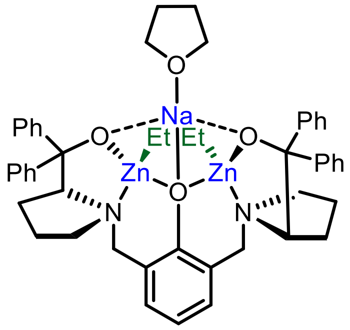

3'-4

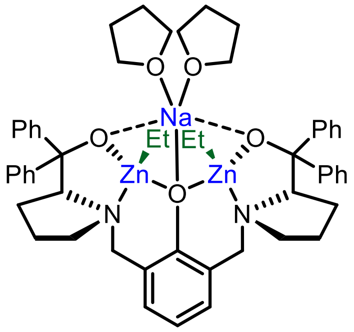

3'-5

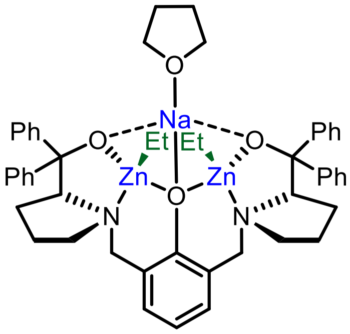

3'-6

Structure	Stereochemistry at the nitrogen atoms	THF coordinated to Na	Position of Zn-Et groups relative to phenol ring plane	G (Hartree)	ΔG (kcal mol^{-1})
**3’-1**	*R,R*	2	Opposite	–6303.269108	+0.0 (reference)
**3’-2**	*R,R*	2	Opposite	–6303.209369	+37.5
**3’-3**	*R,R*	2	Same	–6303.239257	+18.7
**3’-4**	*R,R*	1	Opposite	–6070.959147	
**THF**				–232.315175	
**3’-4 + THF**				–6303.274322	–3.3
**3’-5**	*R,S*	2	Same	–6303.265998	+2.0
**3’-6**	*R,S*	1	Same	–6070.941517	
**3’-6 + THF**				–6303.256692	+7.8

According to the calculations detailed in Table S6, a few structures (**3'-1**, **3'-4** and **3'-5**) are within 3 kcal mol^{-1} of each other and therefore represent plausible structures for complex **3** in solution. The *R*,*R* configuration of the ligand at the nitrogen atoms, as seen in the solid-state for **1**, is likely to be retained upon coordination of two ZnEt groups with the coordination of one THF molecule favoured slightly over the coordination of two THF molecules (–3.3 kcal mol^{-1}). However, in large excess of THF (solvent), this equilibrium may be shifted towards the coordination of two THF molecules, as seen experimentally. It is also plausible that the ligand may rearrange to a *meso* configuration (*R*,*S*) with Zn-Et groups facing in the same direction relative to the phenol ring (**3'-5**) in solution, despite being slightly endergonic (+2.0 kcal mol^{-1}). The molecular structure of **3'-4** with the lowest free enthalpy computed is shown in Figure S63.

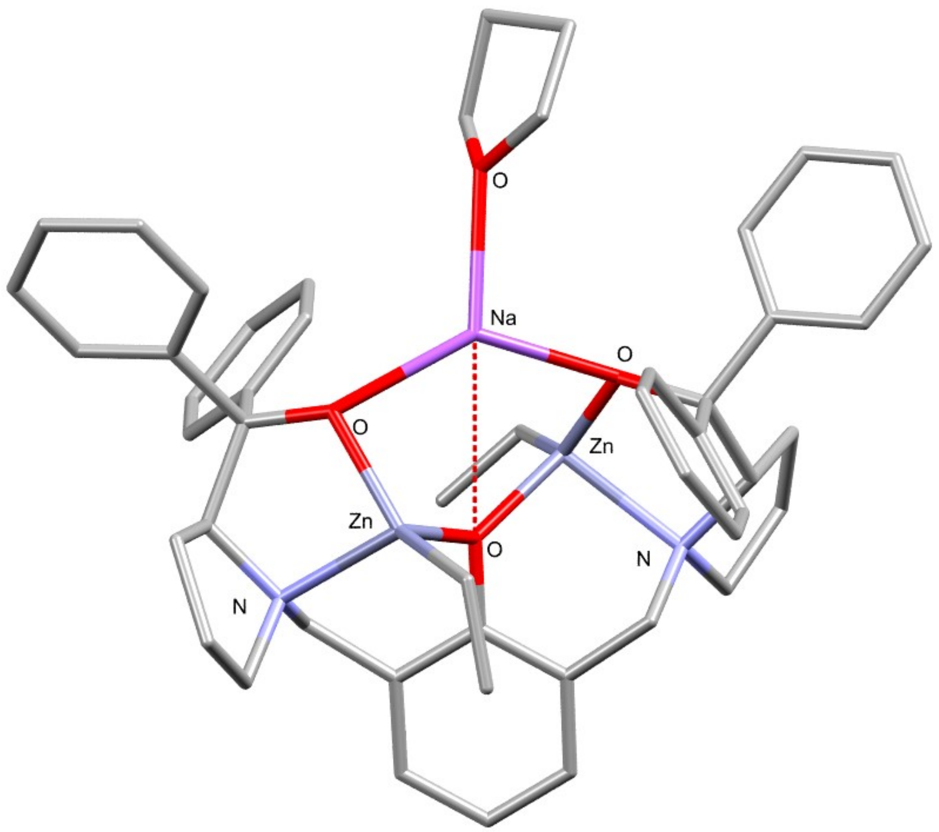

Figure S63. Molecular structure of complex **3'-4**, computed at the rωB97XD/6-311++g(d,p)/6-31g(d)/cpcm=tetrahydrofuran/298K level of theory.

Thermodynamics of reaction of complex 3’ with BnOH

Protonation reaction with 1 eq. BnOH

The thermodynamics of the reaction between **3’** and 1 eq. BnOH were evaluated starting from the two lowest free enthalpy structures for **3’**: **3’-1** (as observed experimentally) and **3’-5**, with *R*,*R* and *R*,*S* ligand configurations at the pyrrolidine nitrogen atoms, respectively.

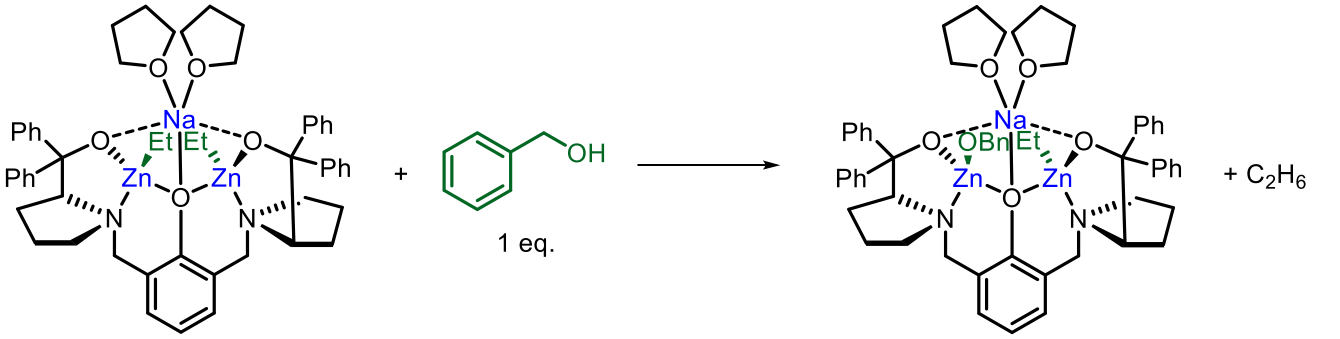

3'-1 **3'-1***
ΔG = -30.5 kcal mol^{-1}

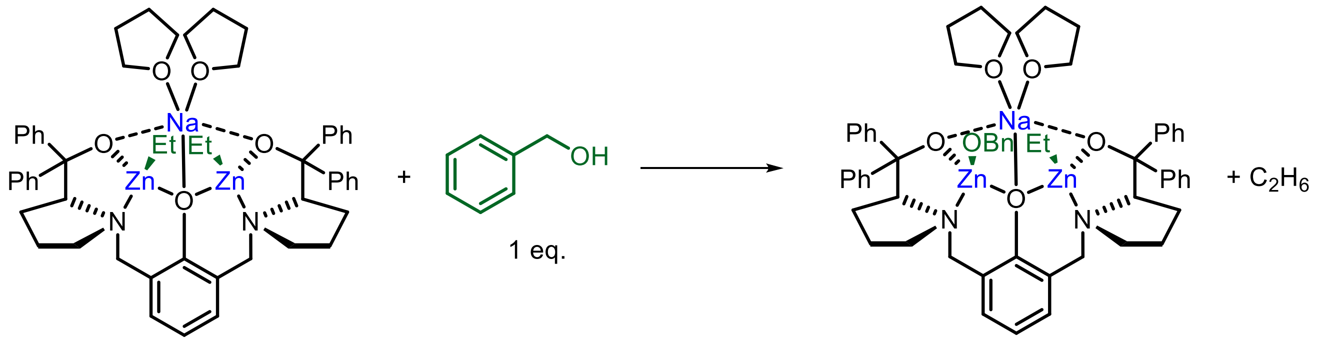

3'-5 **3'-5***
ΔG = -26.9 kcal mol^{-1}

Figure S64. Thermodynamics of protonation of complex **3’** with 1 eq. BnOH, calculated at the rωB97XD/6-311++g(d,p)/6-31g(d)/cpcm=tetrahydrofuran/298K level of theory.

Table S7. Free enthalpies computed for various conformers of complex **3’-n*** at the rωB97XD/6-311++g(d,p)/6-31g(d)/cpcm=tetrahydrofuran/298K level of theory.

Structure	Stereochemistry at the nitrogen atoms	THF coordinated to Na	Position of Zn-Et/OBn groups relative to phenol ring plane	G (Hartree)	ΔG (kcal mol^{-1})
**BnOH**				–346.578891	
**Et**				–79.748587	
**3’-1**	*R,R*	2	Opposite	–6303.269108	+0.0 (reference)
**3’-1***	*R,R*	2	Opposite	–6570.148038	
**3’-1* + Et – BnOH**				–6303.317734	–30.5
**3’-5**	*R,S*	2	Same	–6303.265998	+0.0 (reference)
**3’-5***	*R,S*	2	Same	–6570.139199	
**3’-5* + Et – BnOH**				–6303.308895	–26.9

From the calculations highlighted in Table S7, the protonation of one Zn-Et group with 1 eq. BnOH in **3’-1** and **3’-5** to form a Zn-OBn moiety is thermodynamically favoured, regardless of the geometry of the mixed Et/OBn complex.

Thermodynamics of protonation reaction of 3’ with 2 eq. BnOH: formation of bis-OBn complex 5’

The thermodynamics of the reaction between **3’** and 2 eq. BnOH were evaluated starting from two lowest free enthalpy structures for **3’**: **3’-1** (as observed experimentally) and **3’-5**, with *R*,*R* and *R*,*S* ligand configuration at the pyrrolidine nitrogen atoms, respectively. The geometry of the resulting complex was not changed.

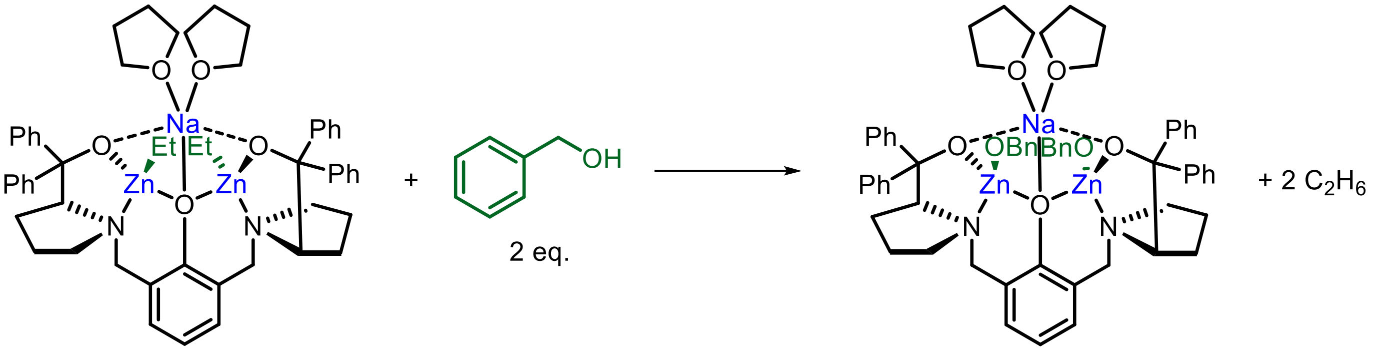

3'-1

5'-1

ΔG = -56.9 kcal mol^{-1}

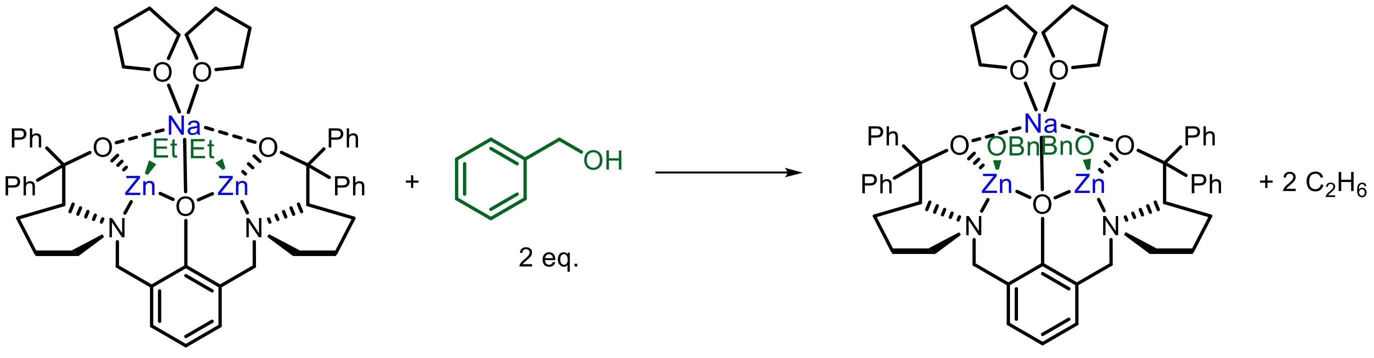

3'-5

5'-5

ΔG = -51.9 kcal mol^{-1}

Figure S65. Thermodynamics of protonation of complex 3’ with 2 eq. BnOH, calculated at the rωB97XD/6-311++g(d,p)/6-31g(d)/cpcm=tetrahydrofuran/298K level of theory.

Table S8. Free enthalpies computed for the protonation of complex **3'** into complex **5'** at the rωB97XD/6-311++g(d,p)/6-31g(d)/cpcm=tetrahydrofuran/298K level of theory.

Structure	Stereochemistry at the nitrogen atoms	THF coordinated to Na	Position of Zn-OBn groups relative to phenol ring plane	G (Hartree)	ΔG (kcal mol^{-1})
**BnOH**				–346.578891	
**Et**				–79.748587	
**3'-1**	*R,R*	2	Opposite	–6303.269108	+0.0 (reference)
**5'-1**	*R,R*	2	Opposite	–6837.020241	
**5'-1 + 2 Et – 2 BnOH**				–6303.359633	–56.9
**3'-5**	*R,S*	2	Same	–6303.265998	+0.0 (reference)
**5'-5**	*R,S*	2	Same	–6837.009322	
**5'-5 + 2 Et – 2 BnOH**				–6303.348714	–51.9

From the calculations highlighted in Table S8, the protonation of two Zn-Et groups with 2 eq. BnOH in **3'-1** and **3'-5** to form a bis Zn-OBn complex **5'** is thermodynamically favoured, regardless of the geometry of the mixed Et/OBn complex.

<u>**Computed molecular structures of complex 5'**</u>

Several possible molecular structures of complex **5'** were optimised and their relative free enthalpies were compared. In particular, the relative position of the Zn-OBn groups, the stereochemistry at the pyrrolidine nitrogen atoms as well as the coordination of one or two THF molecules to the alkali metal was investigated.

Table S9. Free enthalpies computed for various conformers of complex **5’** at the rωB97XD/6-311++g(d,p)/6-31g(d)/cpcm=tetrahydrofuran/298K level of theory.

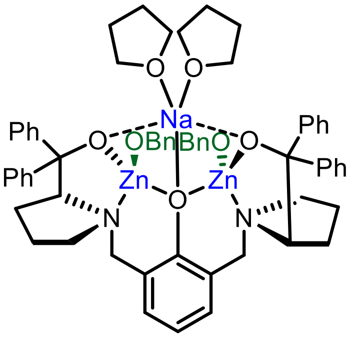

5'-1

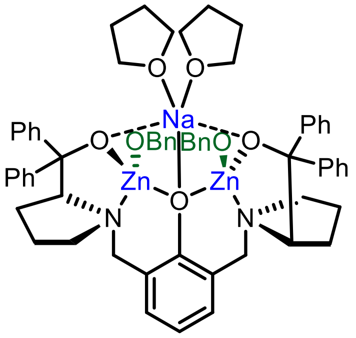

5'-2

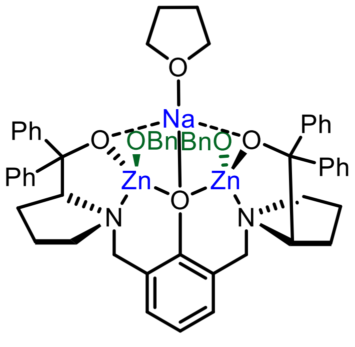

5'-4

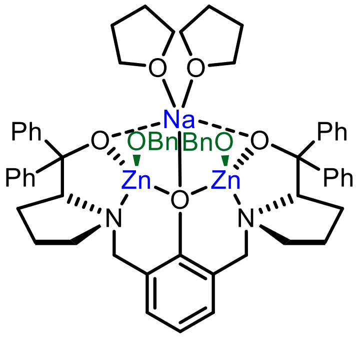

5'-5

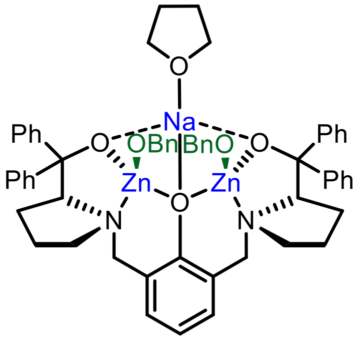

5'-6

Structure	Stereochemistry at the nitrogen atoms	THF coordinated to Na	Position of Zn-Et groups relative to phenol ring plane	G (Hartree)	ΔG (kcal mol^{-1})
**5’-1**	*R*,*R*	2	Opposite	−6837.020241	+0.0 (reference)
**5’-2**	*R*,*R*	2	Opposite	−6837.023146	-1.8
**5’-4**	*R*,*R*	1	Opposite	−6604.715559	
**THF**				−232.315175	
**5’-4 + THF**				−6837.030734	−6.7
**5’-5**	*R*,*S*	2	Same	−6837.009322	+6.9
**5’-6**	*R*,*S*	1	Same	−6604.703344	
**5’-6 + THF**				−6837.018519	+1.1

According to the calculations detailed in Table S9, structure **5’-4** has the lowest free enthalpy, and therefore represents a plausible structure for complex **5** in solution. The *R*,*R* configuration of the ligand at the pyrrolidine nitrogen atoms, as seen in the solid-state for **1**, is likely retained upon reaction of **3’** with 2 eq. BnOH, with the coordination of one THF molecule favoured over the coordination of two (–

6.7 kcal mol^{-1}). It is plausible that the rearrangement of the ligand to a *meso* configuration (*R*,*S*) with the two Zn-OBn groups facing in the same direction relative to the phenol ring and coordination of one THF molecule (structure **5’-6**), might also be accessible under the polymerisation conditions, as this structure is only endergonic by 7.8 kcal mol^{-1} compared to ground state **5’-4**. The molecular structure with the lowest free enthalpy computed, **5’-4**, is displayed in Figure S66.

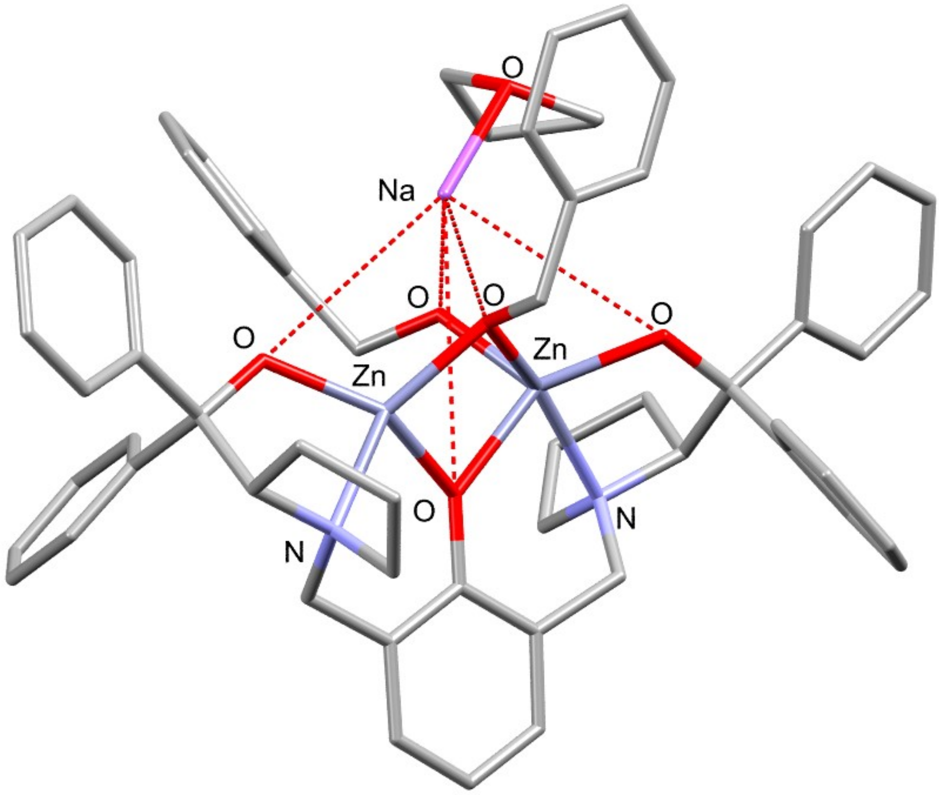

Figure S66. Molecular structure of complex **5’-4**, computed at the rωB97XD/6-311++g(d,p)/6-31g(d)/cpcm=tetrahydrofuran/298K level of theory.

__**Thermodynamics of possible rearrangement reactions of complex 5'**__

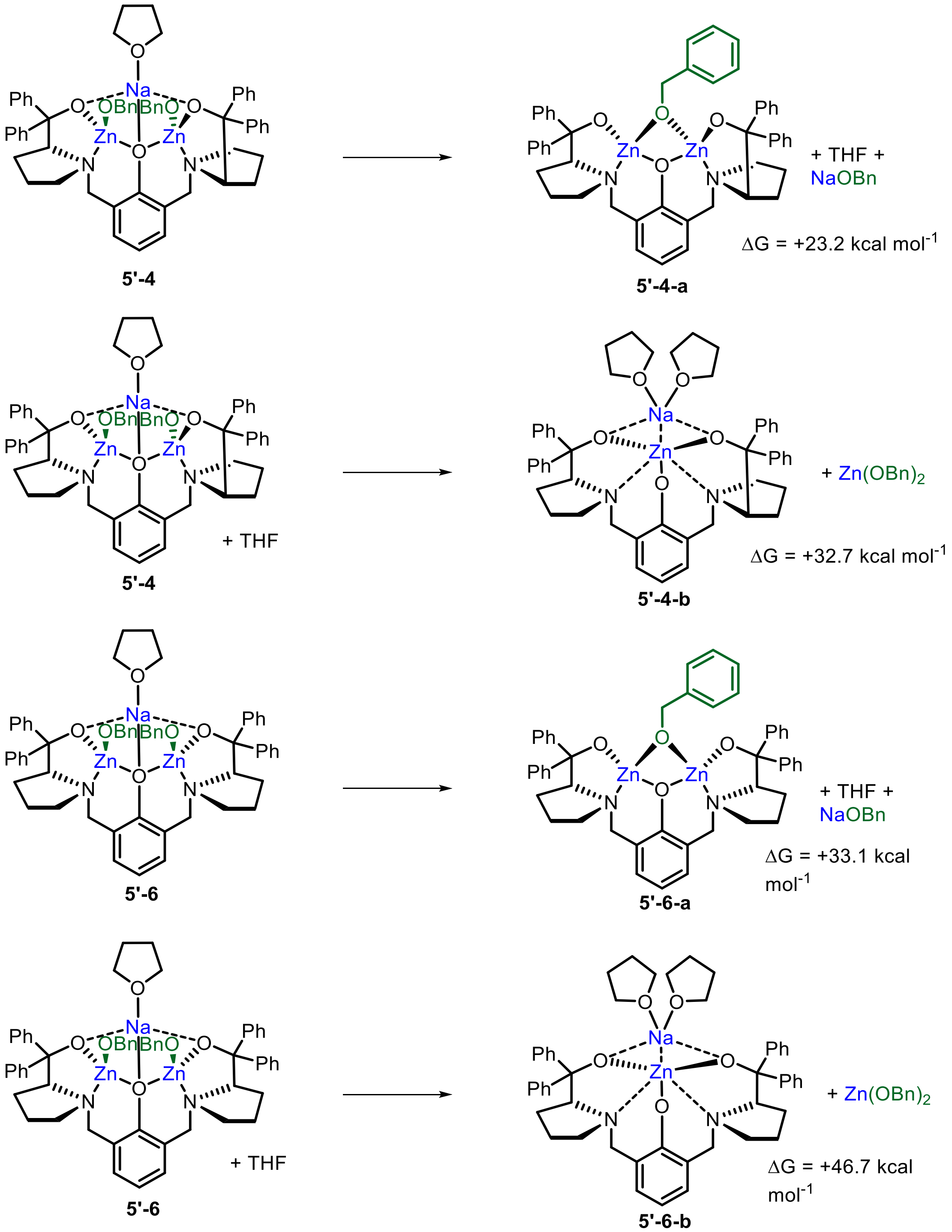

Figure S67. Thermodynamics of possible rearrangement reactions of complex **5'**, calculated at the rωB97XD/6-311++g(d,p)/6-31g(d)/cpcm=tetrahydrofuran/298K level of theory.

Table S10. Free enthalpies computed for various homo- and heterometallic rearrangements of complex 5’ at the rωB97XD/6-311++g(d,p)/6-31g(d)/cpcm=tetrahydrofuran/298K level of theory.

Structure	Stereochemistry at the nitrogen atoms	THF coordinated to Na	Position of Zn-OBn groups relative to phenol ring plane	G (Hartree)	ΔG (kcal mol^{-1})
**NaOBn**				–508.339091	
**Zn(OBn)$_2$**				–972.961064	
**THF**				–232.315175	
**5’-4**	*R,R*	1	Opposite	–6604.715559	+0.0 (reference)
**5’-4-a**	*R,R*	0	N/A	–5864.024329	
**5’-4-a + THF + NaOBn**				–6604.678595	+23.2
**5’-4**	*R,S*	1	Opposite	–6604.715559	+0.0 (reference)
**5’-4-b**	*R,S*	2	N/A	–4365.599346	
**5’-4-b + Zn(OBn)$_2$ – THF**				–6604.663347	+32.7
**5’-6**	*R,S*	1	Same	–6604.703344	+0.0 (reference)
**5’-6-a**	*R,S*	0	N/A	-5863.996266	
**5’-6-a + THF + NaOBn**				-6604.650532	+33.1
**5’-6**	*R,S*	1	Same	–6604.703344	+0.0 (reference)
**5’-6-b**	*R,S*	2	N/A	–4365.564954	
**5’-6-b + Zn(OBn)$_2$ – THF**				–6604.628955	+46.7

The calculations in Table S10 provide further evidence for the protonation of two Zn-Et groups in **3’** to form a bis Zn-OBn complex **5’** and suggest that the heterometallic complex **5** is thermodynamically favourable.

<u>Coordination of L-LA to complex 5’ and molecular structures of the resulting complexes</u>

Several possible molecular structures for **5’** with coordinated L-LA molecules were optimised and their relative free enthalpies were compared. In particular, the relative position of the Zn-OBn groups, the stereochemistry at the pyrrolidine nitrogen atoms as well as the coordination of one or two L-LA molecules to the alkali metal was investigated.

Table S11. Free enthalpies computed for various structures of complex **5’-n-LLA** at the rωB97XD/6-311++g(d,p)/6-31g(d)/cpcm=tetrahydrofuran/298K level of theory.

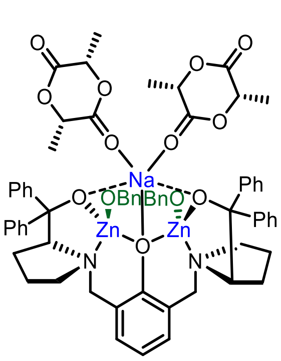

5'-1-LLA

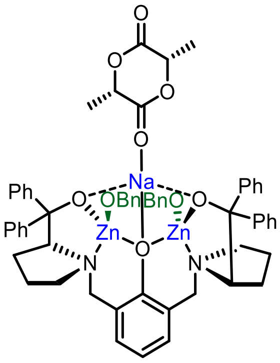

5'-4-LLA

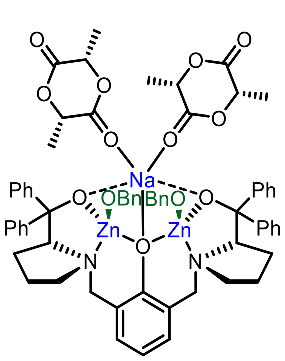

5'-5-LLA

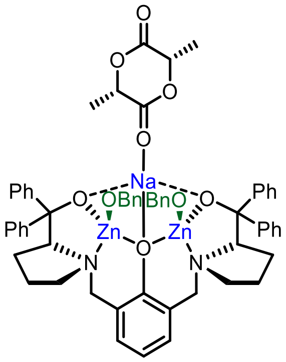

5'-6-LLA

Structure	Stereochemistry at the nitrogen atoms	THF or L-LA coordinated to Na	Position of Zn-OBn groups relative to phenol ring plane	G (Hartree)	ΔG of coordination reaction (kcal mol^{-1})
**L-LA**				−534.197188	
**THF**				−232.315175	
**5’-1**	*R,R*	2	Opposite	−6837.020241	+0.0 (reference)
**5’-1-LLA**	*R,R*	2	Opposite	−7440.782469	
**5’-1-LLA + 2 THF – 2 L-LA**				−6837.018443	+1.1
**5’-4**	*R,R*	1	Opposite	−6604.715559	+0.0 (reference)
**5’-4-LLA**	*R,R*	1	Opposite	−6906.597775	
**5’-4-LLA + THF – L-LA**				−6604.715762	−0.1
**5’-5**	*R,S*	2	Same	−6837.009322	+0.0 (reference)
**5’-5-LLA**	*R,S*	2	Same	−7440.774674	
**5’-5-LLA + 2 THF – 2 L-LA**				−6837.010648	-0.8
**5’-6**	*R,S*	1	Same	−6604.703344	+0.0 (reference)
**5’-6-LLA**	*R,S*	1	Same	−6906.59384	
**5’-6-LLA + THF – L-LA**				−6604.711827	−5.3

In terms of free enthalpy, the results in Table S11 suggest that the coordination of L-LA molecules to complex **5** varies from almost neutral to slightly favoured for coordination of one L-LA molecule (–5.3 kcal mol^{-1}), depending on the complex geometry and the number of THF molecules being replaced by L-LA molecules.

Table S12. Comparative free enthalpies of various structures of complex **5'-n-LLA** computed at the rωB97XD/6-311++g(d,p)/6-31g(d)/cpcm=tetrahydrofuran/298K level of theory.

**Structure**	**Stereochemistry at the nitrogen atoms**	**L-LA coordinated to Na**	**Position of Zn-OBn groups relative to phenol ring plane**	**G (Hartree)**	**ΔG of coordination reaction (kcal mol^{-1})**
**L-LA**				–534.197188	
**5'-1-LLA**	*R,R*	2	Opposite	–7440.782469	
**5'-1-LLA – L-LA**				–6837.018443	+7.8
**5'-4-LLA**	*R,R*	1	Opposite	–6906.597775	+0.0 (reference)
**5'-5-LLA**	*R,S*	2	Same	–7440.774674	
**5'-5-LLA – L-LA**				–6906.577486	+12.7
**5'-6-LLA**	*R,S*	1	Same	–6906.59384	+2.5

According to the results in Table S12, the most stable geometry for complex **5'-n-LLA** features one molecule of L-LA, (*R*,*R*) stereochemistry at the pyrrolidine nitrogen atoms of the ligand and the Zn-OBn groups facing opposite sides relative to the phenol ring plane (structure **5'-4-LLA**). However, the rearrangement of the ligand to a *meso* configuration (*R*,*S*) with Zn-OBn groups facing in the same direction relative to the phenol ring plane and coordination of one L-LA molecule (structure **5'-6-LLA**) may also be accessible under the polymerisation conditions as this structure is only slightly endergonic by 2.5 kcal mol^{-1} compared to ground state **5'-4-LLA**. The molecular structure with the lowest free enthalpy computed, **5'-4-LLA**, is shown in Figure S68.

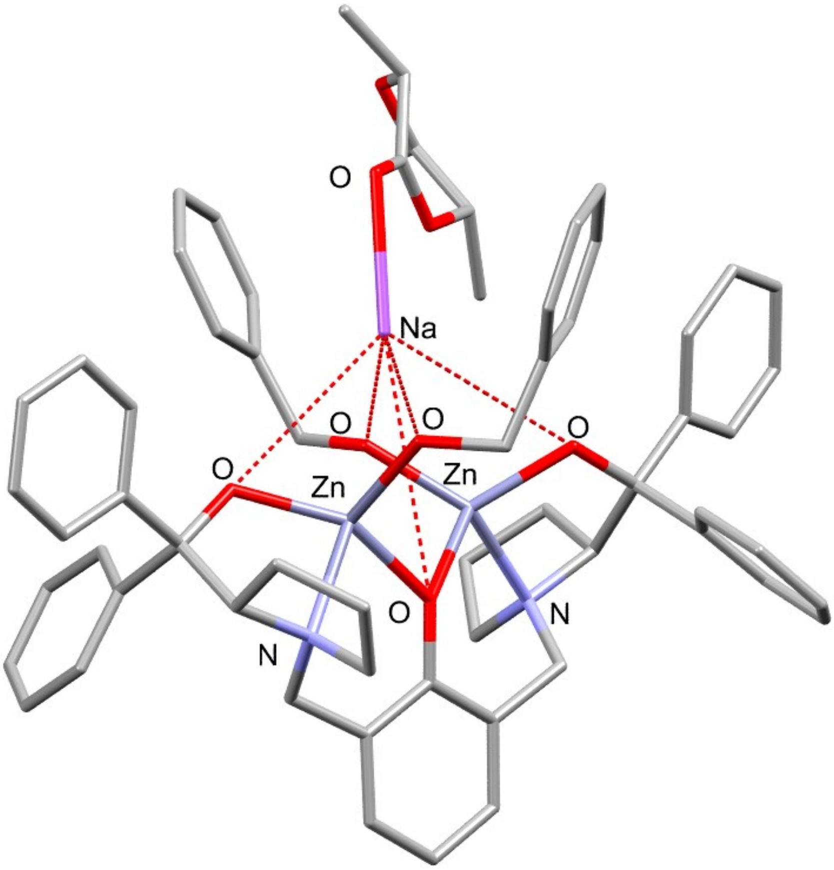

Figure S68. Molecular structure of complex **5’-4-LLA**, computed at the rωB97XD/6-311++g(d,p)/6-31g(d)/cpcm=tetrahydrofuran/298K level of theory.

Computed molecular structures of complex 4’

Several possible molecular structures of complex **4’** were optimised and their relative free enthalpies were compared. Based on the data obtained for **3’**, particular focus was given to the stereochemistry at the pyrrolidine nitrogen atoms as well as the coordination of one or two THF molecules to the alkali metal.

Table S13. Free enthalpies computed for various conformers of complex **4'** at the rωB97XD/6-311++g(d,p)/6-31g(d)/cpcm=tetrahydrofuran/298K level of theory.

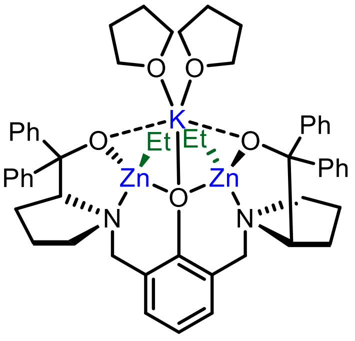

4'-1

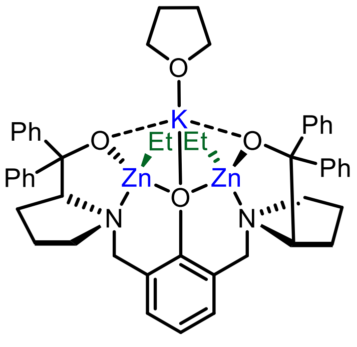

4'-4

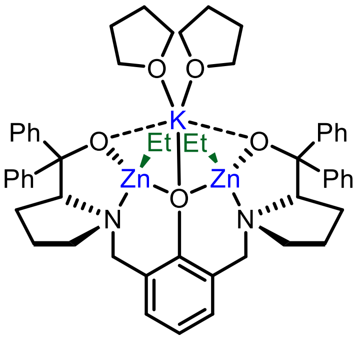

4'-5

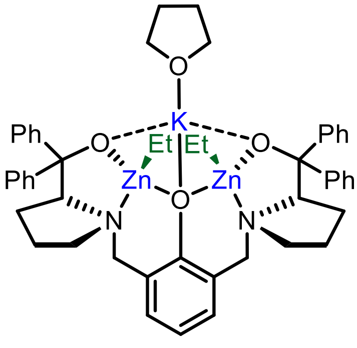

4'-6

Structure	Stereochemistry at the nitrogen atoms	THF coordinated to K	Position of Zn-Et groups relative to phenol ring plane	G (Hartree)	ΔG (kcal mol^{-1})
**4'-1**	*R,R*	2	Opposite	–6740.917915	+0.0 (reference)
**4'-4**	*R,R*	1	Opposite	–6508.600588	
**THF**				–232.315175	
**4'-4 + THF**				–6740.915763	+1.4
**4'-5**	*R,S*	2	Same	–6740.905574	+7.7
**4'-6**	*R,S*	1	Same	–6508.582947	
**4'-6 + THF**				–6740.898122	+12.4

The results in Table S13 suggest that a couple of structures (**4'-1** and **4'-4**) are within 2 kcal mol^{-1} of each other, and therefore represent plausible structures for complex **4** in solution. The *R*,*R* ligand configuration at the pyrrolidine nitrogen atoms, as seen in the solid-state for **1**, is likely retained upon coordination of Zn_2Et_2 in **4'**, with the coordination of two THF molecules slightly favoured over the coordination of one THF molecule. The molecular structure with the lowest free enthalpy computed, **4'-1**, is displayed in Figure S69.

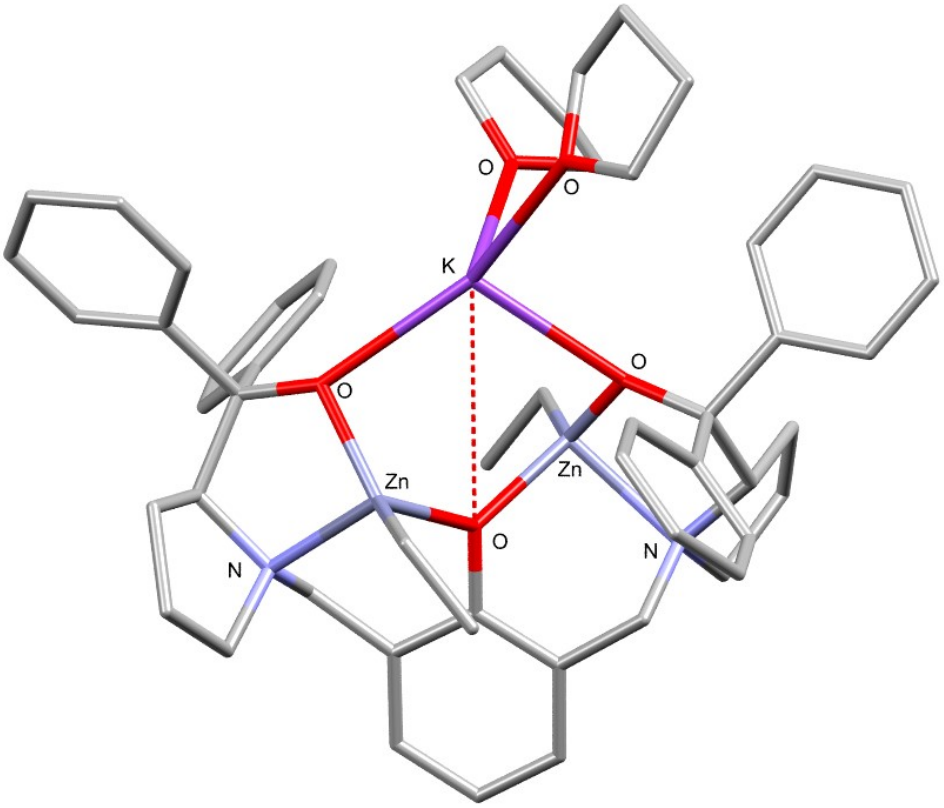

Figure S69. Molecular structure of complex **4’-1**, computed at the rωB97XD/6-311++g(d,p)/6-31g(d)/cpcm=tetrahydrofuran/298K level of theory.

Computed molecular structures of complex 6’

Several possible molecular structures of complex **6’** were optimised and their relative free enthalpies were compared. Based on the data obtained for complex **5’**, particular focus was given to the investigation of the stereochemistry at the pyrrolidine nitrogen atoms as well as the coordination of one or two THF molecules to the alkali metal.

Table S14. Free enthalpies computed for various conformers of complex **6’** at the rωB97XD/6-311++g(d,p)/6-31g(d)/cpcm=tetrahydrofuran/298K level of theory.

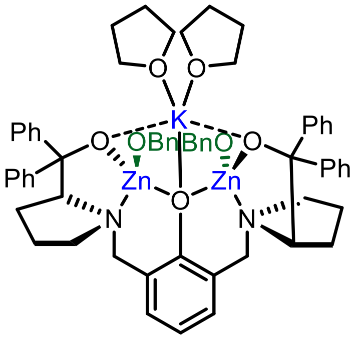

6'-1

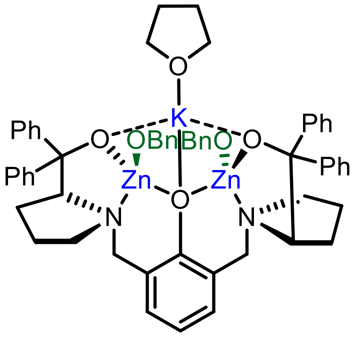

6'-4

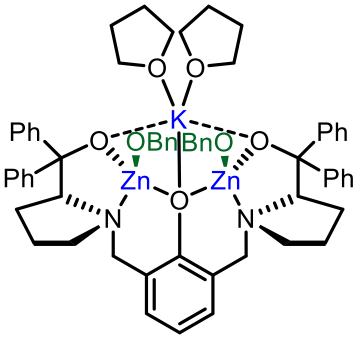

6'-5

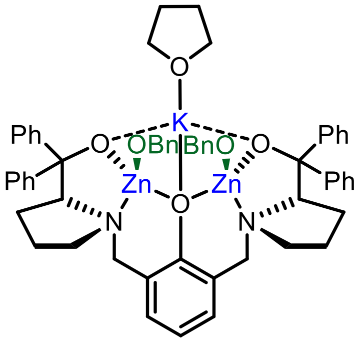

6'-6

Structure	Stereochemistry at the nitrogen atoms	THF coordinated to K	Position of Zn-Et groups relative to phenol ring plane	G (Hartree)	ΔG (kcal mol^{-1})
**6’-1**	*R*,*R*	2	Opposite	– 7274.678453	+0.0 (reference)
**6’-4**	*R*,*R*	1	Opposite	–7042.361021	
**THF**				–232.315175	
**6’-4 + THF**				–7274.676196	+1.4
**6’-5**	*R*,*S*	2	Same	-7274.654501	+15.0
**6’-6**	*R*,*S*	1	Same	–7042.341688	
**6’-6 + THF**				–7274.656863	+13.5

The calculations detailed in Table S14 suggest that structure **6’-1** has the lowest free enthalpy and therefore represents a plausible structure for complex **6** in solution. The *R*,*R* configuration of the ligand at the pyrrolidine nitrogen atoms, as seen in the solid-state for **1**, is likely retained upon reaction of **4’** with 2 eq. BnOH, with the coordination of two molecules of THF favoured over the coordination of one THF molecule (by 1.4 kcal mol^{-1}). The molecular structure with the lowest free enthalpy computed, **6’-1**, is shown in Figure S70.

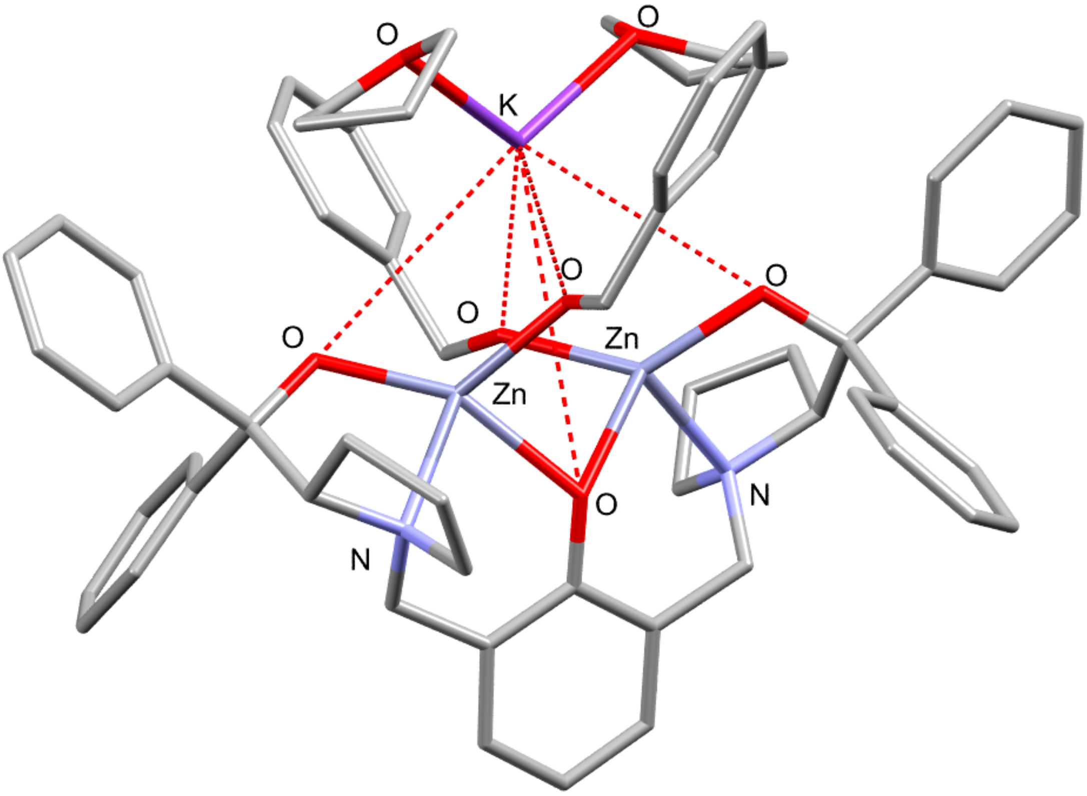

Figure S70. Molecular structure of complex **6'-1**, computed at the rωB97XD/6-311++g(d,p)/6-31g(d)/cpcm=tetrahydrofuran/298K level of theory.

Coordination of L-LA to complex 6' and molecular structures of the resulting complexes

Several possible molecular structures of complex **6'** coordinated to L-LA were optimised and their relative free enthalpies were compared. In particular, the relative position of the Zn-OBn groups, the stereochemistry at the pyrrolidine nitrogen atoms as well as the coordination of one or two L-LA molecules to the alkali metal were investigated.

Table S15. Free enthalpies computed for various structures of complex **6’-n-LLA** at the rωB97XD/6-311++g(d,p)/6-31g(d)/cpcm=tetrahydrofuran/298K level of theory.

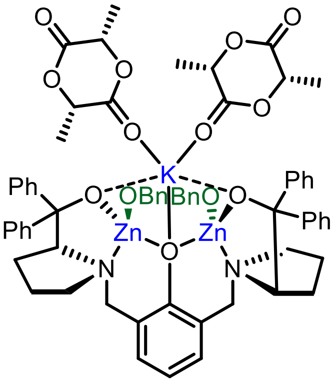

6'-1-LLA

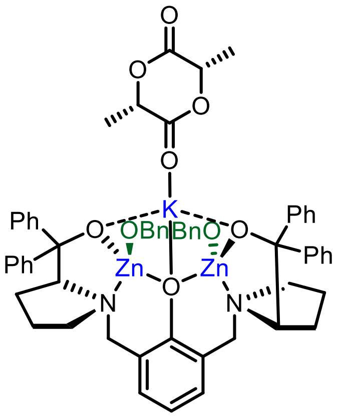

6'-4-LLA

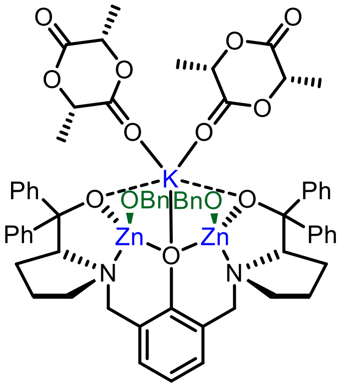

6'-5-LLA

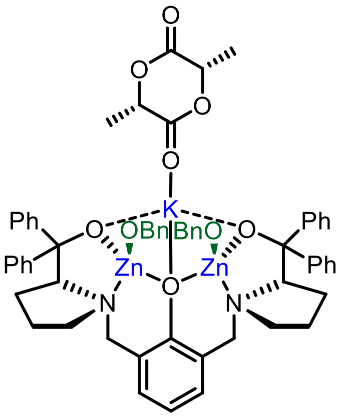

6'-6-LLA

Structure	Stereochemistry at the nitrogen atoms	THF or L-LA coordinated to K	Position of Zn-OBn groups relative to phenol ring plane	G (Hartree)	ΔG of coordination reaction (kcal mol^{-1})
**L-LA**				−534.197188	
**THF**				−232.315175	
**6’-1**	*R,R*	2	Opposite	−7274.678453	+0.0 (reference)
**6’-1-LLA**	*R,R*	2	Opposite	−7878.429952	
**6’-1-LLA + 2 THF – 2 L-LA**				−7274.665926	+7.8
**6’-4**	*R,R*	1	Opposite	−7042.361021	+0.0 (reference)
**6’-4-LLA**	*R,R*	1	Opposite	−7344.240917	
**6’-4-LLA + THF – L-LA**				−7042.358904	+1.3
**6’-5**	*R,S*	2	Same	−7274.654501	+0.0 (reference)
**6’-5-LLA**	*R,S*	2	Same	– 7878.433431	
**6’-5-LLA + 2 THF – 2 L-LA**				−7274.669405	−9.4
**6’-6**	*R,S*	1	Same	−7042.341688	+0.0 (reference)
**6’-6-LLA**	*R,S*	1	Same	– 7344.235003	
**6’-6-LLA + THF – L-LA**				−7042.35299	−7.1

The results in Table S15 suggest that the coordination of L-LA molecules to complex **6’** varies from almost neutral in term of free enthalpy to favoured (–9.4 kcal mol^{-1}), depending on the starting geometries of complex **6’** and the number of THF molecules being replaced by L-LA molecules.

Table S16. Comparative free enthalpies of various structures of complex 6’-n-LLA computed at the rωB97XD/6-311++g(d,p)/6-31g(d)/cpcm=tetrahydrofuran/298K level of theory.

Structure	Stereochemistry at the nitrogen atoms	L-LA coordinated to K	Position of Zn-OBn groups relative to phenol ring plane	G (Hartree)	ΔG of coordination reaction (kcal mol^{-1})
**L-LA**				–534.197188	
**6’-1-LLA**	*R,R*	2	Opposite	–7274.665926	
**6’-1-LLA – L-LA**				–7344.232764	+5.1
**6’-4-LLA**	*R,R*	1	Opposite	–7344.240917	+0.0 (reference)
**6’-5-LLA**	*R,S*	2	Same	– 7878.433431	
**6’-5-LLA – L-LA**				–7344.236243	+2.9
**6’-6-LLA**	*R,S*	1	Same	– 7344.235003	+3.7

Based on the results in Table S16, the most stable geometry of **6’-n-LLA** features one L-LA molecule coordinated, the ligand with (*R*,*R*) stereochemistry at the pyrrolidine nitrogen atoms and the two Zn-OBn moieties facing opposite sides relative to the phenol ring plane. However, it is plausible that the ligand rearranges to a *meso* configuration (*R*,*S*) with Zn-OBn groups facing in the same direction relative to the phenol ring under the polymerisation conditions, allowing coordination of two L-LA molecules (structure **6’-6-LLA**) as this structure is only slightly endergonic by 2.9 kcal mol^{-1} compared to ground state **6’-4-LLA**. The molecular structure with the lowest free enthalpy computed, **6’-4-LLA**, is shown in Figure S71.

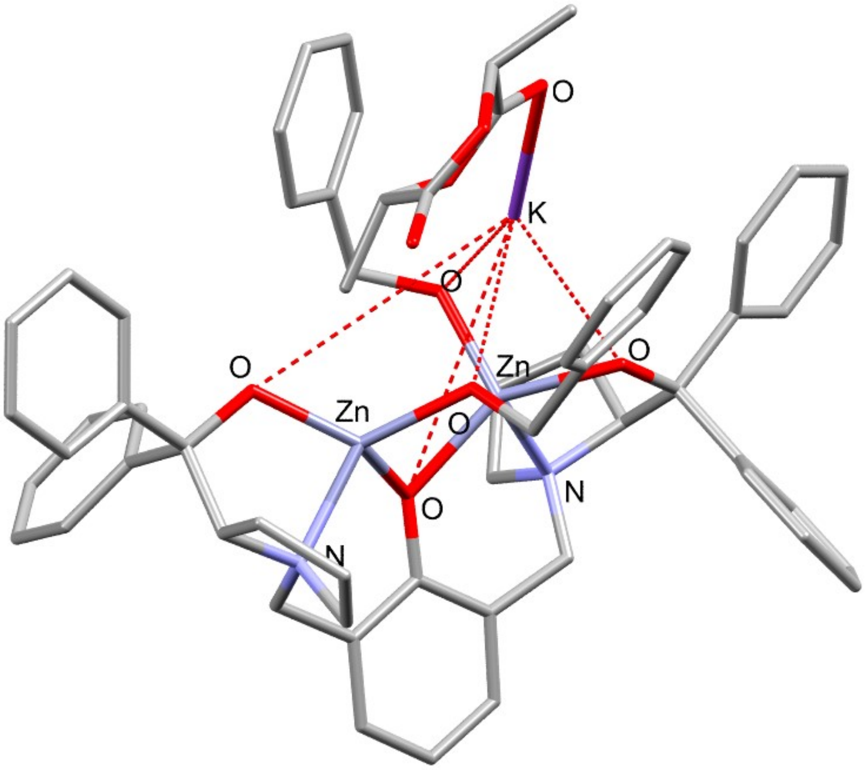

Figure S71. Molecular structure of complex 6'-4-LLA, computed at the rωB97XD/6-311++g(d,p)/6-31g(d)/cpcm=tetrahydrofuran/298K level of theory.

Comparison between DFT and X-ray data

To confirm that the DFT structures of **2'-6'** accurately represent metal complexes **2-6** based on the ProPhenol ligand (**LH_3**), a DFT structure of **[LZn_2(4-nitrophenol)]** was calculated (Figure S72) using the same computational parameters as were employed to calculate structures of complexes **2'-6'**. Complex **LZn_2(4-nitrophenol)]** was previously crystallised by Ding *et al.*,[10] enabling comparison of the key bond lengths and angles between the DFT and X-ray structures, as listed in Tables S17-18. The close correlation between the DFT and X-ray bond lengths and angles of **LZn_2(4-nitrophenol)]** confirm that that the DFT protocol selected was adequate to model the structure of complexes **2'-6'**.

Table S17. Comparison of selected bond lengths in DFT and X-ray structures of [LZn_2(4-nitrophenol)].

Selected bonds	Bond length in the DFT structure (Å)	Bond length in the X-ray structure (Å)[10]	% Difference
**Zn1-O5**	1.931	1.902(5)	1.5
**Zn1-O3**	2.025	2.005(6)	1.0
**Zn1-O4**	2.103	2.081(5)	1.1
**Zn2-O3**	2.028	2.000(6)	1.4
**Zn2-O6**	1.929	1.896(5)	1.7
**Zn2-O4**	2.107	2.091(5)	0.8
**Zn1-O7**	2.080	2.096(6)	0.8
**Zn2-O8**	2.079	2.096(6)	0.8
**Zn1-N11**	2.235	2.190(6)	2.0
**Zn2-N12**	2.229	2.203(6)	2.0

Literature X-ray atom labels were altered to match the DFT structure (Figure S72).

Table S18. Comparison of selected bond angles in DFT and X-ray structures of [LZn_2(4-nitrophenol)].

Selected bond angles	Bond angle in the DFT structure (°)	Bond angle in the X-ray structure (°)[10]	% Difference
**O3-Zn1-O4**	77.77	77.9(2)	0.2
**O5-Zn1-O7**	107.58	117.7(3)	8.6
**O3-Zn2-O4**	77.60	77.8(2)	0.3
**O6-Zn2-O8**	106.94	118.0(3)	9.4
**Zn1-O3-Zn2**	105.02	105.1(3)	0.1
**Zn1-O4-Zn2**	99.61	99.3(2)	0.3

Literature X-ray atom labels were altered to match the DFT structure (Figure S72).

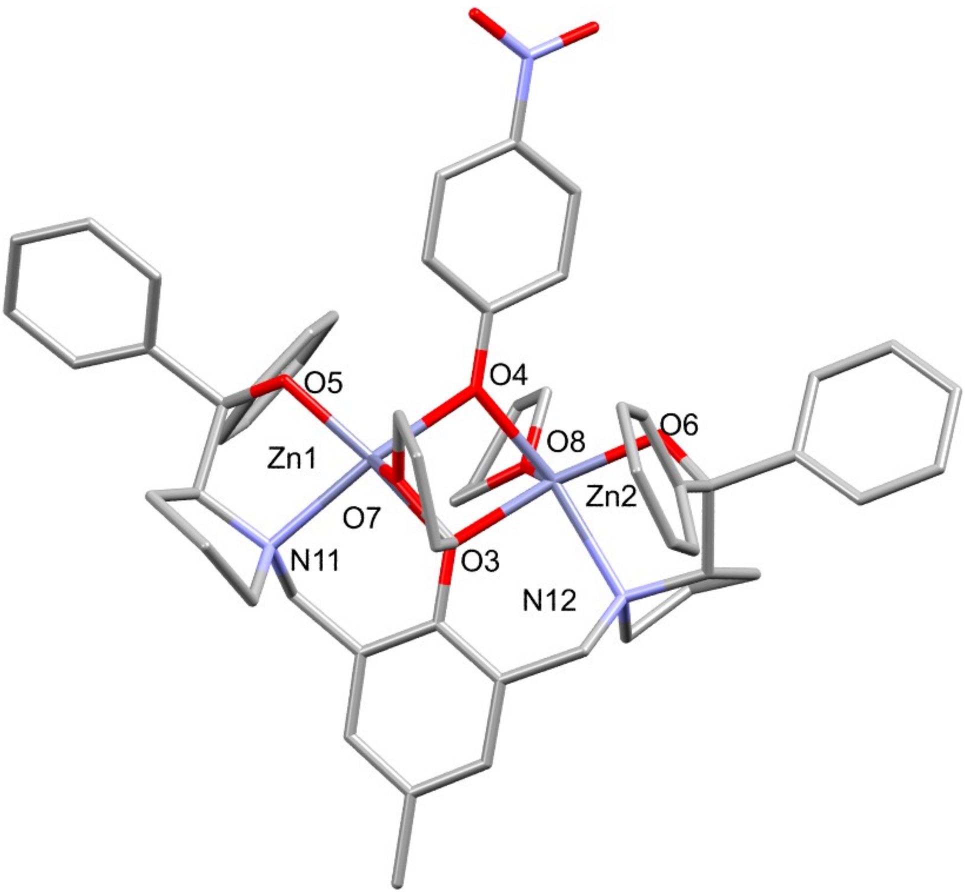

Figure S72. Molecular structure of complex [LZn_2(4-nitrophenol)], computed at the rωB97XD/6-311++g(d,p)/6-31g(d)/cpcm=tetrahydrofuran/298K level of theory.

References

1. W. Gruszka, L. C. Walker, M. P. Shaver, J. A. Garden, *Macromolecules*, 2020, **53**, 4294-4302.

2. G. M. Sheldrick, *Acta Crystallogr. Sect. C Struct. Chem.*, 2015, **71**, 3–8.

3. O. V. Dolomanov, L. J. Bourhis, R. J. Gildea, J. A. K. Howard, H. Puschmann, *J. Appl. Crystallogr.*, 2009, **42**, 339–341.

4. Gaussian 09, Revision D.01, M. J. Frisch, G. W. Frucks, H. B. Schlegel, G. E. Scuseria, M. A. Robb, J. R. Cheeseman, G. Scalmani, V. Barone, B. Mennucci, G. A. Petersson, H. Nakatsuji, M. Caricato, X. Li, H. P. Hratchian, A. F. Izmaylov, J. Bloino, G. Zheng, J. L. Sonnenberg, M. Hada, M. Ehara,K. Toyota, R. Fukuda, J. Hasegawa, M. Ishida, T. Nakajima, Y. Honda, O. Kitao, H. Nakai, T. Vreven, J. Montgomery, J. A.;, J. E. Peralta, F. Ogliaro, M. Bearpark, J. J. Heyd, E. Brothers, K. N. Kudin, V. N. Staroverov, R. Kobayashi, J. Normand, K. Raghavachari, A. Rendell, J. C. Burant, S. S. Iyengar, J. Tomasi, M. Cossi, N. Rega, J. M. Millam, M. Klene, J. E. Knox, J. B. Cross, V. Bakken, C. Adamo, J. Jaramillo, R. Gomperts, R. E. Stratmann, O. Yazyev, A. J. Austin, R. Cammi, C. Pomelli, J. W. Ochterski, R. L. Martin, K. Morokuma, V. G. Zakrzewski, G. A. Voth, P. Salvador, J. J. Dannenberg, S. Dapprich, A. D. Daniels, Ö. Farkas, J. B. Foresman, J. V. Ortiz, J. Cioslowski and D. J. Fox, Gaussian, Inc., Wallingford CT, 2009.

5. J. -D. Chai, M. Head-Gordon, *Chem. Phys. Lett.*, 2008, **467**, 176–178.

6. J. -D. Chai, M. Head-Gordon, *Phys. Chem. Chem. Phys.*, 2008, **10**, 6615–6620.

7. G. Scalmani, M. J. Frisch, *J. Chem. Phys.*, 2010, **132**, 114110-114115.

8. D. M. York, M. Karplus, *J. Phys. Chem. A*, 1999, **103**, 11060–11079.

9. A. Kowalski, A. Duda, S. Penczek, *Macromolecules,* 1998, **31**, 2114–2122.

10. Y. Xiao, Z. Wang, K. Ding, *Chem. Eur. J.*, 2005, **11**, 3668–3678.
